# Enzymatic elaboration of oxime-linked glycoconjugates in solution and on liposomes†

**DOI:** 10.1039/d2tb00714b

**Published:** 2022-06-14

**Authors:** Joana Silva, Reynard Spiess, Andrea Marchesi, Sabine L. Flitsch, Julie E. Gough, Simon J. Webb

**Affiliations:** Department of Chemistry, University of Manchester Oxford Road Manchester M13 9PL UK S.Webb@manchester.ac.uk +44 (0)-161-306-4524; Manchester Institute of Biotechnology, University of Manchester 131 Princess St Manchester M1 7DN UK; Department of Materials and Henry Royce Institute, The University of Manchester Manchester M13 9PL UK

## Abstract

Oxime formation is a convenient one-step method for ligating reducing sugars to surfaces, producing a mixture of closed ring α- and β-anomers along with open-chain (*E*)- and (*Z*)-isomers. Here we show that despite existing as a mixture of isomers, *N*-acetylglucosamine (GlcNAc) oximes can still be substrates for β(1,4)-galactosyltransferase (β4GalT1). β4GalT1 catalysed the galactosylation of GlcNAc oximes by a galactose donor (UDP-Gal) both in solution and *in situ* on the surface of liposomes, with conversions up to 60% in solution and *ca.* 15–20% at the liposome surface. It is proposed that the β-anomer is consumed preferentially but long reaction times allow this isomer to be replenished by equilibration from the remaining isomers. Adding further enzymes gave more complex oligosaccharides, with a combination of α-1,3-fucosyltransferase, β4GalT1 and the corresponding sugar donors providing Lewis X coated liposomes. However, sialylation using *T. cruzi* trans-sialidase and sialyllactose provided only very small amounts of sialyl Lewis X (sLe^x^) capped lipid. These observations show that combining oxime formation with enzymatic elaboration will be a useful method for the high-throughput surface modification of drug delivery vehicles, such as liposomes, with cell-targeting oligosaccharides.

## Abstract 2

Despite existing as a mixture of isomers, reducing sugar oximes can still be substrates for glycosyltransferases.

## Graphical abstract

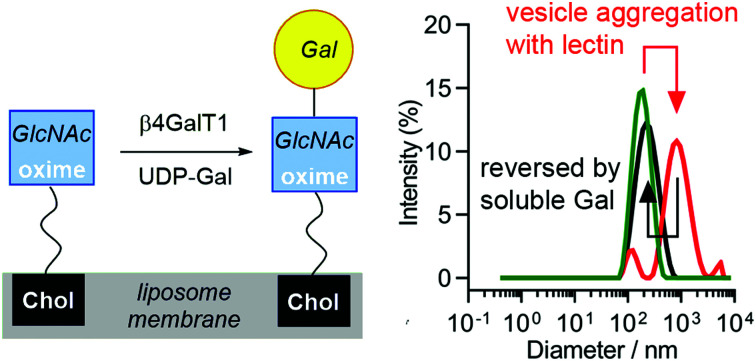

## Introduction

Oligosaccharide-coating of drug delivery vehicles is an attractive method for targeting particular cell types.^1^ For example, oligosaccharide-coated liposomes and nanoparticles have been developed that target sialoadhesin (Siglec-1, CD169), an endocytic surface receptor that preferably binds Neu5Ac(α2–3)Gal(β1–4)GlcNAc sequences.^2^ Indeed, since some sialic-acid-binding immunoglobulin-like lectins (Siglecs) can be overexpressed in diseased cells,^3^ they are attractive targets for drug delivery by coated liposomes. Similarly, the CD62E (E-selectin) interaction with sialyl Le^X^ can be used to target drug carrying liposomes to cancerous cells.^4^

**Fig. 1:**
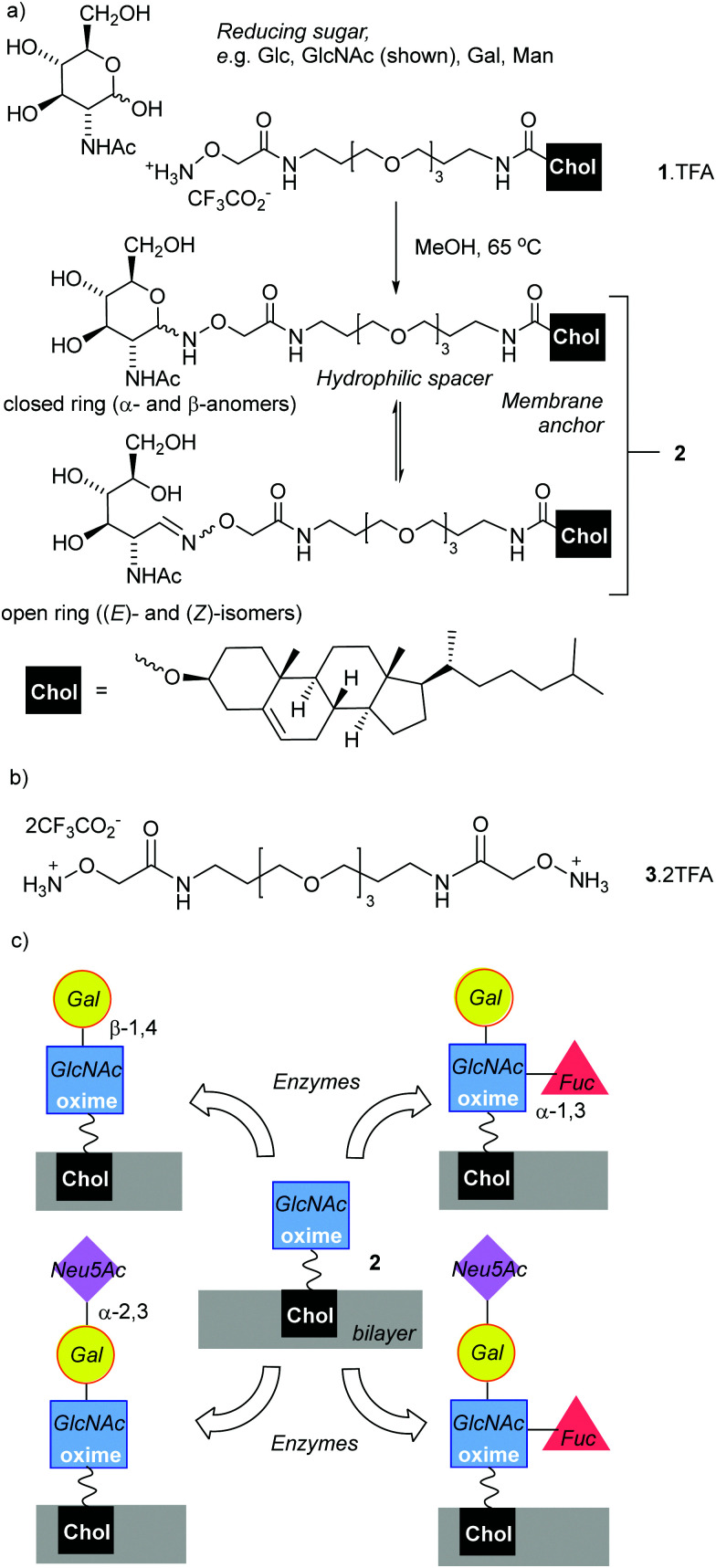
(a) Condensation of reducing sugars with *N*-(alkyloxy)amine terminated lipid 1 to create synthetic glycolipid 2 as a mixture of ring-closed and open-chain isomers. (b) Dimeric *N*-alkoxyammonium 3. (c) Proposed *in situ* enzymatic transformation of 2 into new synthetic glycoconjugates. Chol = cholesteryl-O-.

A key challenge is the development of rapid and cost-effective methods for attaching oligosaccharides to the surface of liposomes (phospholipid vesicles). The chemical synthesis of some oligosaccharides can be demanding and expensive,^5^ requiring time-consuming protection/deprotection strategies that lower yields. Adding chemoenzymatic strategies to chemical synthesis methodologies could shorten synthesis times and improve selectivity. The ligation of reducing sugars to hydrazides^6–8^ or *N*-alkoxyamines^9–12^ are versatile bioconjugation strategies,^13–15^ with the resulting adducts used for microarray platforms,^16,17^ vaccines,^18–20^ imaging,^21^ functionalising nanoparticles^22,23^ and scaffolds.^24,25^ These condensation reactions have the advantage of requiring only unprotected reducing sugars. These are available from the natural pool and are often the cheapest way of accessing key chemical motifs. The resulting adducts, hydrazones and oximes respectively, have significantly different structures and stabilities. Hydrazone adducts often ring close to form cyclic glycopyranoses,^6^ which structurally mimic the ring-closed forms (both α and β anomers) of the natural sugars. The oxime adducts on the other hand can exist as a mixture of ring-closed forms (α- and β-anomers), along with the (*E*)- and (*Z*)-isomers of the ring-opened oximes (Fig. 1a).^26^ These ring-opened forms are structurally quite different to the natural sugars, which is reflected in negligible binding of the ring-opened forms to the matching lectins;^16^ nonetheless oximes have greater hydrolytic stability than the hydrazones, a useful property for materials that need extended shelf-lives. *N*-Methyl-alkoxyamines are an alternative to conventional *N*-alkoxyamines that are reported to give almost exclusively ring-closed forms.^27,28^ However, the reaction of secondary oxyamines with reducing sugars is also reported to be significantly slower and give lower yields (albeit with greater absolute amounts of cyclic adduct).^8^

Liposomal formulations need to be stable in buffered solutions for extended periods, so the hydrolytic stability of *N*-alkoxyoxime/reducing sugar adducts in aqueous solution is attractive despite the formation of mixtures of isomers.^29^ Desired cell-targeting properties might be maintained if the terminal sugars of the adducts are ring-closed, which may be achieved either by directly condensing reducing oligosaccharides with an *N*-alkoxyamine lipid or by using glycosyltransferases to build upon the oxime adducts.

Chemoenzymatic methods have been shown to provide highly selective and efficient routes to desired oligosaccharides,^30,31^ with some glycosyltransferases capable of modifying unnatural substrates either in solution or on surfaces.^32–38^ In a recent example, β(1,4)-galactosyltransferase (β4GalT1) and *T. cruzi* trans-sialidase (TcTS) were used in a one-pot *in situ* procedure to catalyse the transfer of first galactose (Gal) then *N*-acetylneuraminic acid (Neu5Ac) onto a synthetic *N*-acetylglucolipid embedded in phospholipid liposomes.^32^

Given the high selectivity of glycosyltransferases for building oligosaccharides,^39,40^ determining if these enzymes could act on simple oxime adducts was attractive. Yang and Cheng reported that β4GalT1 could galactosylate GlcNAc hydrazones linked to gold nanoparticles,^41^ while Prudden *et al* reported enzymatic fucosylation of *N*-glucosyl-*N*-methyl-*N*-alkoxyamines.^8*c*^ However there is a lack of reports of enzymatic transformations of sugar oximes formed from primary *N*-alkoxyamines. Such oximes have been used to functionalise biosurfaces with saccharides,^25^ giving materials that may be suitable for further modification through the *in situ* application of enzymes.^36,37^ It was hoped that the closed forms of the sugar oximes might be accepted as substrates by key enzymes, with equilibration between closed and open forms allowing the feedthrough of all oxime isomers into accepted substrate. In this study, we explore this combination of high-throughput chemical ligation with *in situ* multienzyme transformation as a pathway towards oligosaccharide-coated liposomes.

## Results and discussion

### Synthesis of *N*-alkoxyamines 1 and 3

To anchor oxime-glycolipids to the liposomal membrane, a cholesteryl anchor was selected (Fig. 1a).^42^ A reactive *N*-alkoxyamine terminus was linked to the cholesteryl unit through a triethyleneglycol (TEG) spacer, which was hoped to facilitate access of enzymes and lectins to the ligated sugars when the lipid is embedded in a membrane. The trifluoroacetate salt of *N*-alkoxyamine lipid 1 (Fig. 1a), which combines these features, was synthesised in 20% overall yield in three steps from commercial reagents (see the ESI†).

Lipid 1 is amphiphilic and does not have a distinct chromophore, which impairs quantitative analysis of product mixtures by both NMR spectroscopy and HPLC (UV detection). Therefore two water-soluble model compounds were used. Commercially available *N*-ethoxyamine 5 was used by Baudendistel *et al.* to quantify the complex equilibria that exist when condensing *N*-alkoxyamines with reducing sugars, such as glucose, mannose and *N*,*N*′-diacetylchitobiose.^9^ In addition, dimeric *N*-alkoxyammonium 3 (Fig. 1b) was synthesised. It has the same reactive terminus as 1 and permits the condensation methodology to be tested on this analogue in solution. The water-soluble oximes produced were used to validate our chemoenzymatic methodology.

### Condensation of reducing sugars with 1, 3 and 5

**Scheme 1:**
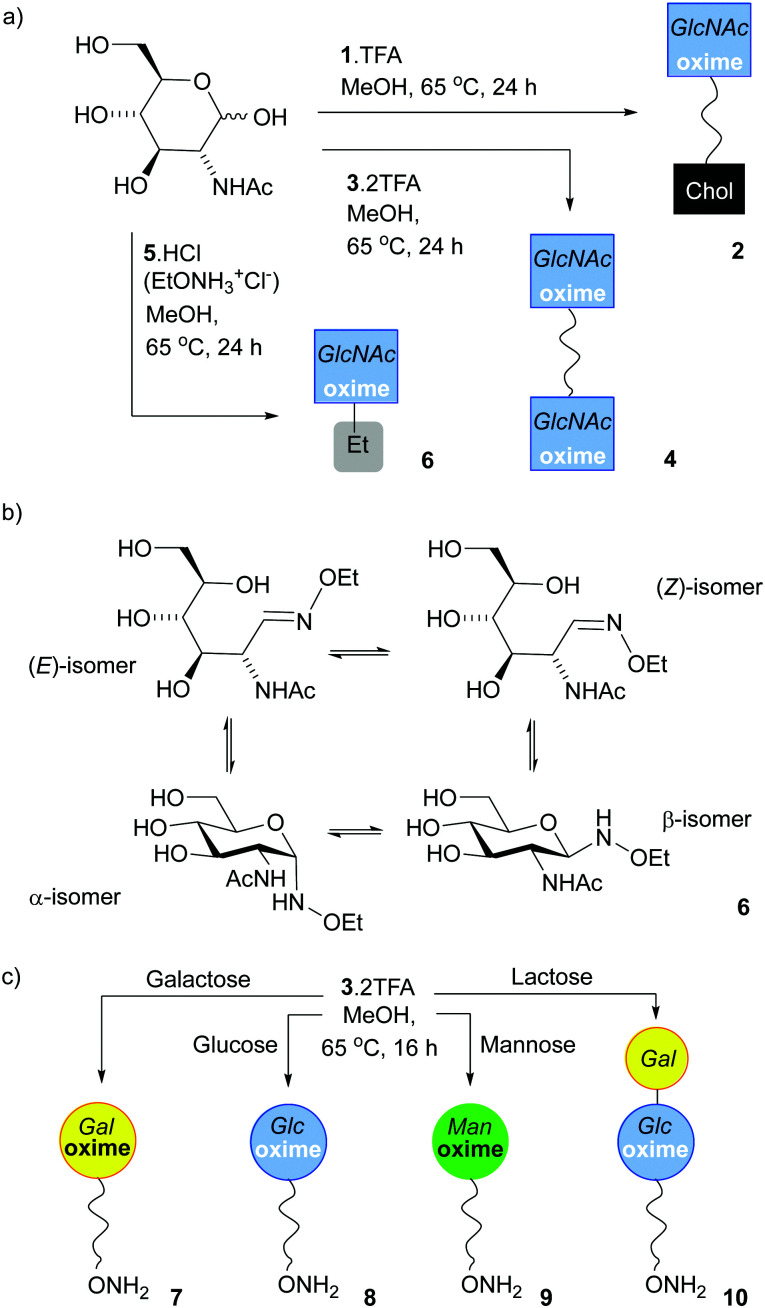
(a) Synthesis of 2, 4 and 6, the oxime adducts between GlcNAc and 1, 3 and 5 respectively, using the SNFG symbol for GlcNAc.^45,46^ (b) Structures of the four isomers found in 6. (c) Monoconjugation of 3 to reducing monosaccharides to make 7, 8, 9 and 10.

The reaction of 5·HCl (*N*-ethoxyammonium chloride) with GlcNAc (1.5 eq.) was studied first (Scheme 1). Several potential catalysts were screened^43,44^ but they did not improve the adduct yield when compared to simply heating the reactants together in methanol at 65 °C (see the ESI†), perhaps because 5·HCl is itself weakly acidic. ^1^H NMR spectroscopy on this crude mixture showed resonances at 7.42 and 6.72 ppm, which arise from the CH=N protons for the open-chain (*E*)- and (*Z*)-oximes respectively,^9^ whereas signals at 4.66 and 4.32 ppm are from the α- and β-anomers of the cyclic forms respectively. Integration of the peaks in the reaction mixture after 24 h gave an *E*/*Z*/α/β ratio of 5% : 3% : 30% : 61% (see the ESI†). This mixture was dissolved in CH_3_CN/water, then separated by HPLC to give a product-containing fraction in 46% yield (120 mg). Integration showed the *E*/*Z*/α/β isomer ratio was now 64% : 21% : 0% : 15% in CD_3_OD, which was a relatively consistent ratio between repeat reactions. Baudendistel *et al.* reported a 56% : 18% : 0% : 26% *E*/*Z*/α/β ratio for the same product in buffer at pH 5 at 21 °C.^9^

It was also possible to isolate an HPLC fraction enriched in the open-chain (*E*)-oxime (*E*/*Z*/α/β 94% : 4% : 0% : 3%), which allowed assessment of the interconversion rate between isomers. This (*E*)-oxime enriched mixture was dissolved in deuterated MES buffer (pD 7, 20 °C) and monitored by ^1^H NMR spectroscopy over time. After six days the (*E*)-oxime still predominated (61%) but smaller amounts of the (*Z*)-oxime (21%) and cyclic β-anomer (18%) were present; the α-anomer was not detected over this period (see the ESI†).

Much like 5·HCl, bivalent reactive tether 3 could be condensed with reducing sugars; heating 3·2TFA in methanol with 2 eq. of GlcNAc for 16 h provided 4, a tether displaying two saccharides, in 15% yield after HPLC separation. The *E*/*Z*/α/β ratio in 4 (Scheme 1a) was 58% : 23% : 0% : 19%, which was not significantly different to that observed for 6 (64% : 21% : 0% : 15%). Much like *N*-ethoxyamine,^9^ bivalent reactive tether 3 could be condensed with other simple sugars. In efforts to obtain a mono-functionalised tether that could be directly conjugated to a surface, the saccharide to tether ratio was decreased. Reaction with reducing sugars (0.5 eq.) in methanol at 65 °C for 16 h followed by HPLC separation afforded the following adducts (yields calculated from the sugar): 7 (Gal, 33%), 8 (Glc, 11%), 9 (Man, 12%) and 10 (Lac, 11%) (Scheme 1c). However, using this procedure with *N*-acetylglucosamine, fucose, glucosamine, glucose-6-phosphate, 2-deoxyglucose, *N*-acetyllactosamine and 3′-sialyllactose gave a mixture of mono- and double-substituted adducts that could not be separated by HPLC (see the ESI,† Section 4). Overall, these efforts to mono-functionalise 3 were too low yielding and time intensive to take forwards.

GlcNAc could be ligated onto lipid 1·TFA by heating in methanol under nitrogen overnight. Reasonable quantities of 2 (Scheme 1a) could be obtained (a yield of 49%) as the cholesterol tail allowed purification by normal phase column chromatography, although once again the isomers could not be separated. ^1^H NMR spectroscopy in CD_3_OD showed 2 was a mixture of (*E*)-oxime, (*Z*)-oxime, α-anomer and β-anomer in a respective ratio of 50% : 32% : 0% : 18%, rather similar to the ratio observed for 4. Similarly, a LacNAc adduct could be obtained that had a 67% : 27% : 0% : 24% ratio; in this case the adduct presents a Gal residue that is unmodified. This chemically obtained adduct proved to be a useful reference compound for monitoring the enzymatic galactosylation of 2 (Fig. 4). Unlike GlcNAc and LacNAc, condensation of 1 with 3-sialyllactose (3′-SL, Neu5Ac(α2–3)Gal(β1–4)GlcNAc) only provided a small amount of the 3′-SL adduct, with the major product a LacNAc adduct that resulted from fragmentation of the trisaccharide. This observation is similar to the unwanted production of a fucosyl adduct during the attempted ligation of sialyl Lewis X to an aryl hydrazide^6^ and shows that this chemical ligation methodology can fail with oligosaccharides containing sensitive groups.

### Enzymatic modification of soluble GlcNAc oximes by β4GalT1

#### Adduct 6 in buffer

**Fig. 2:**
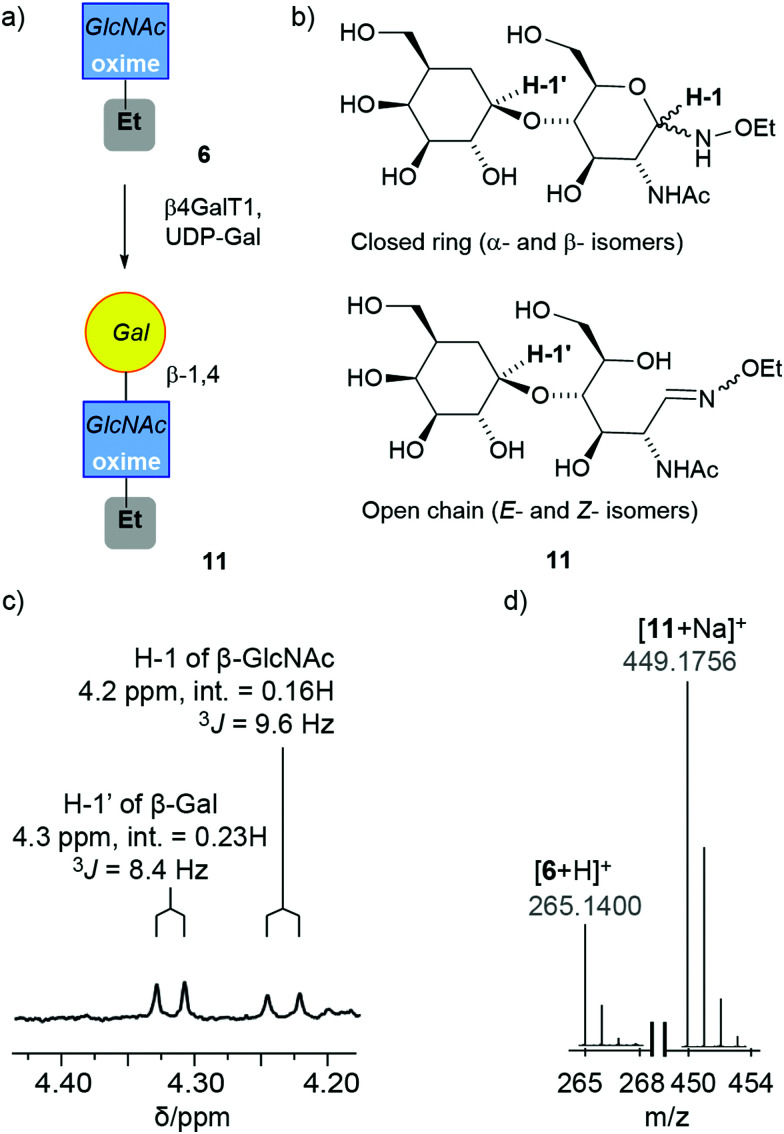
(a) Enzymatic synthesis of 11. This schematic representation uses a SNFG symbol to cover all four isomers in the mixture.^45,46^ (b) The four main isomers of 11. (c and d) Spectral data for the reaction mixture after purification by HPLC: (c) expansion of the ^1^H NMR spectrum of 11 in CD_3_OD showing anomeric protons for the Gal (H-1′) and the cyclic β-anomer of GlcNAc oxime (H-1), with integrations (int.) and coupling constants (*J*) indicated. (d) MS data showing the successful conversion of 6 into the adduct 11.

GlcNAc–NHOEt 6, comprising a mixture of open/closed chains (4.6 mg, *E*/*Z*/α/β ratio 64% : 21% : 0% : 15%) was subjected to standard transformation conditions using bovine β4GalT1 (UniProt number: P08037, expressed in *E. coli*) for 16 h in MES buffer pH 7.0 at 37 °C (Fig. 2a).^32^ This enzymatic transformation was followed by HPLC separation of the product-containing fraction from paramagnetic Mn(ii) ions. The product-containing fraction (3.0 mg, *E*/*Z*/α/β ratio 60% : 24% : 0% : 16%) contained significant amounts of starting material but showed 23% conversion to 11 (2.6 μmol). The β4GalT1 enzyme is reportedly specific toward β-linked GlcNAc acceptors,^47^ which suggests the extent of conversion is limited by the amount of β-anomer present in 6 (2.6 μmol). Since HPLC separation of 6 had also provided a mixture enriched in the closed ring configuration (*E*/*Z*/α/β ratio of 5% : 3% : 32% : 60%), this was also subjected to standard β4GalT1 enzymatic transformation conditions for 16 h. Consistent with the increased proportion of β-anomer, the resulting product mixture after 24 h contained 50% of the LacNAc adduct 11. The ^1^H NMR spectrum of the product-containing fraction showed the appearance of a doublet at 4.32 ppm that corresponds to the proton attached to the anomeric carbon of β-1,4-linked Gal. The integration of the anomeric proton on the GlcNAc relative to the anomeric proton Gal indicates that a significant proportion (70%) of GlcNAc is in the cyclic form when the Gal is ligated to it (Fig. 2c). Successful conversion to LacNAc–NHOEt 11 was also indicated by the observation of the product peak (449.1756 *m*/*z* for [11 + Na]^+^) in the positive ion electrospray LC-MS trace (Fig. 2d).

Given that six days were needed to produce β-anomer from open-chain (*E*)-oxime, extending the reaction time to six days was hoped to not only to give more time for the enzymatic reaction but also to provide more substrate that is accepted by the enzyme. A mixture enriched in the open-chain isomer (1.0 mg, *E*/*Z*/α/β ratio of 93% : 4% : 0% : 3%, see the ESI†) was subjected to standard β4GalT1 enzymatic transformation conditions for six days, then the reaction mixture separated by HPLC. The ^1^H NMR spectrum of the product-containing fraction (0.5 mg) showed a doublet arising from the anomeric proton of the β-1,4-linked Gal carbon in 11 and the product peak was found in the positive ion electrospray LC-MS trace (see the ESI†). The extent of conversion into the LacNAc adduct was calculated by integrating the ^1^H NMR spectrum (60%, ∼0.3 mg) and was much greater than the proportion of β-anomer in the starting substrate mixture (3%, ∼0.03 mg). This suggests that either the β-anomer was replenished by equilibration from the acyclic isomers or the open-chain oximes can be substrates.

These studies show that sugar oximes can be accepted as substrates by glycosyltransferases, although they are poorer substrates than the native reducing sugars. An alternative strategy of enzymatically modifying the native saccharide in solution before oxime ligation may be more efficient for simple or robust oligosaccharides, like LacNAc, although it may not be feasible for sensitive oligosaccharides that are prone to fragmentation.^6^

#### Adduct 4 in buffer

**Fig. 3:**
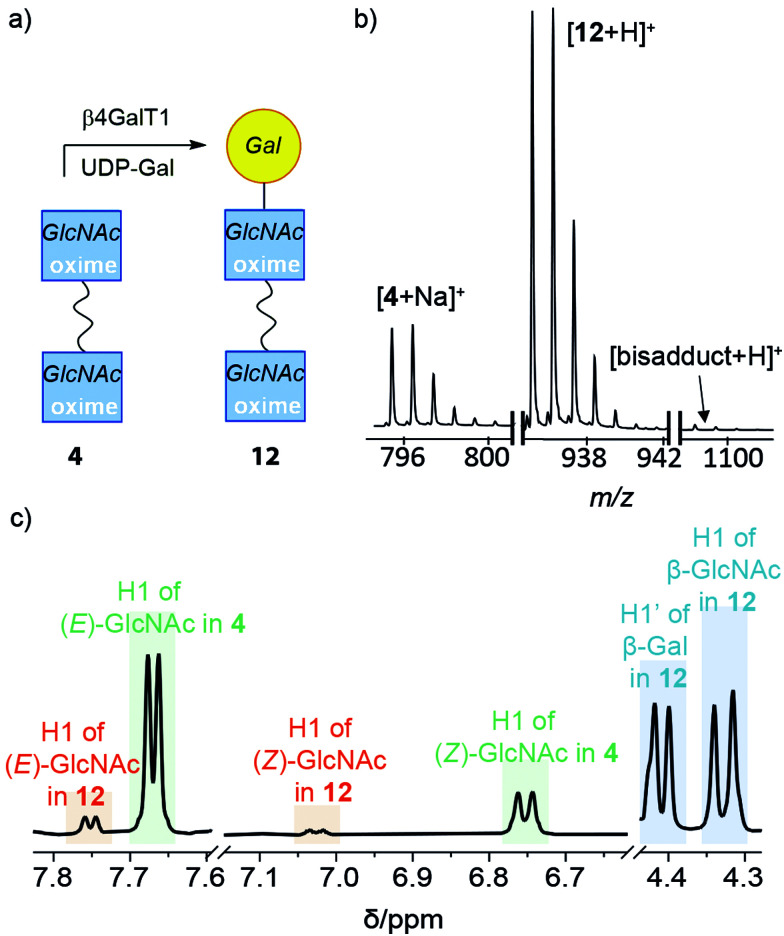
(a) Schematic representation of the enzymatic galactosylation of 4 showing the main product formed, 12. (b) Partial mass spectrum showing ions for 4, 12 and the bisadduct. (c) Expansion of the ^1^H NMR spectrum of 12 in CD_3_OD showing oxime protons for (*E*)- and (*Z*)-isomers of 4 and 12 as well as the anomeric protons for Gal (labelled as H1′) and cyclic β-anomer of GlcNAc (labelled as H1).

The effect of the TEG chain on the ability of β4GalT1 to transfer galactose onto GlcNAc was then assessed (Fig. 3a). Bivalent 4 was subjected to the same enzymatic transformation conditions as 6. A mixture of 4 (ratio of *E*/*Z*/α/β of 58% : 23% : 0% : 19%), UDP-Gal and β4GalT1 in pH 7.0 MES buffer was incubated overnight at 37 °C. The enzymatically transformed mixture was purified by HPLC to give a product-containing fraction with 70% mass recovery. ESI-MS indicated that most of the HPLC fraction was mono-LacNAc product (+Na^+^, 935 *m*/*z*) with a significant amount of unreacted starting material, and a very small amount of the di-LacNAc product (+ H^+^, 1097 *m*/*z*) (Fig. 3b). In the ^1^H NMR spectrum, a new resonance at 4.41 ppm indicated that Gal had been attached to 4 through a β(1–4) linkage (Fig. 3c). Integration of the signal indicated that 59% of the available GlcNAc groups had been transformed. Resonances corresponding to the (*E*)- and (*Z*)-oximes of the starting adduct 4 were found at 7.6 and 6.7 ppm, as well as the (*E*)- and (*Z*)-oximes of the mono-substituted LacNAc product 12, and 7.7 and 7.0 ppm, respectively. Interestingly, these resonances showed the proportion of open-chain oxime was lower in the product 12 than the starting material 4, consistent with the GlcNAc β-anomer being the preferred substrate for β4GalT1 (assuming product 12 has not yet equilibrated).^48^ A DOSY spectrum was able to discriminate between the starting materials and the two products (see the ESI†), with a slightly smaller diffusion constant observed for 12.

**Fig. 4:**
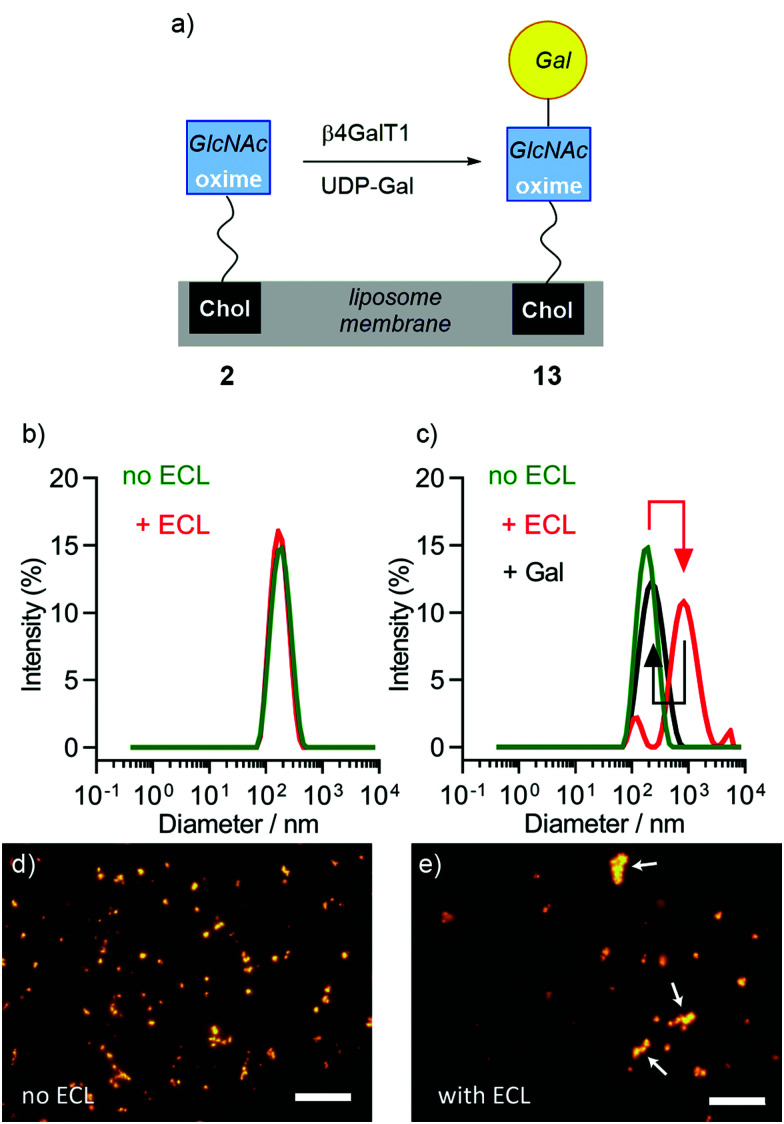
(a) Schematic representation of the *in situ* enzymatic galactosylation of liposomes bearing 2 to give liposomes bearing 13. (b–e) Changes observed upon addition of ECL lectin (0.1 mg mL^−1^) to DMPC liposomes either containing 2 (10 mol%) or a mixture of 2 and 13. (b) DLS of DMPC/2 liposomes gave *d* = 163.9 ± 2.8 nm and 169.9 ± 2.6 nm before (green trace) and after (red trace) addition of ECL. (c) DLS of DMPC/(2 + 13) liposomes gave *d* = 170.0 ± 2.5 nm (PdI of 0.1) and 611.0 ± 211.3 nm (PdI of 0.4) before (green trace) and after (red trace) addition of ECL. The increase in diameter was reversed upon addition of 200 mM galactose (black trace). (d and e) Fluorescence microscopy in HEPES buffer of DMPC/(2 + 13) liposomes (labelled with rhodamine DHPE) (d) before and (e) after addition of ECL. Liposome agglomerates are marked with white arrows. Scale bars each 20 μm.

The higher yield of product despite the shorter reaction time when compared to the analogous reaction involving 6 could imply that 4 is a better substrate for the enzyme (perhaps due to its bivalency)^34^ and/or isomer interconversion may be faster for this compound. Nonetheless, the successful transformation of 4 into 12 confirms that the TEG linker does not prevent the GlcNAc moiety from being a substrate for β4GalT1.

### Enzymatic modification of 2 in liposomes

GlcNAc lipid 2 is not soluble in buffer and forms large aggregates (see the ESI†), which prevents it from reacting in solution in the same way as 4. However, its amphiphilicity allows it to embed into phospholipid bilayers. For example, lipid 1 extensively partitioned into liposome membranes, leaving no detectable amine in solution (see the ESI†). Although the bilayer may present a steric barrier for enzymes, bovine β4GalT1 has been shown to transform *in situ* GlcNAc-capped lipids that are embedded in bilayers to give Gal-labelled liposomes that target human hepatocellular carcinoma (HepG2) cells.^32,49–51^

Liposomes (200 nm diameter) composed of 90 mol% 1,2-dimyristoyl-*sn-glycero*-3-phosphocholine (DMPC) and 2 (10 mol%, as a mixture of isomers) were created by extrusion through polycarbonate membranes with 200 nm diameter pores; this bilayer mixture and liposome size was selected to exploit the enhanced permeability and retention effect and to only require gentle heating during the extrusion process (*T*_m_(DMPC) = 24 °C).^32,52^ The 10 mol% doping level in DMPC/2 liposomes gave 0.2 mM 2 in a 2 mM final lipid concentration (1 mL MES buffer). Addition of the synthetic lipid made no difference to the size of the liposomes (DMPC alone, 163.0 ± 0.7 nm diameter; DMPC/2, 161.8 ± 1.2 nm diameter) and only a very small change to the zeta potential (in HEPES buffer pH 7.5: DMPC only −1.28 mV; DMPC/2, −1.53 mV. See the ESI†). These zeta potentials are comparable to reported values for DMPC liposomes in water and other buffers (−5 to −9 mV).^53,54^

The successful embedding of 2 in liposome membranes was confirmed by adding a GlcNAc-selective lectin, wheat germ agglutinin (WGA). Suspensions of DMPC liposomes that were either undoped or doped with 2 (DMPC/2 liposomes) were mixed with WGA in HEPES buffer (pH 7.5, with CaCl_2_ and NaCl). Carbohydrate-binding proteins, such as lectins, are reported to only recognise cyclic conjugates,^9,16^ so an increase in turbidity at 360 nm with WGA concentration suggested that sufficient closed-chain β-anomer was present to mediate liposome aggregation; no increase in turbidity was observed for undoped liposomes. DLS showed that average particle diameter changed from 159 nm to 31 μm after the addition of WGA (see the ESI†) and flocculation was observed. Fluorescence microscopy showed liposome agglutination in the presence of WGA (see the ESI†). This aggregation was reversible upon the addition of soluble GlcNAc (0.5 M), showing that a specific interaction between membrane-embedded 2 and the lectin is responsible for aggregation.

The same methodology employed to enzymatically modify 6 and 4 was then applied to DMPC/2 liposome suspensions (Fig. 4a). Aliquots of pre-extruded liposome suspensions (100 μL) were transferred to vials followed by the addition of UDP-Gal, MnCl_2_ and β4GalT1 enzyme, then the mixture incubated overnight (16 h). Either 37 °C or room temperature were found to give identical results, so the lower temperature was used as it is better for developing drug delivery systems; the rate of release of entrapped drugs from the liposome lumen can increase significantly with temperature.^55^

**Fig. 5:**
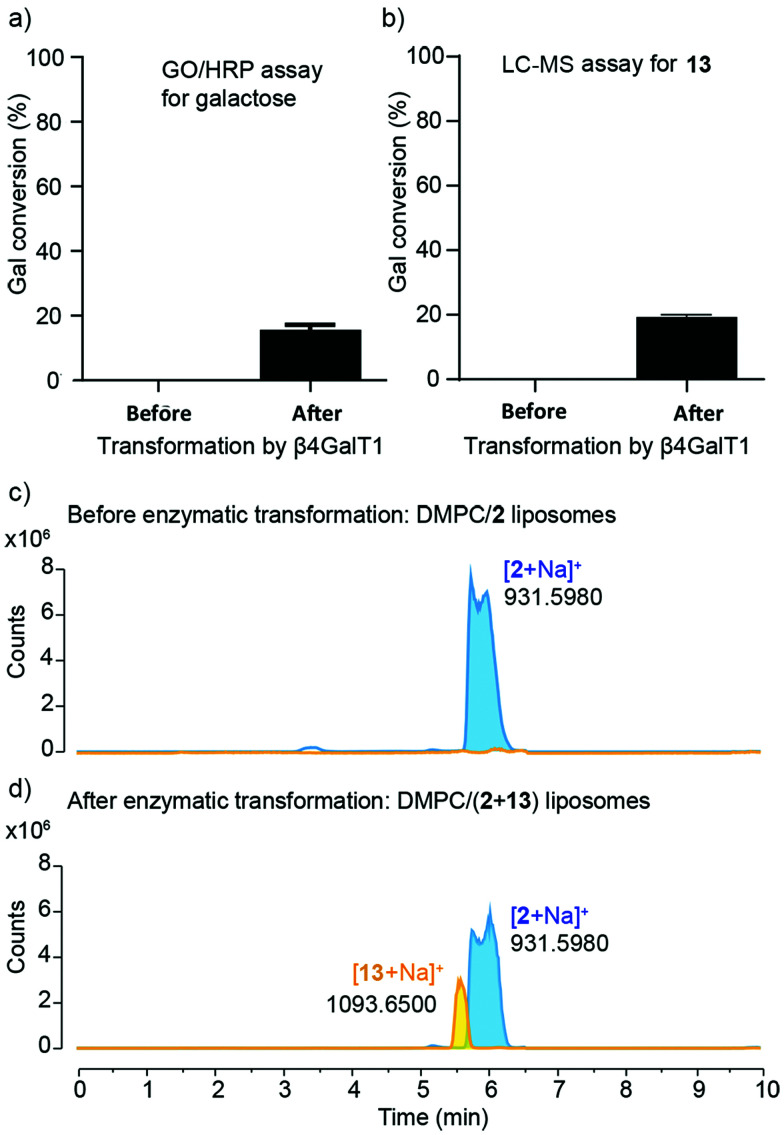
(a) Galactosylation, calculated from GO/HRP assays, in samples before and after treatment with β4GalT1, with conversion of 15.6 ± 1.1% in the latter. Data correspond to mean ± SD (*n* = 3). (b) Galactosylation of DMPC/13 liposomes, estimated using LC-MS, in samples before and after treatment with β4GalT1, with conversion of 19.0 ± 0.7% in the latter. Data correspond to mean ± SD (*n* = 6 from two independent experiments). (c and d) Extracted LC-MS chromatograms of (c) DMPC/2 liposomes (before enzymatic reaction) and (d) DMPC/(2 + 13) liposomes (after enzymatic reaction) showing masses for glycolipids 2 (6.0 min) and 13 (5.5 min).

The enzymatic transformation of the DMPC/2 liposomes to give DMPC/(2 + 13) vesicles was monitored both qualitatively (Fig. 4) and quantitatively (Fig. 5). Enzymatic conversion led to only a small change in the zeta potential, to +3.62 mV in HEPES buffer. Aggregation by *Erythrina cristagalli* lectin^56^ (ECL, in HEPES buffer pH 7.5 with CaCl_2_ and NaCl), which has been used previously to indicate the galactosylation of liposomes by β4GalT1,^34^ was confirmed by increases in turbidity at 360 nm as the concentration of ECL increased; no increase in turbidity was observed for untransformed liposomes. DLS confirmed the increase in particle diameter, from 170 nm before to 600 nm after ECL mediated aggregation (Fig. 4b and c). This aggregation was reversible upon addition of soluble Gal (0.2 M), which showed a specific lectin/Gal interaction was responsible. Fluorescence microscopy corroborated these measurements (Fig. 4d and e), although the number and size of the agglomerates produced by ECL were smaller than those observed for GlcNAc-coated liposomes mixed with WGA.

Quantitative measurement of the extent of galactosylation was then performed using the coupled galactose oxidase/horseradish peroxidase (GO/HRP) assay. The GO/HRP assay is not only able to detect Gal, but also galactosyl derivatives with a C6 hydroxyl, including Gal- or LacNAc-capped glycolipids.^57–59^ Since galactose oxidase selects for the hydroxyl orientation at C4, it does not oxidise Glc residues.^59^ Liposomal samples were analysed using a galactose assay kit (MAK012 from Sigma) according to the supplier's instructions. The fluorescence of the samples was measured (*λ*_ex_ = 535 nm and *λ*_em_ = 587 nm) and interpolated into a standard curve that used different concentrations of synthesised 13. These GO/HRP assays gave a conversion of (16 ± 1)% across three repeats of the transformation with bovine β4GalT1 (Fig. 5a). This assay also showed that human β4GalT1 (UniProt number: B2RAZ5) and bacterial lacto-*N*-neotetraose biosynthesis glycosyltransferase (LgtB, UniProt number: Q51116, expressed in *E. coli*)^60,61^ were similarly effective. The performance of all β4GalT1 variants was the same within the uncertainties of the assay, with 16% Gal conversion for bovine and human β4GalT1 enzyme and 15% for LgtB (see the ESI†).

Analysis by LC-MS, which showed fractions with masses corresponding to both 2 (*m*/*z* = 931.598) and the product 13 (*m*/*z* = 1093.651), supported these GO/HRP data. The extent of conversion was estimated by integration the ESI MS peak obtained from the LC-MS data (see the ESI†). To account for any effect of the phospholipid on ionisation, a calibration curve was constructed using mixtures of separate populations of liposomes containing either 2 or 13 (10 mol%), in the following proportions: 1 : 0, 3 : 1, 1 : 1, 1 : 3, 0 : 1. The data obtained was then analysed based on extracted chromatograms relative to 2 ([M + Na]^+^ 931.598) and 13 ([M + Na]^+^ 1093.651) masses; 13 eluted at 5.5 min and 2 eluted at 6.0 min (Fig. 5c and d). The area of each extracted chromatogram (in counts) was expressed as a ratio of 13 (EC_1093_) in terms of 2 (EC_931_). Interpolation of the integrated intensity of the ion from 13 (*m*/*z* = 1093) from the electrospray ionisation spectrum of the reaction mixture across three different reaction mixtures (from two independent experiments) gave an average value of (19 ± 0.7)% conversion, close to the value from the GO/HRP assay (Fig. 5b).

The β4GalT1-mediated conversion of DMPC/2 liposomes (15 to 20%) is significantly lower than the 60% and 59% conversion obtained in solution for 6 and 4, respectively. The steric constraint introduced by the bilayer on access to the enzyme active site is likely to be a contributing factor, an effect that does not seem to be alleviated by the TEG linker in 1. Another factor is the reduction in substrate availability, with up to half of 2 facing the interior of the liposome and being inaccessible to externally added enzyme (depending on flip-flop rates). Conversion is however comparable to the 14% galactosylation reported when applying bovine β4GalT1 and UDP-Gal to a synthetic GlcNAc-capped fluorescent lipid in DMPC liposomes for 24 h.^62^

### Multienzyme transformation of 2 in DMPC liposomes

Given the successful use of β4GalT1 to transform DMPC/2 liposomes and the failure to directly condense 3′SL with lipid 1, it was hoped that the use of multiple glycosyltransferases would provide more complex glycolipids. Applying multienzyme synthetic sequences to synthetic sugars has been reported to give difficult-to-access bioactive oligosaccharides.^31^ Such complex oligosaccharides on the surface of drug-loaded liposomes may produce highly specific targeting of particular cell types.

Combinations of three enzymes with glycosyltransferase activity were tested, namely β4GalT1, TcTS and α-1,3-fucosyltransferase (α1,3-FucT). Combinations of these enzymes might provide three new oligosaccharide motifs: Neu5Ac(α2–3)LacNAc, Lewis X (Le^x^) and sialyl Lewis X (sLe^x^). These motifs have applications in drug delivery systems^2,32,63^ and/or in vaccines.^64^

The methodology was first validated using soluble GlcNAc-PNP. The *p*-nitrophenyl (PNP) chromophore permits reaction monitoring by HPLC, which provides quantitative timecourse data. The conversion of GlcNAc-PNP to Neu5Ac(α2–3)LacNAc PNP with β4GalT1/UDP-Gal and TcTS/3′-SL using a “one-pot” procedure has already been reported to give 70% of the trisaccharide, with 25% of LacNAc-PNP, within an hour.^32^ This methodology was extended to the synthesis of Le^x^-PNP using β4GalT1 and α1,3-FucT enzymes in both sequential and “one-pot” approaches. If successful, a “one-pot” method could decrease total synthesis time and associated costs; it also takes full advantage of the stereo- and regioselectivity of glycosyltransferases.^65^ HPLC analysis revealed the sequential approach gave 92% conversion after 5 h whereas a one-pot approach gave 75%, indicating that β4GalT1 activity is compromised when mixed with α1,3-FucT and associated reagents. Sialylation of Le^x^-PNP by TcTS could provide sLe^x^-capped PNP. However, in the natural biosynthetic pathway,^66^ sialylation generally occurs before fucosylation,^65^ so α1,3-FucT addition was made the final step. Following treatment of GlcNAc-PNP with β4GalT1/UDP-Gal and TcTS/3′-SL, the sialyated product mixture was fucosylated with α1,3-FucT/L-Fuc over 16 h (with GDP-fucose recycling, see the ESI† for details). This gave a mixture of sLe^x^-PNP and Le^x^-PNP with (30 ± 1)% and (67 ± 1)% conversion, respectively. These validation studies showed that on non-oxime substrates in solution, these enzyme combinations can provide the three target oligosaccharide motifs.

The lack of a chromophore in 2 prevented the use of HPLC to quantify multienzyme transformations of this substrate. However LC-MS and lectin-medicated liposome aggregation are alternatives that allow qualitative monitoring of the *in situ* enzymatic elaboration of synthetic glycolipids in liposomes.^33,34^ To discriminate between liposomal coatings, WGA, ECL, *Maackia amurensis* lectin II (Mall II) and *Ulex europaeus* agglutinin I (UEA I) lectins were used due to their reported specificity towards terminal GlcNAc, Gal, Neu5Ac and Fuc carbohydrates respectively.^56,67^

**Fig. 6:**
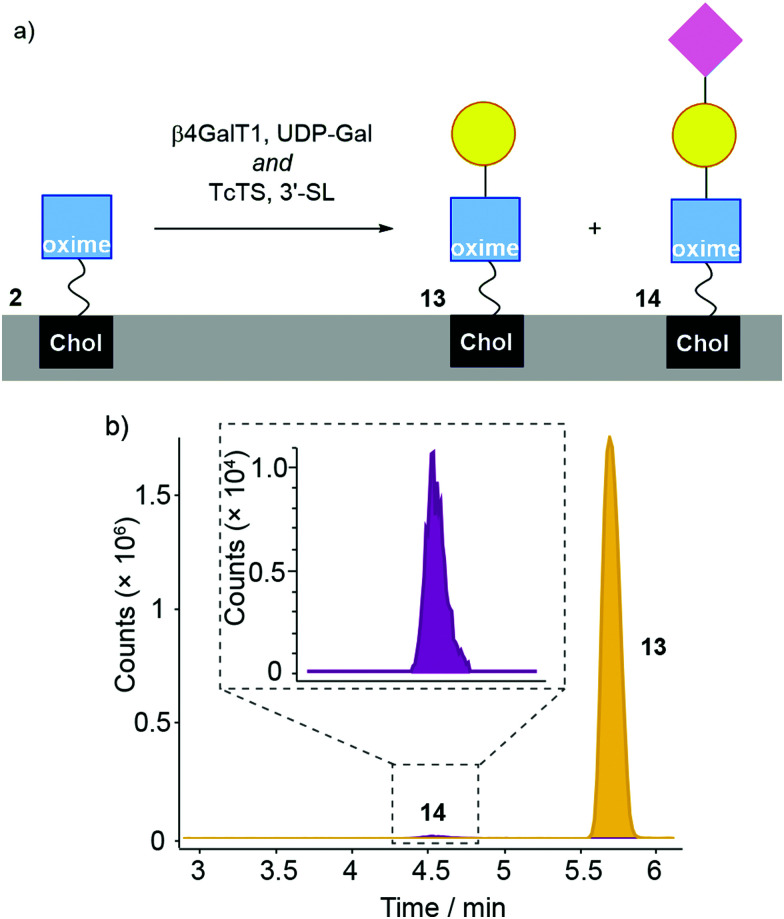
(a) Enzymatic transformation of DMPC/2 liposomes using bovine β4GalT1 and TcTS (with UDP-Gal, MnCl_2_, 3-sialyllactose) affording 13 and 14. (b) LC-MS analysis of DMPC/2 liposomes incubated with β4GalT1/UDP-Gal and TcTS/3-siallyllactose in the same reaction step. For clarity, the peak from 2 is not shown here (see the ESI†).

A “one-pot” combination of β4GalT1 and TcTS has been shown to work on GlcNAc-PNP and GlcNAc-coated liposomes, so was applied to 2 in liposome membranes (Fig. 6). DMPC/2 liposomes (200 μM in MES buffer) were mixed with UDP-Gal, MnCl_2_, 3-sialyllactose, bovine β4GalT1 and TcTS. After overnight incubation, liposomes were analysed by LC-MS, with the data obtained then analysed based on extracted chromatograms relative to 2 ([M + Na]^+^ 931.598), 13 ([M + Na]^+^ 1093.651) and 14 ([M + Na]^+^ 1384.747) masses (Fig. 6b, adduct 2 not shown). All three masses were identified, with product 14 eluting at 4.5 min followed by 13 at 5.7 min and 2 at 6 min. Although these LC-MS data are not quantitative, it is clear that sialylation is relatively poor. In keeping with this low level of sialyation, there was no significant increase in turbidity upon mixing with MAL II. In addition, fluorescence microscopy of MAL II mixed with β4GalT1/TcTS transformed DMPC/2 liposomes (800 nm, labelled with rhodamine DHPE) did not show a significant number of aggregates (see the ESI†).

Given that conversion of GlcNAc-PNP to Neu5Ac(α2–3)LacNAc-PNP was up to 70% when using these two enzymes in solution, these data indicate a strong decrease in conversion at the liposome surface. This is a stronger decrease than similar liposomal studies on non-oxime glycolipids that showed up to 20% conversion,^32^ which may indicate TcTS is sensitive to the oxime link. Further studies on chromophoric LacNAc oximes in solution would be needed to confirm this suggestion.

**Fig. 7:**
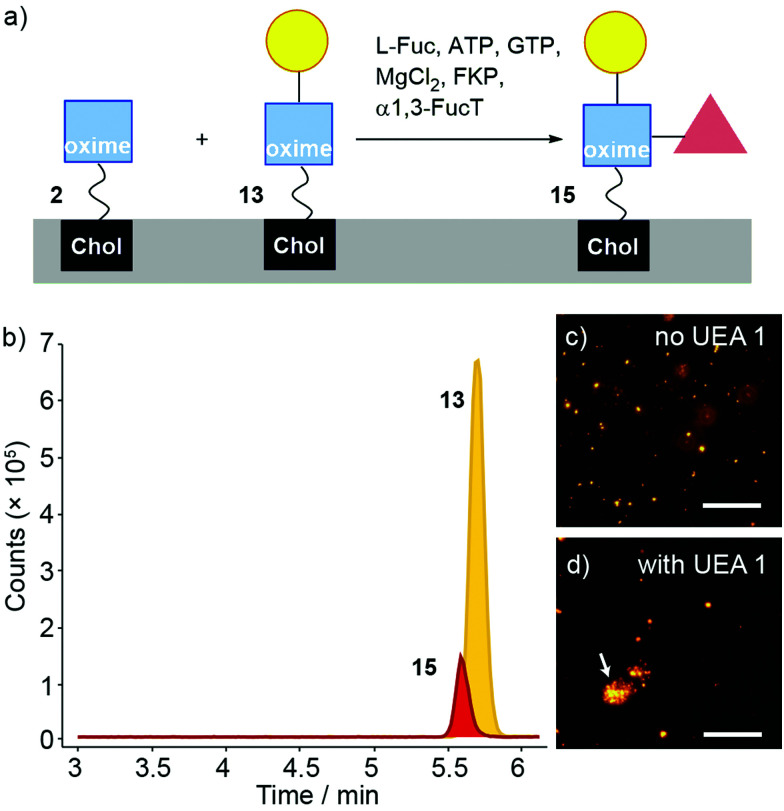
(a) α1,3-FucT/FKP/Fuc mediated transformation of a mixture of 2 and 13 (after β4GalT1, *ca.* 80% and 20% respectively) in DMPC liposomes to afford 15. (b) LC-MS analysis of DMPC/(2 + 13 + 15) liposomes incubated with α1,3-FucT/FKP. A large peak corresponding to 2 was present but is not included in the chart. (c and d) Fluorescence microscopy images of DMPC/(2 + 13) liposomes (800 nm, labelled with rhodamine DHPE) after treatment with α1,3-FucT (c) before and (d) after addition of UEA I. A liposome agglomerate is marked with a white arrow. Scale bar 20 μm.

Fucosylation of 13 instead of sialylation would give Lewis X (Le^x^) coated liposomes (Fig. 7a). The “sequential” enzyme approach was applied to DMPC/2 liposomes. Liposomes were mixed with UDP-Gal, MnCl_2_ and β4GalT1. After overnight incubation, the liposomes were incubated with L-Fuc, ATP, GTP, MgCl_2_, GDP-fucose pyrophosphorylase (FKP) and α1,3-FucT. This suspension was incubated for 6 hours then the enzymatically transformed liposomes analysed by LC-MS. Three oxime masses were identified. The data obtained was then analysed based on extracted chromatograms relative to 2 ([M + Na]^+^ 931.598), 13 ([M + Na]^+^ 1093.651) and 15 masses ([M + H]^+^ 1217.727) (Fig. 7b). The Le^x^-capped lipid 15 eluted at 5.5 min followed by LacNAc-capped 13 at 5.6 min and GlcNAc-capped lipid at 2 at 6 min (not shown). Although addition of the fucose-selective UEA I lectin did not produce significant changes in turbidity and particle size (as monitored by DLS) compared to controls, fluorescence microscopy of UEA I lectin mixed with β4GalT1/α1,3-FucT transformed DMPC/2 liposomes (800 nm, labelled with rhodamine DHPE) showed some small aggregates (Fig. 7d). The low proportion of 15 compared to 13 indicates that surface fucosylation of oxime 13 is much less efficient than for the PNP analogues in solution, which were converted rapidly and in high yield (see the ESI†).

**Fig. 8:**
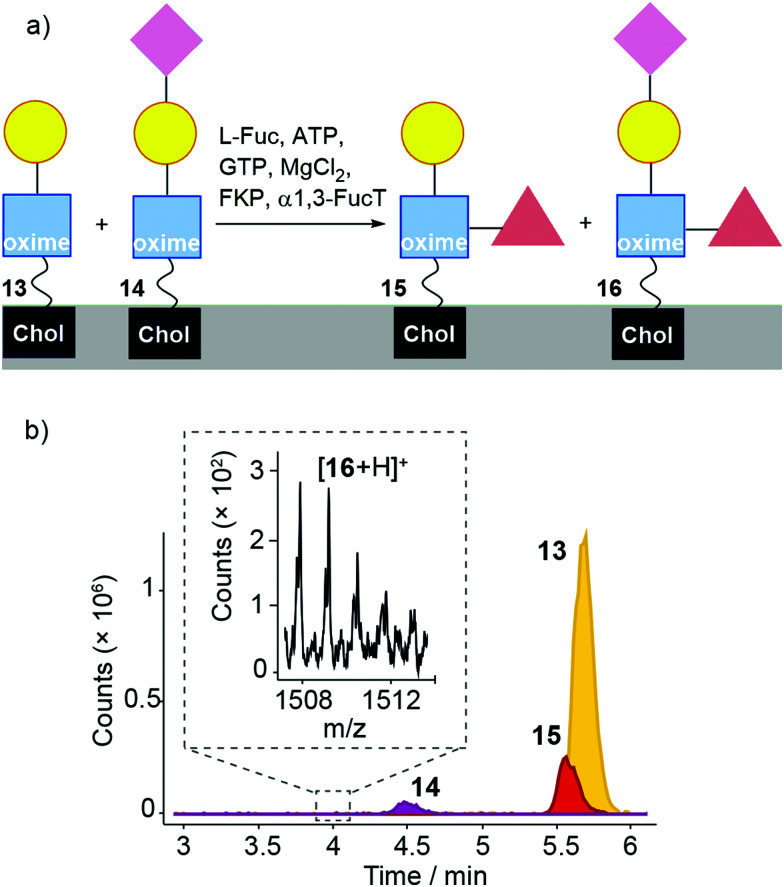
(a) Enzymatic transformation of a mixture of 2, 13 and 14 (formed by the action of β4GalT1 and TcTS on 2) using α1,3-FucT/FKP/Fuc, affording 15 and 16. (b). LC-MS analysis of DMPC/(2 + 13 + 14) liposomes (from the ‘one-pot’ β4GalT/TcTS reaction) incubated with α1,3-FucT/FKP. For clarity, the peak from 2 is not shown here (see the ESI†). Inset: MS data for 16 is found in a small peak eluting at 4 min. Expected for [16 + H]^+^ (*m*/*z*): 1508.8 (100.0%).

Sialylation and fucosylation of DMPC/13 liposomes would produce liposomes coated with sLe^x^ (Fig. 8). DMPC/2 liposomes were first mixed with UDP-Gal, MnCl_2_, 3-sialyllactose, β4GalT1 and TcTS. After overnight incubation, the suspension was mixed with L-Fuc, ATP, GTP, MgCl_2_, FKP enzyme and α1,3-FucT (see the ESI†) and incubated for 6 hours. LC-MS data was obtained from these samples, which was analysed based on extracted chromatograms relative to the masses for 2 ([M + Na]^+^ 931.598), 13 ([M + Na]^+^ 1093.651), 14 ([M + Na]^+^ 1384.747), 15 ([M + H]^+^ 1217.727) and 16 ([M + H]^+^ 1508.822). All these masses were identified in the sample, with 14 eluting at 4.5 min, sLe^x^-capped glycolipid 16 eluting at 4.0 min, Le^x^-capped glycolipid 15 eluting at 5.5 min, 13 at 5.6 min and 2 at 6 min. However, these chromatograms show that conversion to the sialylated products was low, with a small amount of 14 and very little sLe^x^-capped glycolipid 16 present.^68^ No significant agglutination was induced by adding UEA I lectin to the liposomal suspensions (see ESI†).

Taken together, these liposomal studies show that galactosylation by β4GalT1 of a GlcNAc-oxime at a bilayer surface (*ca.* 20% conversion) is effective, with subsequent fucosylation by α1,3-FucT/FKP also relatively effective. However, TcTS-catalysed addition of Neu5Ac to either Le^x^-lipid or LacNAc-lipid needs improvement, which might be achieved through the use of a different enzyme that is less affected by the presence of the bilayer. Nonetheless, the production of liposomes displaying LacNAc or Le^X^ in two or three steps respectively from 1 shows the potential of this methodology.

**Fig. 9:**
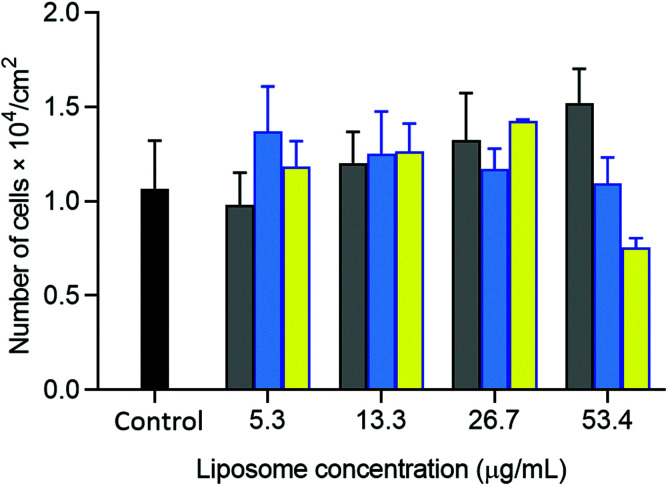
Viability of HepG2 cells (2.5 × 10^4^ cells per cm^2^) incubated with DMPC (gray bars), DMPC/2 (blue bars) and DMPC/(2 + 13) liposomes (yellow bars) at 5.3, 13.3, 26.7, 53.4 μg mL^−1^. Cells incubated in the absence of liposomes used as the control (black bar). Results correspond to the mean ± SD (*n* = 3). Statistical analysis by the non-paired Student's *t* test revealed no significant differences between the control and samples.

The potential of oxime-bearing liposomes as drug delivery vehicles was supported by cell toxicity and drug encapsulation studies. Liposomes displaying these synthetic glycolipids produced little cell toxicity, with initial data showing no significant difference in the viability of HepG2 cells mixed with 26 mg L^−1^ of either DMPC/2 or β4GalT1 transformed DMPC/2 liposomes (DMPC/(2 + 13) liposomes, Fig. 9).

A slight adjustment to the formulation allowed the anti-cancer drug doxorubicin to be retained in the lumen of GlcNAc-coated liposomes using a modification of established procedures.^42^ Cholesterol addition to the liposome membrane allowed retention of the drug over a period of several days, with a DMPC/cholesterol/2 (49 : 41 : 10) composition compatible with the active loading of doxorubicin; 40% of the drug was retained in these GlcNAc-coated liposomes after incubation in buffer for 100 h (see the ESI†).

## Conclusions

Despite a lack of literature reports on the enzymatic elaboration of sugar oximes, a chemoenzymatic approach has been applied to the synthesis of LacNAc-, LacNAc-Neu5Ac-, LeX-, and sLe^x^-capped oxime-linked glycolipids, providing multivalent displays of these saccharides on liposomes.^69^

The well-studied condensation of reducing sugars with *N*-alkoxyamines was used to form the initial glycoconjugates. As anticipated, simple sugars were amenable to condensation, with bivalent *N*-alkoxyamine 3 shown to condense with several simple reducing sugars, whereas a more complex saccharide fragmented.^6,70^ The oximes were a mixture of cyclic and acyclic glycoconjugates, but the transformation of GlcNAc conjugate 6 using β4GalT1/UDP-Gal still proceeded well if extended reaction times were used, with conversions up to 60% after 6 d. Conversion by the β4GalT1/UDP-Gal mixture became faster if the sample of 6 was enriched in the β-anomer, suggesting this isomer is the best substrate for β4GalT1. It is also suggested that extended reaction times allow the β-anomer of 6 to be replenished by isomerisation of the other components. Overall, these observations indicate that the higher hydrolytic stability of the oxime link comes at the expense of lower reactivity with enzymes.

Similarly, enzymatic transformation of GlcNAc lipid conjugate 2 embedded in liposomes by β4GalT1/UDP-Gal also proceeded. The extent of galactosylation was quantified using a GO/HRP coupled assay and estimated from LC-MS data. The measured 15 to 20% conversion after 24 h is lower than the extent of galactosylation of the GlcNAc oxime analogue 6 in solution (50–60%). The major contribution to this decrease is suggested to be the influence of the bilayer rather than the effect of the oxime linkage, since a reduction in conversion to *ca.* 14% has been observed for β4GalT1/UDP-Gal acting on a non-oxime liposome-embedded GlcNAc lipid.^34^

Liposomes doped with GlcNAc-capped adduct 2 could be further transformed using combinations of β4GalT1 with other enzymes, specifically TcTS and α1,3-FucT. The proportions of the oligosaccharide products in liposomes could not be quantified due to the absence of a chromophore, but LC-MS indicated that an *in situ* combination of β4GalT1/α1,3-FucT afforded significant amounts of Le^x^ capped lipid 15 on the liposome surfaces. However, *in situ* sialylation by TcTS/sialyllactose was challenging, with the sLe^X^ capped lipid 16 only detected at very low levels. Sugiarto *et al.* used ‘one-pot’ enzymatic mixtures that included a viral α2–3-sialyltransferase (vST3Gal-I) to synthesise sLe^x^.^65^ This enzyme, unlike TcTS, can tolerate fucosylated substrates and may give better yields of sLe^X^ on the liposome surfaces.

Since adducts 11 and 13 could be accessed directly by *N*-alkoxyamine condensation with LacNAc, this implies that reordering of the synthetic sequence may make more efficient use of enzymatic catalysis, albeit with some loss of the synthetic flexibility that comes with *in situ* modification. Glycosyltransferases in solution could increase the complexity of a core reducing sugar, up to the point where the resulting oligosaccharide is still able to form an oxime without fragmentation.^6,70^ Then condensation of this oligosaccharide with lipid 1 would give an oxime glycolipid that can be inserted into liposome membranes. Further enzymatic steps, for example to attach sensitive sugars like sialic acid,^70^ could be performed *in situ* at the liposome surface. Indeed the modular nature of oxime formation means each component can be simply altered without requiring extensive synthetic redesign. One key improvement would be to add a chromophore that would aid oxime purification by HPLC and allow the continuous monitoring of enzymatic transformations. This in turn would allow the effect of the bilayer on enzyme activity to be quantified more easily, using comparative HPLC assays on oxime glycolipids in liposomes and water-soluble oxime analogues.

Oligosaccharides accessible only through chemical synthesis could be integrated into the oxime synthesis pathway to give additional functionality, for example oligosaccharides labelled with spectroscopic probes like fluorine.^71^ In addition, given that oxime formation described herein is carried out on relatively small scales (typically <100 mg due to the use of HPLC purification), chemical synthesis may provide large quantities of natural/unnatural reducing sugars before smaller scale bioconjugation reactions.

This simple methodology should also be able to label other biomaterial surfaces with oligosaccharides in the same way; oxime formation followed by *in situ* enzymatic transformation. However, given that enzymatic glycosylations appear to be more efficient in solution, the yield of target oligosaccharides might be improved by using an alternative sequence. Native reducing sugars could be condensed with chromophoric *N*-alkoxyamines, which also bear a click chemistry “tag” to give water-soluble intermediates. “One-pot” multienzyme elaboration of the oximes in solution would be followed by high-yielding ligation with a surface functionalised with reactive groups, to give the functionalised biomaterial. Further investigations in this area are continuing. Since little cell toxicity was observed with oxime-doped liposomes, we hope this chemoenzymatic methodology may lead to the development of new carbohydrate antigens, new targeted drug delivery vehicles and novel biomedical wound-healing materials.

## Author contributions

J. Silva: methodology, validation, formal analysis, investigation, data curation, writing-reviewing and editing, visualization. R. Spiess and A. Marchesi: methodology, resources. J. E. Gough and S. Flitsch: methodology, resources, writing-reviewing and editing, supervision. S. J. Webb: conceptualization, methodology, writing-original draft preparation, writing-reviewing and editing, supervision, project administration, funding acquisition.

## Conflicts of interest

There are no conflicts to declare.

## Supplementary Material

TB-010-D2TB00714B-s001
