# Supplementary material for: Enzymatic elaboration of oxime-linked glycoconjugates in solution and on liposomes
Source: J Mater Chem B. 2022 Jun 14;10(26):5016–27. doi: 10.1039/d2tb00714b (PMC9258907; doi:10.1039/d2tb00714b)
Supplement: TB-010-D2TB00714B-s001 [file TB-010-D2TB00714B-s001.pdf]

Electronic Supplementary Material (ESI) for Journal of Materials Chemistry B.
This journal is © The Royal Society of Chemistry 2022

Supplementary Information

Enzymatic elaboration of oxime-linked glycoconjugates in solution and on liposomes

Joana Silva,[1,2] Reynard Spiess,[2] Andrea Marchesi,[1,2] Sabine L. Flitsch,[1,2] Julie E. Gough,[3] and Simon J. Webb*,[1,2]

1. *Department of Chemistry, University of Manchester, Oxford Road, Manchester M13 9PL, United Kingdom. E-mail: S.Webb@manchester.ac.uk; Tel: +44(0)-161-306-4524.*
2. *Manchester Institute of Biotechnology, University of Manchester, 131 Princess St, Manchester M1 7DN, United Kingdom.*
3. *Department of Materials and Henry Royce Institute, The University of Manchester, Manchester M13 9PL, United Kingdom.*

Contents

Abbreviations 4

1. Materials and instrumentation 6

1.1. Materials 6
1.2. Instrumentation 6

2. Synthesis of *N*-alkoxyamines **1** and **3** 8

2.1. Synthesis of 13-(2'-(t-butoxycarbonylaminooxy)acetamido)-4,7,10-trioxatridecyl 2"-(t-butoxycarbonylaminooxy)acetamide (**S3**) 8
2.2. Synthesis of 13-(2'-(aminooxy)acetamido)-4,7,10-trioxatridecyl 2"-(aminooxy)acetamide, bistrifluoroacetate salt (**3**) 9
2.3. Synthesis of cholesteryl (13-amino-4,7,10-trioxatridecyl)carbamate (**S2**) 9
2.4. Synthesis of cholesteryl (2,2-dimethyl-4,8-dioxo-3,6,13,16,19-pentaoxa-5,9-diazadocosan-22-yl)carbamate (**S4**) 10
2.5. Synthesis of cholesteryl (1-(aminooxy)-2-oxo-7,10,13-trioxa-3-azahexadecan-16-yl)carbamate, lipid **1** 11

3. Synthesis of glycoconjugates with *N*-ethoxyammonium hydrochloride 12

3.1. Formation of GlcNAc/*N*-ethoxyamine adduct **6** (mixture of *E*/*Z*/α/β-anomers) 12
3.2. Isomer interconversion from **6** enriched in *E*-isomer 12
3.3. Enzymatic galactosylation of **6** 14
3.4. Enzymatic galactosylation of **6** enriched in *E*-isomer 14

4. Synthesis of glycoconjugates of tether **3** 16

4.1. Dimeric adducts with tether **3** 16
*Formation of **4**, adduct of **3** with GlcNAc* *16*
4.2. Monomeric adducts with tether **3** 18
*General experimental procedure for adducts **7**–**10*** *18*
*Formation of monomeric adduct **7**, adduct of **3** with Gal* *18*
*Formation of monomeric adduct **8**, adduct of **3** with Glc* *18*
*Formation of monomeric adduct **9**, adduct of **3** with Man* *19*
*Formation of monomeric adduct **10**, adduct of **3** with Lac* *19*
4.3. Mixtures of monomeric and dimeric adducts with tether **3** 20
*General experimental procedure for adducts **S5**-**S22*** *20*
*Formation of **S5** and **4**, adducts of **3** with GlcNAc* *20*
*Formation of **S6** and **S12**, adducts of **3** with L-Fuc* *21*
*Formation of **S7** and **S13**, adducts of **3** with GlcN* *21*
*Formation of **S8** and **S14**, adducts of **3** with Glc-6-P* *22*
*Formation of **S9** and **S15**, adducts of **3** with 2-deoxyGlc* *22*
*Formation of **S10** and **S16**, adducts of **3** with LacNAc* *23*
*Formation of **S11** and **S17**, adducts of **3** with 3'-SL* *24*

5. Synthesis of glycoconjugates of lipid **1** 25

5.1. Synthesis of glycolipid **2**, adduct of lipid **1** with GlcNAc 25
5.2. Synthesis of glycolipid **13**, adduct of lipid **1** with LacNAc 26
5.3. HRMS data 27

6. NMR spectra for intermediate **S2** and new compounds **S3**, **3**, **S4** and **1** 28

^{1}H-NMR spectrum of **S3** in $CDCl_3$ at 298 K. 28

^{13}C-NMR spectrum of **S3** in $CDCl_3$ at 298 K. 28
^{1}H-NMR spectrum of **3** in D_2O at 298 K. 29
^{13}C-NMR spectrum of **3** in D_2O at 298 K. 29
^{1}H-NMR spectrum of **S2** in $CDCl_3$ at 298 K. 30
^{13}C-NMR spectrum of **S2** in $CDCl_3$ at 298 K. 30
^{1}H-NMR spectrum of **S4** in CD_3OD at 298 K. 31
^{13}C-NMR spectrum of **S4** in $CDCl_3$ at 298 K. 31
^{1}H-NMR spectrum of **1** in CD_3OD at 298 K. 32
^{13}C-NMR spectrum of **1** in CD_3OD at 298 K. 32

7. Enzymatic transformation of GlcNAc-PNP **S18** 33

Enzymatic transformation of **S18** using combinations of β4GalT1, TcTS and α1,3-FucT 33
Overview *33*
Sequential and “one-pot” formation of LeX-PNP 35
Procedures for enzymatic transformation of PNP derivatives 38
Enzymatic transformation using β4GalT1 enzyme *38*
Enzymatic transformation using TcTS enzyme *38*
Enzymatic transformation using α1,3-FucT enzyme *39*
Enzymatic transformation using β4GalT1/TcTS enzymes *39*
Enzymatic transformation using β4GalT1/α1,3-FucT enzymes *39*

8. Liposomal studies 40

Liposome preparation 40
Procedure for the enzymatic transformation of liposomes 41
Liposome zeta potential and DLS 41
Agglutination assay using lectins 42
Agglutination assay using UV analysis by a plate reader *42*
Agglutination assay using DLS *42*
Agglutination assay using Fluorescence Microscopy *42*
Measurement of galactosylation in enzymatically transformed liposomes 44
Quantification by galactose oxidase assay *44*
Estimation by LC-MS *46*
Quantification of available *N*-alkoxyamino groups on liposome surfaces 47
Solubility of glycolipid **2** in buffer 49
Monitoring of multienzyme transformation of **2** embedded in liposomes by LC-MS. 50
Studies of liposome stability over time 50
Procedure for the active encapsulation of doxorubicin in liposomes 52
Efficiency of doxorubicin encapsulation in liposomes 54
Release of doxorubicin from loaded liposomes 56

9. Cell toxicity assays 58

Procedure for culture of human hepatocellular carcinoma cell line (HepG2) 59
Procedure for measuring cell viability using Alamar blue 59

10. References 60

Abbreviations

**ABTS**	2,2-Azino-bis(3-ethylbenzothiazoline-6-sulfonic acid) diammonium salt
**ATP**	Adenosine 5'-triphosphate
**Boc**	*tert*-Butoxycarbonyl
**BSA**	Bovine serum albumin
**chol**	Cholesterol
**COSY**	Correlation spectroscopy
**d**	doublet
**DAPI**	4',6-Diamidino-2-phenylindole
**DCM**	Dichloromethane
**DHPE**	1,2-Dihexadecanoyl-*sn*-glycero-3-phosphoethanolamine
**DIPEA**	*N*,*N*-Diisopropylethylamine
**DLS**	Dynamic light scattering
**DMEM**	Dulbecco's modified eagle media
**DMPC**	1,2-Dimyristoyl-*sn*-glycero-3-phosphocholine
**DOPC**	1,2-Dioleoyl-*sn*-glycero-3-phosphocholine
**DOX**	Doxorubicin
**DPPC**	1,2-Dipalmitoyl-*sn*-glycero-3-phosphocholine
**EA**	Elemental analysis
**ECL**	*Erythrina Cristagalli* lectin
**EMEM**	Eagle's Minimum Essential Medium
**eq.**	equivalent
**ES**	Electrospray ionisation
**FBS**	Foetal bovine serum
**FITC**	Fluorescein isothiocyanate
**FKP**	L-fucokinase/GDP-Fuc pyrophosphorylase
**FSC**	Forward scatter
**FT-IR**	Fourier transform infra-red spectrometry
**Fuc**	Fucose
**FucT**	Fucosyltransferase
**GalT**	Galactosyltransferase
**GDP**	Guanosine-5'-Diphosphate
**GlcT**	Glucosyltransferase
**GOase**	Galactose oxidase
**GPC**	Gel permeation chromatography
**GTP**	Guanosine 5'-triphosphate
**GTs**	Glycosyltransferases
**HCC**	Hepatocellular carcinoma
**HEPES**	4-(2-Hydroxymethyl)-1-piperazineethanesulfonic acid
**HepG2**	Human hepatocyte carcinoma cells
**HPLC**	High performance liquid chromatography
**HRMS**	High resolution mass spectrometry
**HRP**	Horseradish peroxidase

**LC-MS**	Liquid chromatography-mass spectrometry
**l_d**	Liquid disordered
**Le^x**	Lewis X
**LUV**	Large unilamellar liposome
**m**	multiplet
m/z	Mass divided by charge number of ions
**MAL II**	*Maackia Amurensis* lectin II
**MEM**	Minimum essential media
**MES**	2-(*N*-morpholino)ethanesulfonic acid
**mL**	millilitre
**MS**	Mass spectrometry
n	Number of replicates
**nm**	nanometre
**NMR**	Nuclear magnetic resonance
**PBS**	Phosphate buffered saline
**PdI**	Polydispersity Index
**PEG**	Polyethylene glycol
**PNP**	*p*-Nitrophenyl
**ppm**	parts per million
**q**	quartet
**RFU**	Relative fluorescence units
**RT**	Room temperature
**s**	singlet
**SA**	Sialic acid
**SialT**	Sialyltransferase
**3’-SL**	3’-sialyllactose
**sLe^x**	Sialyl Lewis X
**SSC**	Side scatter
**ssNMR**	Solid state nuclear magnetic resonance spectroscopy
**TcTS**	*Trypanosoma cruzi* trans-sialidase
**TFA**	Trifluoroacetic acid
**THF**	Tetrahydrofuran
**TLC**	Thin layer chromatography
**T_m**	Transition temperature
**TNBS**	2,4,6-Trinitrobenzenesulphonic acid
**UDP**	Uridine diphosphate
**UEA I**	*Ulex europaeus* agglutinin I
**UV**	Ultraviolet
**WGA**	Wheat germ agglutinin
**β4GalT1**	β(1,4)-galactosyltransferase

1. Materials and instrumentation

1.1. Materials

Reagents were purchased from Sigma-Aldrich Co. Ltd., Dorset, UK with some exceptions. DMPC and DOPC were purchased from Avanti Polar Lipids Inc., Alabama, USA. Lissamine™ rhodamine DHPE and BioDesignDialysis Tubing® were purchased from ThermoFisher Scientific Inc., USA. All lectins were purchased from Vector Labs. PD-10 gel permeation chromatography (GPC) columns were purchased from GE Healthcare. Vivaspin®500 was obtained from Sartorius Stedin Biotech.

β4GalT1 enzyme (bovine, P08037) was expressed in-house by Dr Andrea Marchesi (University of Manchester, UK) in *E. coli*. TcTS enzyme was expressed in-house by Dr Sara Kaloo (University of Manchester) in *E. coli*. LgtB (bacterial, Q51116, expressed in *E. coli*), β4GalT1 (human, P15291, expressed in *E. coli* Shuffle) and α1,3-FucT (O30511, expressed in *E. coli*) were kindly provided by Prozomix Ltd (Haltwhistle NE49 9HA, UK).

PBS, cell culture media (EMEM), L-glutamine, penicillin, streptomycin, accutase, foetal bovine serum (FBS), bovine serum albumin (BSA), well plates, tissue culture flasks, paraformaldehyde and DAPI were all obtained from Sigma-Aldrich Co. Ltd., Dorset, UK. Alexa Fluor 488 Phalloidin was obtained from Thermo Fisher Scientific Inc., USA. HepG2 cells were obtained from ECACC (*via* Sigma-Aldrich Co. UK).

1.2. Instrumentation

NMR measurements were made on either a Bruker Ultrashield 400 or a Bruker 500 MHz spectrometer at RT (295 K) in a suitable deuterated solvent (typically $CDCl_3$, D_2O or CD_3OD). Chemical shifts (δ) were measured in parts per million (ppm) and coupling constants (*J*) in Hertz (Hz). ^{1}H- and ^{13}C-NMR spectroscopy are referenced relative to the residual solvent peaks. Multiplicities are indicated as singlet (s) doublet (d), doublet of doublets (dd), triplet (t), quartet (q), and multiplet (m). Spectra assignments were made by 2D COSY, HMQC, HSQC, DEPT-90 and DEPT-135 measurements to aid peak identification. NMR data were processed and analysed using MestreNova.

Electrospray mass spectrometry was performed on an Agilent 6560 IMS Qtof MS with an Agilent 1290 Infinity 2 series LC. High resolution mass spectrometry was performed on a Water Q-TOF micro with an ES+/- ion source. For GC-MS experiments an Agilent 1200 series 6510 Q-TOF LC-MS was used. Reversed-phase HPLC purification was performed either on an Agilent 1100 series system, LC system with either a G1315B diode-array detector or a G1365B multi-wavelength detector. LC−MS measurements were made using an Agilent 1100 LC/MSD Ion Trap System with an Electrospray Source and positive ionisation. The following columns were used: Agilent Zorbax Eclipse XDB-C_8 (150 × 4.4 mm, 5 µm; used for the quantification of galactosylation of **2** by LC-MS) fitted with a guard column, Macherey-Nagel Nucleosil C_{18} (150 × 4.6 mm, 5 µm, used for the transformation of GlcNAc-PNP) fitted with a guard column, Agilent Eclipse XDB-C_8 (250 × 9.4 mm, 5 µm; used to purify **2** and **13**) and Agilent Eclipse XDB-C_{18} (250 × 9.4 mm, 5 µm; used to purify other compounds).

FTIR spectra were recorded on a Bruker Alpha-P instrument and analysed using OPUS 6.5 software between 4000 and 500 cm^{-1}. An ATR platinum diamond detector was used. The acquisition was over 64 scans at a resolution of 2 cm^{-1}. Absorbance and fluorescence spectra were measured on an Infinite® 200 PRO NanoQuant plate reader from TECAN and a CLARIOstar plate reader. UV-visible spectroscopy

was measured using a Jasco V-660 spectrophotometer at 400 nm/min in a 200-800 nm range. Elemental analysis was performed on a Thermo Scientific FLASH 2000 series CHNS/O analyser. DLS were performed on a Malvern Zetasizer Nano ZSP 633-nm laser at 25 °C. The ζ potential was measured on a Malvern Zetasizer Nano ZSP at 25 °C using the diffusion barrier method.[1]

Fluorescence images were acquired using a Zeiss Axio Imager A1 fluorescence microscope with a Canon Powershot G6 digital camera attached.

2. Synthesis of *N*-alkoxyamines 1 and 3

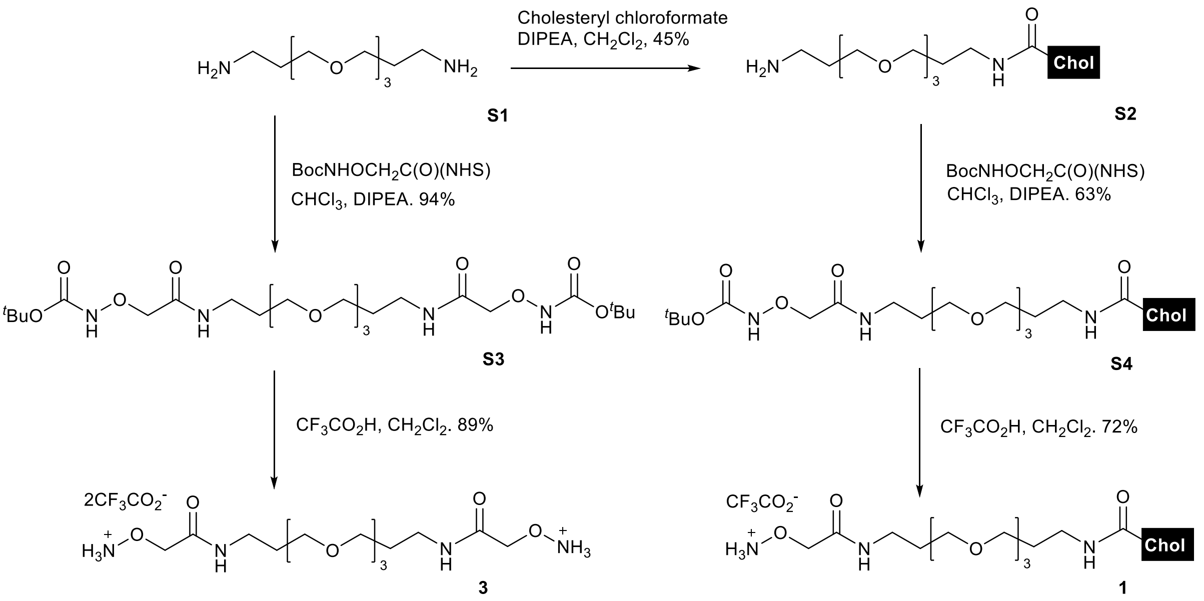

Scheme S1: Synthesis of lipid **1** and tether **3**

To anchor these synthetic oxime-glycolipids to the liposomal membrane, a cholesteryl anchor was selected.[2a] A reactive *N*-alkoxyamine terminus was linked to the cholesteryl unit through a triethyleneglycol spacer, which was hoped to facilitate access of enzymes and lectins to the ligated sugars when the lipid is embedded in a liposomal membrane. The trifluoroacetate salt of *N*-alkoxyamine lipid **1**, which combines these features, was synthesised with 20% overall yield in three steps from commercially available reagents (Scheme S1).

Based on a methodology described by Thakar *et al.*,[2b] the tether **3** was synthesised over two reaction steps using similar synthetic methodology.

2.1. Synthesis of 13-(2'-(t-butoxycarbonylaminooxy)acetamido)-4,7,10-trioxatridecyl 2"-(t-butoxycarbonylaminooxy)acetamide (S3)

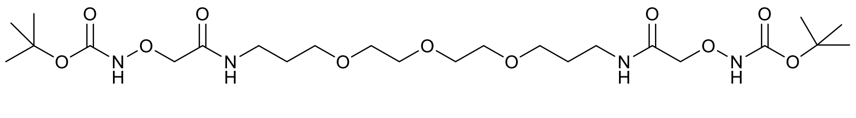

4,7,10-Trioxa–1,13–tridecanediamine (**S1**, 0.22 mmol, 48,2 µL) was dissolved in a mixture of chloroform ($CHCl_3$, 10 mL) and *N*,*N*-diisopropylethylamine (DIPEA, 0.44 mmol, 78 µL), before adding *N*-Boc-aminooxyacetic acid *N*-hydroxysuccinimide ester (0.52 mmol, 149.90 mg) to the solution. After 4 h of stirring at RT, the crude was dissolved into the organic layer ($CHCl_3$) and further washed 3 times using fresh Milli-Q water. The solution was then dried using magnesium sulphate followed by chloroform removal under reduced pressure to afford the pure product **S3** as a yellow oil. Yield: 117 mg, 94%.

^{1}H-NMR (400 MHz, $CDCl_3$): δ_H 8.18 (s, 2H, 2 × CONHO), 7.88 (s, 2H, 2 × CONH), 4.32 (s, 4H, 2 × OCH_2CO), 3.68-3.50 (m, 12H, $(CH_2OCH_2)_3$), 3.40 (q, 4H, *J* = 6.1 Hz, 2 × $NHCH_2$), 1.81 (p, 4H, *J* = 6.1 Hz, 2 × CH_2), 1.47 (s, 18H, 6 × CH_3) ppm. **^{13}C-NMR** (100 MHz, $CDCl_3$): δ_C 168.9 (2 × CO), 157.6 (2 × CO), 82.5 (2 × $NHOCH_2$), 75.8 - 69.5 ($(CH_2OCH_2)_3$), 37.0 (2 × $NHCH_2$), 29.0 (2 × CH_2), 28.1 (6 × CH_3) ppm. **HRMS** for $(C_{24}H_{46}N_4O_{11}Na)^+$ expected 589.3055 found 589.3050. **FT-IR** 3292 (N-H stretching), 2977-2871 (C-H stretching), 1723 (C=O bond stretch, ester), 1654 (C=O bond stretch, ketone), (N-H bending), 1478, 1455 (CH_2 bend symmetric and asymmetric, respectively), 1368 (CH_3 bending), 1251 (C-O stretching), 1107 (C-N stretching) and 727 (CH_2 rocking) cm^{-1}. **Anal. Calcd.** calculated for $C_{24}H_{46}N_4O_{11}.CHCl_3$): C, 43.77%; H, 6.91%; N, 8.17%. Found: C, 43.21%; H, 6.86%; N, 7.94%.

2.2. Synthesis of 13-(2'-(aminooxy)acetamido)-4,7,10-trioxatridecyl 2"-(aminooxy)acetamide, bistrifluoroacetate salt (3)

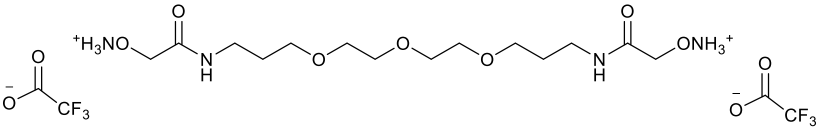

The Boc-protected amines of **S3** were deprotected by dissolving **S3** in a 1:1 (v/v) mixture of trifluoroacetic acid (TFA, 5 mL) and dry dichloromethane (CH_2Cl_2, 5 mL). After stirring for 30 min at RT, the solvent was removed under vacuum and the product was washed five times with diethyl ether (5 mL) to remove residual TFA. Finally, the product was further dried on a high vacuum line to afford compound **3** without further purification. Yield: 111 mg, 89%.

^{1}H-NMR (400 MHz, D_2O): δ_H 4.55 (s, 4H, 2 × OCH_2CO), 3.67-3.48 (m, 12H, $(CH_2OCH_2)_3$), 3.28 (t, 4H, *J* = 6.8 Hz, 2 × $NHCH_2$), 1.76 (p, 4H, *J* = 6.6 Hz, 2 × CH_2) ppm. **^{13}C-NMR** (100 MHz, D_2O): δ_C 168.4 (2 × CO), 71.6 (2 × $NHOCH_2$), 69.4 - 68.2 ($(CH_2OCH_2)_3$), 36.2 (2 × $NHCH_2$), 28.1 (2 × CH_2) ppm. **HRMS** for $(C_{14}H_{31}N_4O_7)^+$ expected 367.2186 found 367.2187. **FT-IR** 3290 (N-H stretching), 2868 (C-H stretching), 1656 (C=O bond stretch), 1555 (N-H bending), 1431 (CH_2 bending), 1132 and 1183 (C-F bond stretch) cm^{-1}. **Anal. Calcd.** calculated for $C_{14}H_{30}N_4O_7(CF_3CO_2H)_2.(H_2O)_2$: C, 34.29%; H, 5.76%; N, 8.89%. Found: C, 36.48; H, 5.41; N, 8.55.

2.3. Synthesis of cholesteryl (13-amino-4,7,10-trioxatridecyl)carbamate (S2)

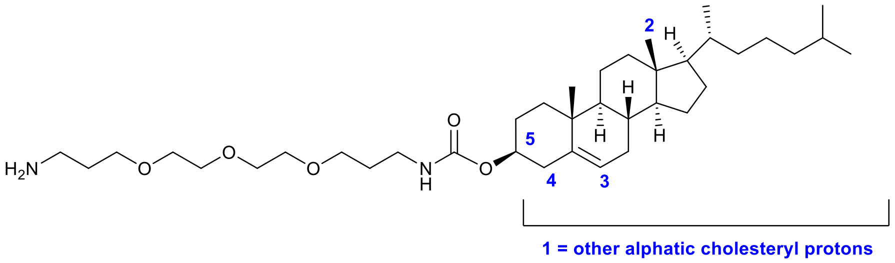

Following methodology described by Leamon *et al.*,[3] 4,7,10-trioxa–1,13–tridecanediamine (**S1**, 5 mmol, 1.1 mL) was mixed with DIPEA (5 mmol, 871 µL) in DCM (10 mL). After 30 min, cholesteryl

chloroformate (1 mmol, 449.12 mg) was added dropwise in the mixture. The solution was stirred overnight at RT. After removing the solvent by reduced pressure, the crude was precipitated by adding MeCN (30 mL) followed by filtration. A white sticky solid was obtained that was subsequently washed several times with distilled H_2O and air-dried. Yield: 276 mg, 45% yield. Spectral data matches that previously reported.[4]

^{1}H-NMR (500 MHz, $CDCl_3$): δ_H 5.35 (d, 1H, J_1=9.0, J_2=11.4 Hz, 3), 5.30 (s, 1H, NH), 4.47 (t, J = 11.3 Hz, 1H, 5), 3.70-3.50 (m, 12H, $(CH_2OCH_2)_3$), 3.27 (d, 2H, J = 7.4 Hz, CH_2NH), 2.89 (t, 2H, J = 6.0 Hz, NH_2CH_2), 2.39-2.18 (m, 2H, 4), 2.05-0.82 (m, 44H, 1, NH_2, 2 × CH_2), 0.67 (s, 3H, 2) ppm. **^{13}C-NMR** (100 MHz, $CDCl_3$): δ_C 156.4 (CO), 139.9 (C), 122.4 (C-3), 74.1 (C-5), 70.5-69.4 ($(CH_2OCH_2)_3$), 56.6 (C), 56.1 (C), 50.0 (C), 42.3 (C), 39.7 (CH_2), 39.6 (CH_2), 39.5 (NH_2CH_2), 38.7 (C-4), 38.6 (CH_2NH), 37.0 (CH_2), 36.5 (C), 36.1 (CH_2), 35.8 (CH), 31.9 (CH_2), 31.8 (C), 31.6 (CH_2 trioxa), 29.5 (CH_2 trioxa), 28.2 (CH_2), 28.0 (CH), 24.2 (CH_2), 23.8 (CH_2), 22.8 (CH_3), 22.5 (CH_3), 21.0 (CH_2), 19.3 (CH_3), 18.7 (CH_3), 11.8 (CH_2) ppm. **HRMS** for $(C_{38}H_{68}O_5N_2K)^+$ expected 671.4765 found 671.4760.

2.4. Synthesis of cholesteryl (2,2-dimethyl-4,8-dioxo-3,6,13,16,19-pentaoxa-5,9-diazadocosan-22-yl)carbamate (S4)

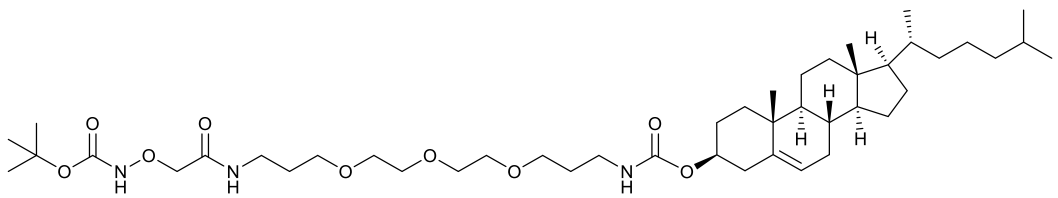

Compound **S2** (0.22 mmol, 139.3 mg) was dissolved in chloroform ($CHCl_3$, 10 mL) followed by the addition of DIPEA (0.22 mmol, 39 µL) and *N*-Boc-aminooxyacetic acid *N*-hydroxysuccinimide ester (0.26 mmol, 75 mg). After stirring the mixture overnight at RT, the solution was concentrated and the crude was dissolved in $CHCl_3$ (20 mL) and washed with deionised water (3 × 20 mL). The organic layer was dried over magnesium sulphate, filtered and evaporated under reduced pressure *via* rotary evaporation. Then, the residue was separated using a silica column (EtOAc/PET 1:1 changed to EtOAc/CH_3OH 20:1) to afford pure product. **TLC** R_f 0.30 (ethyl acetate). Yield: 80.9 mg, 63% yield.

^{1}H-NMR (500 MHz, CD_3OD): δ_H 7.98 (s, 1H, CONHO), 7.87 (s, 1H, CONH), 5.36 (d, 1H, J = 4.9 Hz, 3), 5.16 (s, 1H, NHCOO), 4.47 (t, J = 11.2 Hz, 1H, **5**), 4.31 (s, 2H, OCH_2CO), 3.69-3.50 (m, 12H, $(CH_2OCH_2)_3$), 3.42 (q, 2H, J = 6.2 Hz, CH_2NH), 3.27 (q, 2H, J = 6.3 Hz, $NHCH_2$), 2.39-2.20 (m, 2H, 4), 1.92-0.81 (m, 51H, 1, 2 × CH_2, 6 × CH_3), 0.67 (s, 3H, 2) ppm. **^{13}C-NMR** (100 MHz, CD_3OD): δ_C 168.7 (OCONH), 157.5 (NHCOO), 156.2 (CONH), 139.9 (C), 122.4 (C-3), 82.6 (C Boc group), 75.8 ($NHOCH_2$), 74.1 (C-5), 70.5-69.5 ($(CH_2OCH_2)_3$), 56.6 (C), 56.1 (C), 50.0 (C), 42.3 (C), 39.7 (CH_2), 39.5 (CH_2), 38.8 (C-4), 38.6 (CH_2NH), 37.1 ($NHCH_2$), 37.0 (CH_2), 36.5 (C), 36.1 (CH_2), 35.8 (CH), 31.9 (CH_2), 31.8 (C), 29.5 (CH_2 trioxa), 29.0 (CH_2 trioxa), 28.2 (3 × CH_3 Boc group), 28.1 (CH_2), 28.0 (CH), 24.2 (CH_2), 23.8 (CH_2), 22.8 (CH_3), 22.5 (CH_3), 21.0 (CH_2), 19.3 (CH_3), 18.7 (CH_3), 11.8 (CH_2) ppm. **HRMS** for $(C_{45}H_{79}O_9N_3K)^+$ expected 844.5453 found 844.5448.

2.5. Synthesis of cholesteryl (1-(aminooxy)-2-oxo-7,10,13-trioxa-3-azahexadecan-16-yl)carbamate, lipid 1

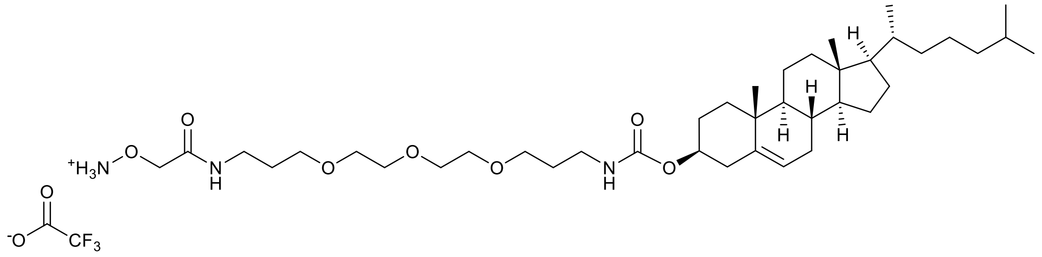

The compound **S4** was deprotected by dissolution in a 1:1 (v/v) mixture of trifluoroacetic acid (TFA, 5 mL) and dry dichloromethane (CH_2Cl_2, 5 mL) and stirring for 30 min at RT. The solvent was then removed *in vacuo* and the product was washed five times with diethyl ether (Et_2O, 5 mL) to remove residual TFA. Finally, the product was further dried for 1 h on a high vacuum line to afford compound **1** without further purification. Yield: 105 mg, 72% yield.

^{1}H-NMR (500 MHz, CD_3OD): δ_H 5.37 (d, 1H, *J* = 4.6 Hz, 3), 4.47 (s, 2H, OCH_2O), 4.35 (m, 1H, 5), 3.67-3.46 (m, 12H, $(CH_2OCH_2)_3$), 3.34 (q, 2H, *J* = 7.0 Hz, CH_2NH), 3.16 (t, 2H, *J* = 6.8 Hz, $NHCH_2$), 2.36-2.23 (m, 2H, 4), 2.08-0.82 (m, 42H, 1, 2 × CH_2), 0.71 (s, 3H, 2) ppm. **^{13}C-NMR** (100 MHz, CD_3OD): δ_C 168.2 (CONH), 157.4 (COO), 139.8 (C), 122.0 (C-3), 74.1 (C-5), 71.7 (NH_2OCH_2), 70.1-69.7 ($(CH_2OCH_2)_3$), 56.7 (C), 56.1 (C), 50.2 (C), 42.0 (C), 39.7 (CH_2), 39.2 (CH_2), 38.3 (C-4), 37.6 (CH_2NH), 36.8 ($NHCH_2$), 36.5 (CH_2), 36.3 (C), 36.2 (CH_2), 35.7 (CH), 31.8 (CH_2), 31.6 (C), 28.9 (CH_2 trioxa), 28.7 (CH_2 trioxa), 27.9 (CH_2), 27.7 (CH), 23.9 (CH_2), 23.5 (CH_2), 21.7 (CH_3), 21.5 (CH_3), 20.7 (CH_2), 20.2 (CH_3), 18.3 (CH_3), 10.9 (C-2) ppm. **HRMS** for $(C_{40}H_{71}O_7N_3K)^+$ expected 744.4929 found 744.4924.

3. Synthesis of glycoconjugates with *N*-ethoxyammonium hydrochloride

3.1. Formation of GlcNAc/*N*-ethoxyamine adduct 6 (mixture of E/Z/α/β-anomers)

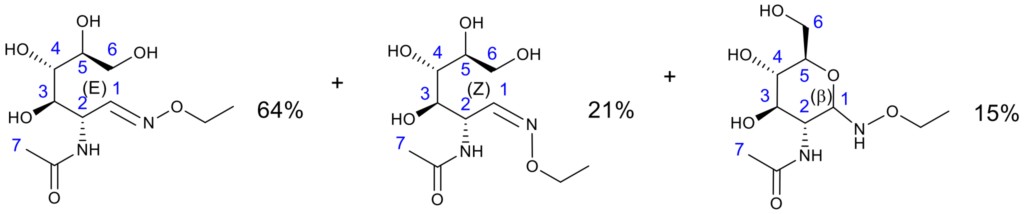

Adduct (*E*)-**6** Adduct (*E*)-**6** Adduct β-**6**

Yield: 120 mg, 46%, 64:21:0:15 mixture of E/Z/α/β-anomers.

The synthesis of the isomers of **6** was accomplished by mixing 1 eq. of *O*-ethylhydroxylamine hydrochloride with 1.5 eq. of GlcNAc in methanol (10 mL). The solution stirred under reflux conditions overnight (65 °C, N_2 atmosphere). The solvent was removed under reduced pressure and the crude mixture was purified by HPLC on a 60 min gradient ranging from 5% to 95% MeCN in water with a flow rate of 1.0 mL/min. The sample was collected and freeze-dried to afford the product as oil.

^{1}H-NMR (400 MHz, CD_3OD): δ_H 7.39 (d, 0.64H, 6.0 Hz, 1E), 6.69 (d, 0.21H, 6.2 Hz, 1Z), 5.10 (t, 0.25H, 5.8 Hz, 2Z), 4.69 (t, 0.66H, 6.4 Hz, 2E), 4.21 (d, 0.15H, *J* = 9.7 Hz, 1β), 4.11 (dd, 0.32H, J_1=7.1, J_2=1.3 Hz, 3Z), 4.05 (q, 2.00H, *J* = 7.1 Hz, CH_2), 4.00 (dd, 0.65H, J_1=6.8, J_2= 1.9 Hz, 3E), 3.84 (dd, 0.21H, J_1=11.9, J_2=1.9 Hz, 6aβ), 3.76 (dd, 0.86H, J_1=10.7, J_2= 2.9 Hz, 6aE), 3.71 (dd, 0.30H, J_1=7.0, J_2=2.6 Hz, 6aZ), 3.68-3.56 (m, 2H, 4, 5), 3.49 (dd, 0.64H, J_1=8.0, J_2=1.9 Hz, 6bE), 3.49 (dd, 0.28H, J_1=7.7, J_2= 2.9 Hz, 6bZ), 3.41 (dd, 0.13H, J_1=8.5, J_2=1.9 Hz, 6bβ), 1.97 (3×s, 3H, 7), 1.20 (3×t, 3H, *J* = 7.0 Hz, CH_3) ppm. **^{13}C-NMR** (100 MHz, CD_3OD): δ_C 171.8 (CO), 149.4 (C-1 E), 147.6 (C-1 Z), 90.0 (C-1 β), 71.5 (C-3 E), 70.6 (CH), 70.0 (C-3 β), 69.2 (C-3 Z), 68.8 (CH_2), 63.3 (C-6), 63.2 (C-2 β), 52.1 (C-2 E), 49.1 (C-2 Z), 47.8 (CH), 21.2 (C-7), 13.4-12.9 (3 × CH_3) ppm. **HRMS** for $(C_{10}H_{20}O_6N_2Na)^+$ expected 287.1214 found 287.1211.

Data matched that reported previously.[5]

3.2. Isomer interconversion from 6 enriched in *E*-isomer

An alternative purification of the reaction mixture of glycoconjugates **6** by HPLC afforded a fraction with a large proportion of open chain (*E*)-oxime (E/Z/α/β, 93:4:0:3). Assuming that open-chain (*E*)-oxime is not a substrate for the β4GalT1 enzyme whereas the β-anomer is, access to this enriched fraction allowed assessment of whether equilibration between isomers allows open-chain oximes to form substrate for this enzyme.

To determine the amount of time required to produce the ring-closed form in significant amounts, the selected fraction (open-chain (*E*)-oxime) was dissolved in buffered D_2O (50 mM MES buffer, pH 7) and monitored by ^{1}H-NMR spectroscopy over time at 37 °C (Figure S1a). After six days, the (*E*)-oxime was still the predominant isomer (61%) in the mixture but the (*Z*)-oxime and cyclic β-anomer were detected in significant amounts. Based on the integration of the respective doublets, the (Z)-oxime

and the cyclic β-anomer proportions were 21% and 18%, respectively. The α-pyranoside isomer was not observed during the course of the experiment (*i.e.* *E*/*Z*/α/β was 61:21:0:18). The 2D COSY spectrum of the mixture before and after 6 days shows the increased intensity of the crosspeak corresponding to the β-anomeric doublet (Figure S1b,c). These observations suggest that the (*E*)-oxime isomer can interconvert into the cyclic β-anomer to re-establish equilibrium.

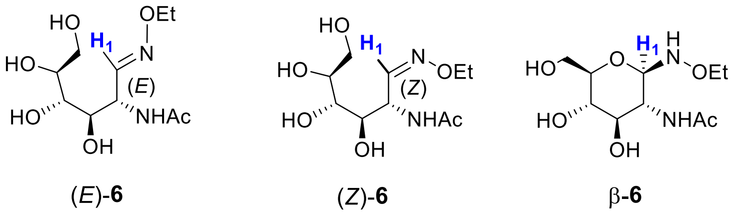

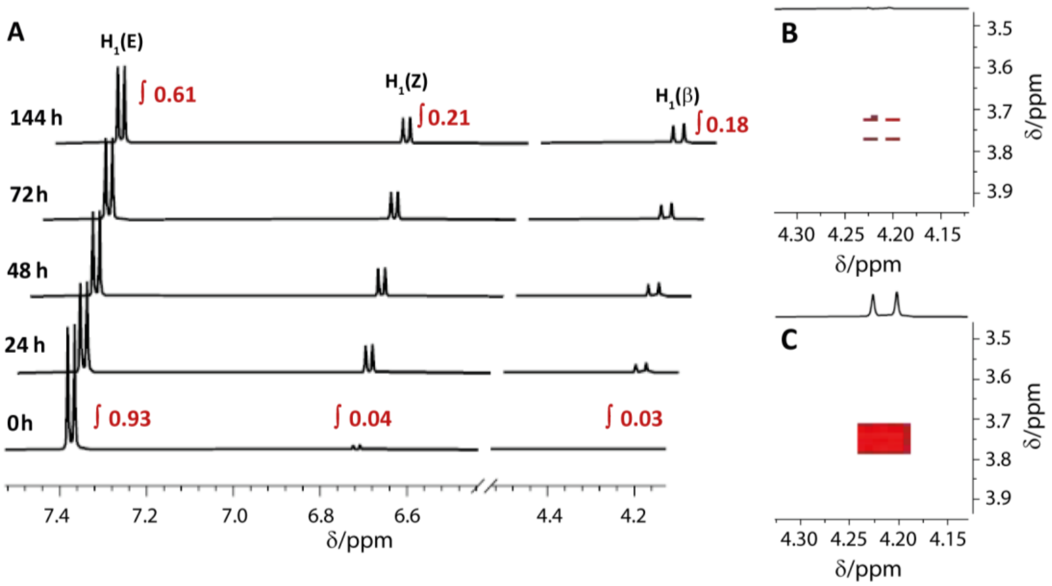

Figure S1. Equilibration of glycoconjugate **6** (fraction predominantly (*E*)-oxime) over time monitored by NMR spectroscopy (buffered D_2O, 295 K, 400 MHz). (A) Interconversion of acyclic/cyclic adducts is achieved over 6 days. (B) 2D COSY NMR spectrum (buffered D_2O, 295 K, 400 MHz) before and (C) after 6 days shows a cross-peak corresponding to the anomeric proton of the β-oxime isomer.

3.3. Enzymatic galactosylation of 6

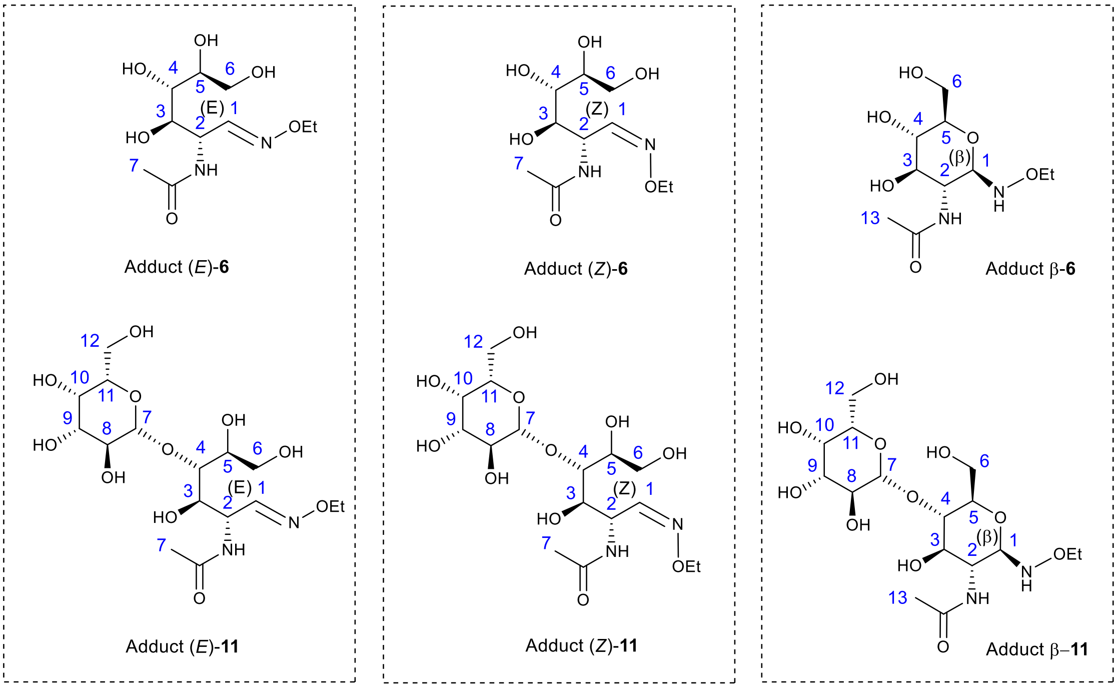

60% + 24% + 16%

Yield: 3 mg, 35% mass recovery. 60:24:0:16 mixture of *E*/*Z*/α/β-anomers, 50% **6** and 50% **11**.

The GlcNAc/*N*-ethoxyamine adduct **6** (0.02 mmol, 4.6 mg) was dissolved in MES buffer (1 mL, 50 mM, pH 7.0) and mixed with UDP-Gal (0.03 mmol, 18.3 mg), $MnCl_2$ (3 µL of a 1.0 M solution in water) and β4GalT1 (17.19 µL of a 0.54 mg/mL solution). The mixture was vortex mixed and incubated overnight at 37 °C. The final product was purified by HPLC on a 40 min gradient ranging from 5% to 100% MeCN in water with a flow rate of 1.0 mL/min. The sample was collected and freeze-dried to afford a 1:1 mixture of **6** and the product **11** (3 mg in total, 35% mass recovery).

^{1}H-NMR (500 MHz, CD_3OD): δ_H 7.39 (d, 0.60H, *J* = 6.0 Hz, 1E), 6.69 (d, 0.24H, *J* = 6.2 Hz, 1Z), 5.10 (t, 0.36H, *J* = 5.5 Hz, 2Z), 4.69 (t, 0.66H, 6.4 Hz, 2E), 4.41 (d, 0.23H, *J* = 7.7 Hz, 7), 4.21 (d, 0.16H, *J* = 9.8 Hz, 1β), 4.13 (dd, 0.25H, J_1=7.1, J_2= 1.4 Hz, 3Z), 4.04 (q, 2H, *J* = 7.0 Hz, CH_2), 4.00 (dd, 0.65H, J_1=6.8, J_2= 1.9 Hz, 3E), 3.83-3.73 (m, 2H, 2β, 6a, 9), 3.73-3.56 (m, 4H, 6b, 3β, 5, 10, 12), 3.55-3.43 (m, 3H, 4, 8, 11), 1.97 (3 × s, 3H, 13), 1.22 (3×t, 3H, *J* = 7.0 Hz, CH_3) ppm. **HRMS** for adduct **11** $(C_{16}H_{31}O_{11}N_2Na)^+$ expected 450.1826 found 450.1821.

3.4. Enzymatic galactosylation of 6 enriched in *E*-isomer

A portion of **6** enriched in the open-chain (*E*)-oxime (93%, see Section 3.2 above) was then subjected to standard β4GalT1 enzymatic transformation conditions for an extended period of 6 days at 37 °C. The reaction mixture after transformation was then purified by HPLC. The ^{1}H-NMR spectrum of the purified mixture showed a doublet at 4.4 ppm that corresponds to the proton attached to the anomeric carbon of Gal with a β-1,4 linkage (Figure S2a). The relative integration of this proton environment compared to the CH_3 of the *N*-ethoxyamine portion of the molecule (set to 3.00) was

0.60 (60% extent of conversion into the LacNAc adduct). Successful conversion to the LacNAc product was confirmed by the observation of the product peak in the positive ion liquid chromatography-mass spectrometry (LC-MS); [**11** + Na]$^+$ (Figure S2b). From the isomer interconversion experiment on **6** (Section 3.2), the proportion of β-anomer at equilibrium in buffered D_2O was 0.18 (Figure S1). This means that up to 18% of GlcNAc should be available to conjugate with Gal after 144 h; however, 60% of LacNAc adduct was, in fact, produced. This difference may be related to the subsequent conjugation of Gal to GlcNAc adducts by the β4GalT1 enzyme during the equilibration period. This means that the presence of the enzyme may pull the mixture out of equilibrium, leading to fresh generation of more β-adduct.

Although a reactant mixture of **6** consisting of pure β-pyranoside could not be obtained to confirm it was the best (or only) substrate for β4GalT1, these observations suggest that the β-anomer of the adduct is the best substrate for the enzyme.

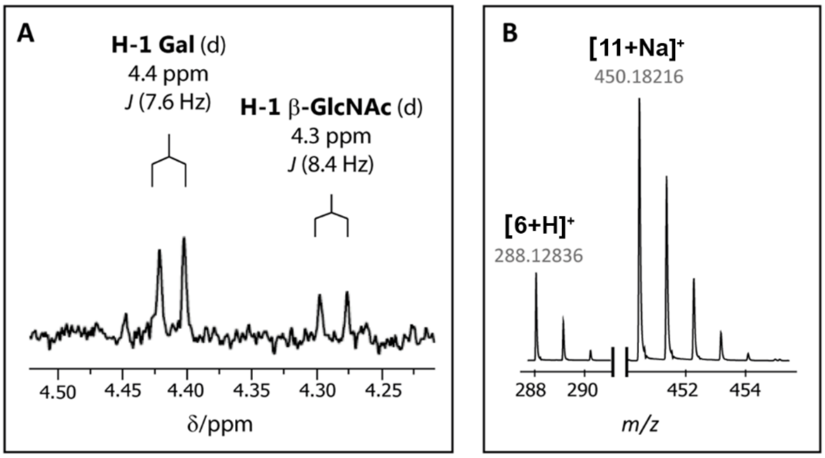

Figure S2: (a) ^{1}H-NMR (CD_3OD, 295 K, 400 MHz) spectrum of enzymatically produced **11** showing the appearance of a new doublet that correspond to the Gal H-1 from the disaccharide. (b) HRMS of the solution shows the presence of starting adduct **6** and enzymatically produced adduct **11**. The 100% intensity peaks are shown for both (the first ^{13}C isotopomer).

4. Synthesis of glycoconjugates of tether 3

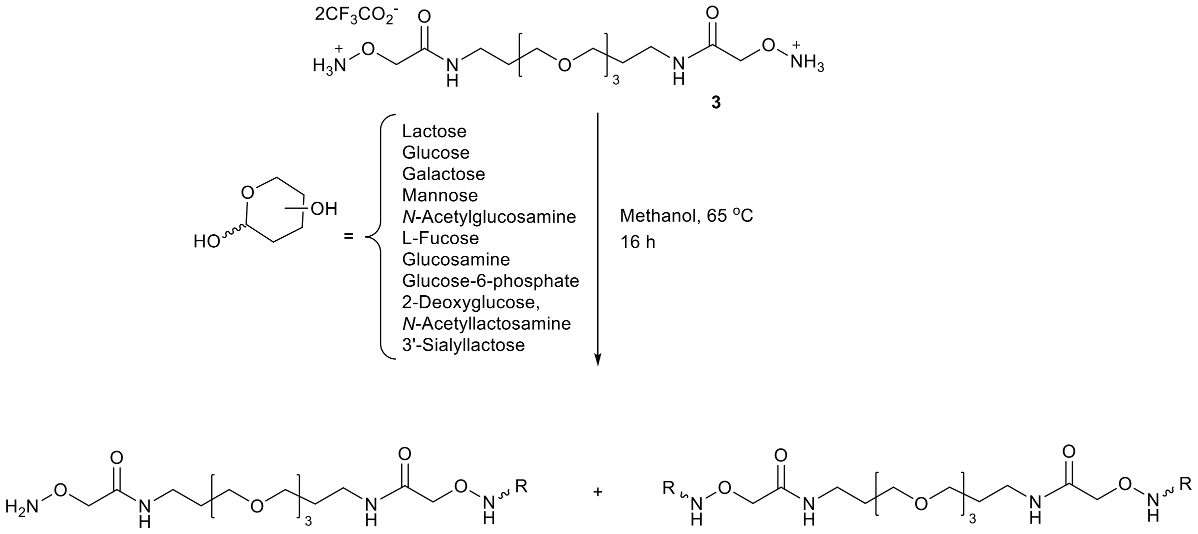

R = *N*-Acetylglucosaminyl, **S5**
R = Galactosyl, **7**
R = Glucosyl, **8**
R = Mannosyl, **9**
R = Lactosyl, **10**
R = L-Fucosyl, **S6**
R = Glucosaminyl, **S7**
R = Glucosyl-6-phosphate, **S8**
R = 2-Deoxyglucosyl, **S9**
R = *N*-Acetyllactosaminyl, **S10**
R = 3'-Sialyllactosyl, **S11**

R = *N*-Acetylglucosaminyl, **4**
R = L-Fucosyl, **S12**
R = Glucosaminyl, **S13**
R = Glucosyl-6-phosphate, **S14**
R = 2-Deoxyglucosyl, **S15**
R = *N*-Acetyllactosaminyl, **S16**
R = 3'-Sialyllactosyl, **S17**

Scheme S2: Synthesis of monomeric and dimeric glycoconjugates of tether **3**

4.1. Dimeric adducts with tether 3

Formation of **4**, adduct of **3** with GlcNAc

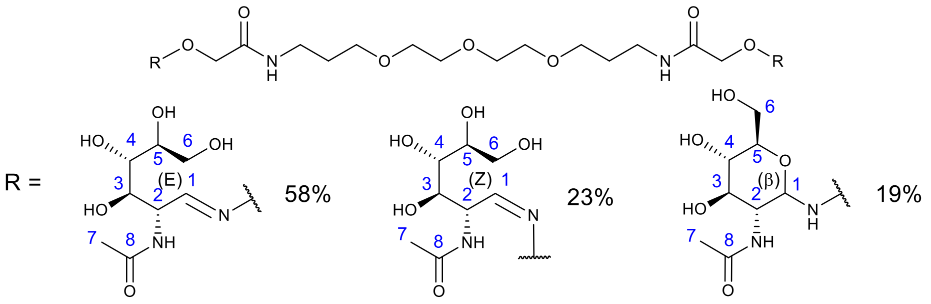

Yield: 25 mg, 15%, 58:23:0:19 mixture of E/Z/α/β-anomers.

The synthesis of compound **4** was accomplished by mixing 1 eq. of **3** with 2 eq. of GlcNAc in methanol (10 mL). The solution was stirred under reflux conditions overnight (65 °C, N_2 atmosphere). The solvent was removed under reduced pressure and the crude mixture was purified by HPLC on a 60 min gradient ranging from 5% to 95% MeCN in water with a flow rate of 1.0 mL/min. The sample was collected and freeze-dried to afford the product as an oil.

[1]**H-NMR** (400 MHz, CD_3OD): δ_H 7.67 (d, 1.08H, 5.6 Hz, 1E), 6.76 (d, 0.43H, 7.5 Hz, 1Z), 5.35 (t, 0.44H, 7.4 Hz, 2Z), 4.76 (t, 1.27H, 6.0 Hz, 2E), 4.59 (s, 0.61H, OCH_2CO Z), 4.49 (s, 2.12H, OCH_2CO E), 4.32 (d, 0.35H, *J* = 9.7 Hz, 1β), 4.17 (s, 0.60H, OCH_2CO β), 4.09 (m, 1.70H, 3E, 3Z), 3.92 (dd, 0.38H, J_1=11.8 Hz, J_2=1.5 Hz, 2β), 3.80 (dd, 2H, J_1=10.6 Hz, J_2=3.1 Hz, 6a), 3.74-3.44 (m, 20H, 3β, 4, 5, 6b, $(CH_2OCH_2)_3$), 3.38-3.31 (m, $NHCH_2$, CH_2NH), 2.02 (3 × s, 6H, 7E, 7Z, 7β), 1.82 (m, 4H, 2 × CH_2) ppm. [13]**C-NMR** (100 MHz, CD_3OD): δ_C 172.5 (CO), 172.0 (CO), 171.9 (CO), 171.4 (CO), 170.9 (CO), 170.8 (CO), 150.9 (C-1 E), 150.6 (C-1 Z), 90.1 (C-1 β), 77.9 (CH), 75.3 (CH), 73.3 (CH), 72.4 (CH), 72.1 (CH_2 β), 71.5 (CH_2 Z), 71.3 (CH_2 E), 70.5 (CH), 70.4 (CH), 70.1, 69.8 - 68.5 ($(CH_2OCH_2)_3$), 68.4 (CH), 63.4 (CH_2), 61.5 (C-2 β), 52.1 (C-2 E/Z), 36.3 (2 × $NHCH_2$), 28.9 (2 × CH_2), 21.6-21.3 (3 × C-7) ppm. **HRMS** for $(C_{30}H_{56}O_{17}N_6Na)^+$ expected 795.3594 found 795.3576.

4.2. Monomeric adducts with tether 3

General experimental procedure for adducts **7**–**10**

The synthesis of compounds **7**–**10** was accomplished by mixing 1 eq. of **3** with 0.5 eq. of the respective reducing sugar (lactose, glucose, galactose or mannose) in methanol (10 mL). The solution was stirred under reflux conditions overnight (65 °C, N_2 atmosphere). The solvent was removed under reduced pressure and the crude mixture was purified by HPLC on a 60 min gradient ranging from 5% to 95% MeCN in water with a flow rate of 1.0 mL/min. The sample was collected and freeze-dried to afford the product as oil.

Formation of monomeric adduct **7**, adduct of **3** with Gal

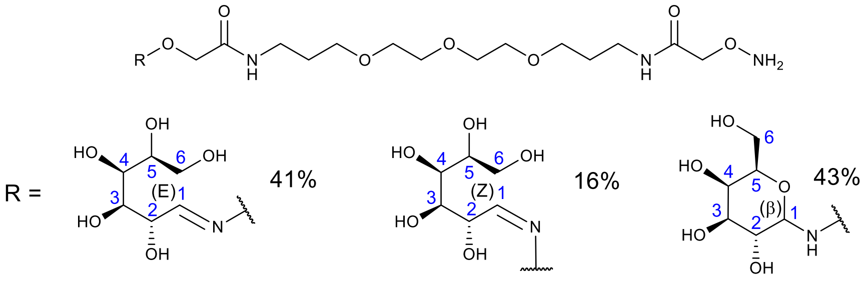

Yield: 23.9 mg, 33%, 41:16:0:43 mixture of E/Z/α/β-anomers.

^{1}H-NMR (400 MHz, CD_3OD): δ_H 7.70 (d, 0.41H, 6.4 Hz, 1E), 6.99 (d, 0.16H, 5.9 Hz, 1Z), 5.14 (t, 0.17H, 5.8 Hz, 2Z), 4.54-4.47 (m, 2.76H, OCH_2CO E, OCH_2CO Z, 2E), 4.17 (d, 0.43H, *J* = 8.7 Hz, 1β), 4.10 (s, 2H, OCH_2CO), 3.94 (dd, 1H, J_1=8.1 Hz, J_2=4.6 Hz, 6a), 3.90 (dd, J_1=9.2 Hz, J_2=2.5 Hz, 3E, 3Z), 3.73-3.51 (m, 14H, 3β, 6b, $(CH_2OCH_2)_3$), 3.41-3.31 (m, MeOH obscuring 4, 5, $NHCH_2$, CH_2NH), 1.87-1.77 (m, 4H, 2 × CH_2) ppm. **^{13}C-NMR** (100 MHz, CD_3OD): δ_C 171.5 (CO E, Z), 170.9 (CO β), 155.5 (C-1 E, Z), 153.9 (C-1 β), 74.1 (CH), 72.3 (OCH_2 E), 72.2 (OCH_2 β), 72.1 (OCH_2 Z), 71.3 (CH), 70.2 (CH), 70.1 (CH_2O), 70.0 (C-2 β), 69.9-68.6 ($(CH_2OCH_2)_3$), 68.4 (C-2 E), 63.5 (C-6), 36.4 ($NHCH_2$, CH_2NH), 28.9 (2 × CH_2) ppm. **HRMS** for $(C_{20}H_{40}O_{12}N_4Na)^+$ expected 551.2535 found 551.2526.

Formation of monomeric adduct **8**, adduct of **3** with Glc

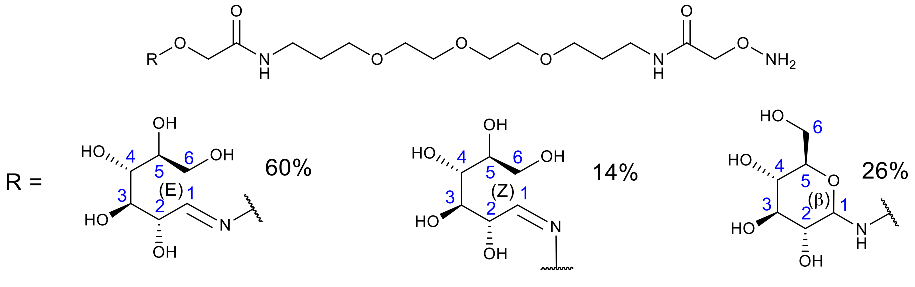

Yield: 9 mg, 11%, 60:14:0:26 mixture of E/Z/α/β-anomers.

^{1}H-NMR (400 MHz, CD_3OD): δ_H 7.63 (d, 0.60H, 6.8 Hz, 1E), 6.88 (d, 0.21H, 6.7 Hz, 1Z), 5.07 (t, 0.16H, 6.7 Hz, 2Z), 4.54 (d, 0.23H, *J* = 8.7 Hz, 1β), 4.49 (s, 1.25H, OCH_2CO E), 4.35 (t, 0.65H, 6.5 Hz, 2E), 4.20 (s, 0.13H, OCH_2CO Z), 4.10 (s, 1.83H, OCH_2CO β), 4.09 (m, 2H, $COCH_2O$, 3E, 3Z), 3.92 (d, 0.24H, *J* = 8.5 Hz, 2β), 3.81 (dd, 1H, J_1=11.1 Hz, J_2=3.2 Hz, 6a), 3.75-3.51 (m, 14H, 3β, 6b, $(CH_2OCH_2)_3$), 3.41-3.30 (m,

MeOH obscuring 4, 5, $NHCH_2$, CH_2NH), 1.87-1.77 (m, 4H, 2 × CH_2) ppm. **^{13}C-NMR** (100 MHz, CD_3OD): δ_C 171.5 (CO E, Z), 170.9 (CO β), 152.8 (C-1 E, Z), 78.1 (CH), 74.1 (CH), 72.1 (OCH_2 E), 71.5 (OCH_2 Z, β), 71.1 (OCH_2 β), 70.6 (CH), 70.1 (CH_2O, C-2 E), 69.9-68.6 ($(CH_2OCH_2)_3$), 63.5 (C-6), 36.4 ($NHCH_2$, CH_2NH), 28.9 (2 × CH_2) ppm. **HRMS** for $(C_{20}H_{40}O_{12}N_4Na)^+$ expected 551.2535 found 551.2534.

Formation of monomeric adduct **9**, adduct of **3** with Man

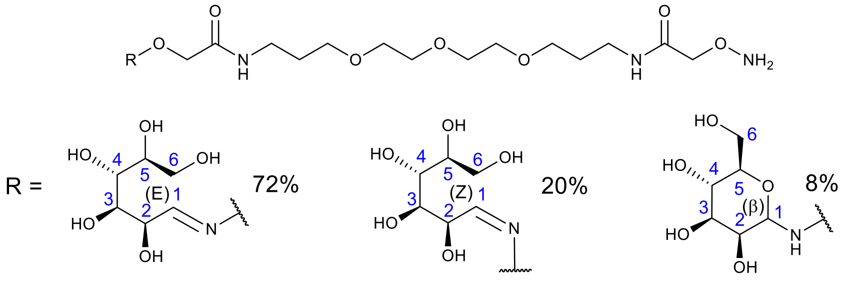

Yield: 8.8 mg, 12%, 72:20:0:8 mixture of E/Z/α/β-anomers.

^{1}H-NMR (500 MHz, CD_3OD): δ_H 7.64 (d, 0.72H, 7.1 Hz, 1E), 6.91 (d, 0.20H, 6.9 Hz, 1Z), 5.02 (t, 0.21H, 7.2 Hz, 2Z), 4.51 (s, 0.43H, OCH_2CO Z), 4.49 (s, 1.62H, OCH_2CO E), 4.24 (t, 0.90H, 7.1 Hz, 2E), 4.18 (d, 0.08H, J = 8.7 Hz, 1β), 4.09 (s, 2H, $COCH_2O$), 3.89 (dd, 1H, J_1=8.1 Hz, J_2=1.1 Hz, 6a), 3.83 (dd, 1H, J_1=11.1 Hz, J_2=3.3 Hz, 3E, 3Z), 3.77 (dd, 1H, J_1=8.1, J_2=1.3 Hz, 6b), 3.73-3.50 (m, 14H, 3β, $(CH_2OCH_2)_3$), 3.39-3.30 (m, MeOH obscuring 5, 4, $NHCH_2$, CH_2NH), 1.85-1.76 (m, 4H, 2 × CH_2) ppm. **^{13}C-NMR** (100 MHz, CD_3OD): δ_C 171.5 (CO E, Z), 170.9 (CO β), 154.5 (C-1 E), 153.7 (C-1 Z), 72.3 (C-3 E, Z), 72.0 (OCH_2 E), 71.5 (OCH_2 Z, β), 71.4 (C-1 β), 71.1 (OCH_2 β), 71.0 (CH), 70.1 (CH_2O), 69.9-68.6 ($(CH_2OCH_2)_3$), 68.3 (C-2 E), 63.7 (C-6), 36.4 ($NHCH_2$, CH_2NH), 28.9 (2 × CH_2) ppm. **HRMS** for $(C_{20}H_{40}O_{12}N_4Na)^+$ expected 551.2535 found 551.2525.

Formation of monomeric adduct **10**, adduct of **3** with Lac

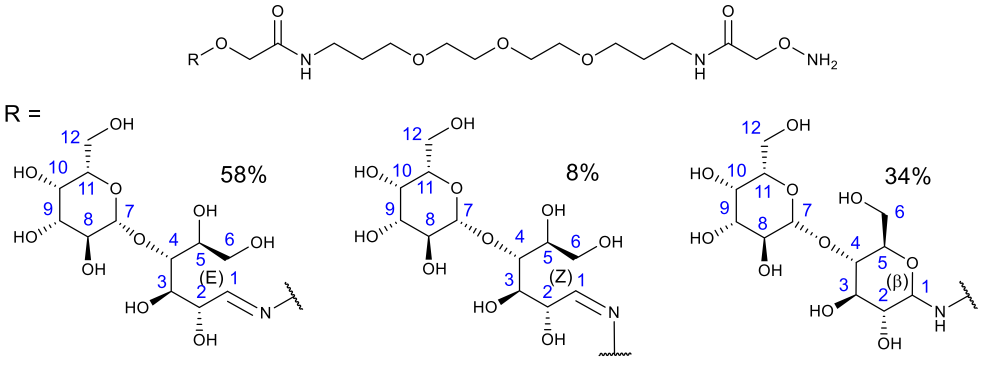

Yield: 34.8 mg, 11%, 58:8:0:34 mixture of E/Z/α/β-anomers.

^{1}H-NMR (400 MHz, CD_3OD): δ_H 7.72 (d, 0.57H, 6.4 Hz, 1E), 7.05 (d, 0.08H, 5.6 Hz, 1Z), 5.07 (t, 0.15H, 5.6 Hz, 2Z), 4.54 (m, 0.81H, 5.0 Hz, 2E, OCH_2CO Z), 4.50 (s, 1.05H, OCH_2CO E), 4.44 (d, 0.62H, J = 7.8 Hz, 7), 4.39 (d, 0.34H, J = 7.5 Hz, 1β), 4.24 (d, 0.88H, J = 3.5 Hz, 3β, OCH_2CO β), 4.10 (s, 2H, 3E, 3Z, $COCH_2O$), 3.71-3.47 (m, 20H, 6a, 6b, 8, 9, 10, 11, 12a, 12b, $(CH_2OCH_2)_3$), 3.41-3.30 (m, MeOH obscuring 4, 5, $NHCH_2$, CH_2NH), 1.87-1.77 (m, 4H, 2 × CH_2) ppm. **^{13}C-NMR** (100 MHz, CD_3OD): δ_C 171.5 (CO E, Z), 170.9

(CO β), 153.4 (C-1 E, C-1 Z), 104.2 (C-1 β), 103.7 (C-7), 91.5 (CH), 80.1 (CH), 79.2 (CH), 76.6 (CH), 75.9 (CH), 75.7 (CH), 75.6 (CH), 74.1 (CH_2), 73.5 (CH), 73.2 (CH_2), 72.1 (OCH_2 E), 71.6 (OCH_2 Z), 71.5 (OCH_2 β), 71.2 (CH), 70.1 (CH_2O), 69.9-68.6 ($(CH_2OCH_2)_3$), 36.5 (CH), 36.3 ($NHCH_2$), 36.2 (CH_2NH), 28.9 (2 × CH_2) ppm. **HRMS** for $(C_{26}H_{50}O_{17}N_4Na)^+$ expected 713.3063 found 713.3043.

4.3. Mixtures of monomeric and dimeric adducts with tether 3

General experimental procedure for adducts **S5**-**S22**

The synthesis of compounds **S5**-**S22** was accomplished by mixing 1 eq. of **3** with 0.5 eq. of the respective reducing sugar (*N*-acetylglucosamine, L-fucose, glucosamine, glucose-6-phosphate, 2-deoxyglucose, *N*-acetyllactosamine or 3'sialyllactose) in methanol (10 mL). The solution stirred under reflux conditions overnight (65 °C, N_2 atmosphere). The solvent was removed under reduced pressure and the crude mixture was purified by HPLC on a 60 min gradient ranging from 5% to 95% MeCN in water with a flow rate of 1.0 mL/min. The sample was collected and freeze-dried to afford the product as oil.

Formation of **S5** and **4**, adducts of **3** with GlcNAc

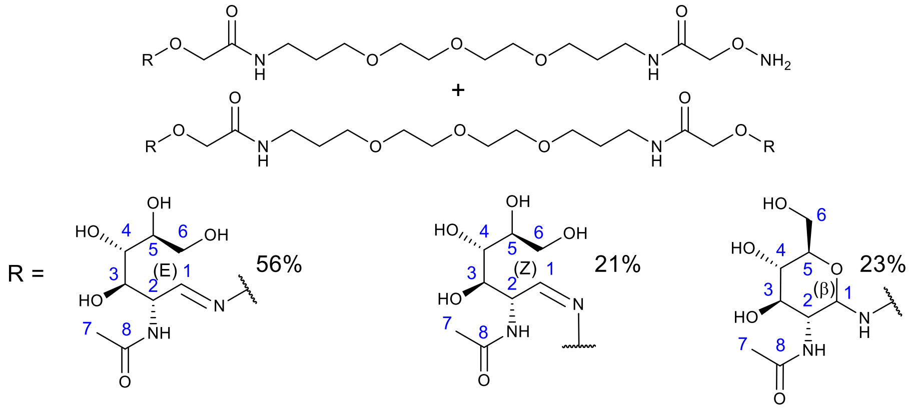

Yield: 30 mg, 70%, 56:21:0:23 mixture of E/Z/α/β-anomers.

^{1}H-NMR (400 MHz, CD_3OD): δ_H 7.67 (d, 0.56H, 5.6 Hz, 1E), 6.76 (d, 0.21H, 7.5 Hz, 1Z), 5.36 (t, 0.21H, 7.4 Hz, 2Z), 4.74 (t, 0.60H, 6.0 Hz, 2E), 4.59 (s, 0.55H, OCH_2CO Z), 4.50 (s, 1.05H, OCH_2CO E), 4.32 (d, 0.23H, J = 8.7 Hz, 1β), 4.18 (s, 0.37H, OCH_2CO β), 4.09 (m, 2.63H, $COCH_2O$, 3E, 3Z), 3.92 (d, 0.24H, J = 11.8 Hz, 2β), 3.80 (dd, 1H, J_1=10.4 Hz, J_2=3.0 Hz, 6a), 3.71-3.51 (m, 14H, 3β, 6b, $(CH_2OCH_2)_3$), 3.41-3.31 (m, MeOH obscuring 4, 5, $NHCH_2$, CH_2NH), 2.00 (3 × s, 3H, 7E, 7Z, 7β), 1.87-1.75 (m, 4H, 2 × CH_2) ppm. **^{13}C-NMR** (100 MHz, CD_3OD): δ_C 171.8 (CO E), 171.4 (CO Z), 170.9 (CO β), 150.9 (C-1 E), 150.6 (C-1 Z), 90.1 (C-1 β), 74.0 (CH), 72.1 (OCH_2 β), 71.4 (OCH_2 Z), 71.3 (OCH_2 E), 70.4 (CH), 70.1 (CH), 69.9 - 68.6 ($(CH_2OCH_2)_3$), 68.3 (CH), 63.4 (CH), 61.3 (C-2 β), 52.1 (C-2 E), 36.3 ($NHCH_2$, CH_2NH), 28.9 (2 × CH_2), 21.3 (C-7) ppm. **HRMS** for $(C_{22}H_{43}O_{12}N_5K)^+$ expected 608.2540 found 608.2525; for $(C_{30}H_{56}O_{17}N_6K)^+$ expected 811.3334 found 811.3311.

Formation of **S6** and **S12**, adducts of **3** with L-Fuc

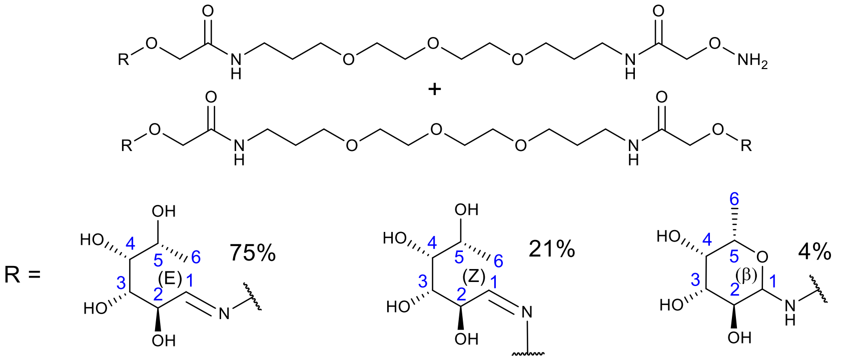

Yield: 35.6 mg, 52%, 75:21:0:4 mixture of E/Z/α/β-anomers.

^{1}H-NMR (400 MHz, CD_3OD): δ_H 7.66 (d, 0.75H, 6.4 Hz, 1E), 6.95 (d, 0.21H, 5.8 Hz, 1Z), 5.10 (t, 0.22H, 5.8 Hz, 2Z), 4.51-4.44 (m, 3H, 2E, OCH_2CO), 4.26 (d, 0.04H, *J* = 8.9 Hz, 1β), 4.10-4.02 (m, 3H, 5, $COCH_2O$), 3.83 (dd, 0.24H, J_1=9.1, J_2=2.5 Hz, 3Z), 3.68-3.49 (m, 13H, 3E, $(CH_2OCH_2)_3$), 3.43 (dd, 1H, J_1=9.0, J_2=1.6 Hz, 4), 3.38-3.28 (m, MeOH obscuring $NHCH_2$, CH_2NH), 1.84-1.73 (m, 4H, 4H, 2 × CH_2), 1.23 (2 × d, 3H, *J* = 6.6 Hz, 6β, 6E, 6Z) ppm. **^{13}C-NMR** (100 MHz, CD_3OD): δ_C 171.4 (CO E, Z), 170.9 (CO β), 155.5 (C-1 E), 153.9 (C-1 Z), 74.0 (CH), 73.3 (C-3 Z), 72.8 (C-3β), 72.1 (OCH_2 E), 71.6 (OCH_2 Z, β), 70.2 (C-4), 70.1 (CH_2O), 69.9-68.6 ($(CH_2OCH_2)_3$), 68.6 (CH), 68.4 (C-2 β), 36.4 ($NHCH_2$), 36.3 (CH_2NH), 28.9 (2 × CH_2), 18.6 (C-6) ppm. **HRMS** for $(C_{20}H_{40}O_{11}N_4Na)^+$ expected 535.2586 found 535.2573; $(C_{26}H_{50}O_{15}N_4Na)^+$ expected 681.3165 found 681.3160.

Formation of **S7** and **S13**, adducts of **3** with GlcN

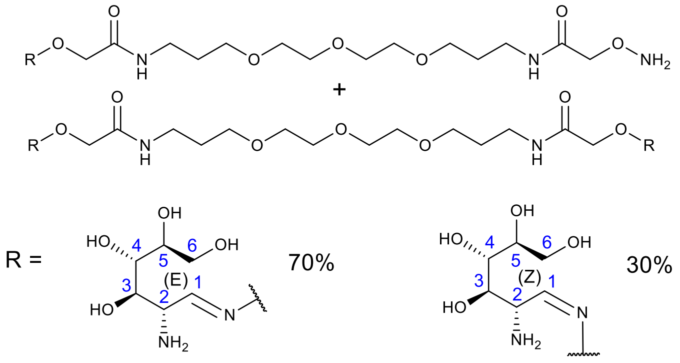

Yield: 14.2 mg, 22%, 70:30:0:0 mixture of E/Z/α/β-anomers.

^{1}H-NMR (400 MHz, CD_3OD): δ_H 7.72 (d, 0.70H, *J* = 4.3 Hz, 1E), 7.02 (d, 0.30H, *J* = 6.7 Hz, 1Z), 4.71-4.66 (m, 1H, 2Z, OCH_2CO Z), 4.59 (s, 1.32H, OCH_2CO E), 4.21-4.15 (m, 1H, 2E, 3E, 3Z), 3.79 (dd, 1H, J_1=6.3 Hz, J_2=1.5 Hz, 6a), 4.09 (s, 2H, $COCH_2O$), 3.79 (dd, 1H, J_1=10.9, J_2=3.2 Hz, 6b), 3.75-3.50 (m, 14H, 4, 5, $(CH_2OCH_2)_3$), 3.39-3.33 (m, $NHCH_2$, CH_2NH), 1.85-1.75 (m, 4H, 2 × CH_2) ppm. **^{13}C-NMR** (100 MHz, CD_3OD): δ_C 171.4 (CO E), 170.2 (CO Z), 146.8 (C-1 E, Z), 74.0 (CH), 72.5 (OCH_2 E), 71.3 (OCH_2 Z), 71.0 (CH), 70.0 (CH_2O), 69.8-68.6 ($(CH_2OCH_2)_3$), 68.4 (CH), 63.1 (CH_2), 36.4 ($NHCH_2$, CH_2NH), 28.9 (2 × CH_2) ppm. **HRMS** for $(C_{20}H_{41}O_{11}N_5Na)^+$ expected 550.2695 found 550.2685; $(C_{26}H_{52}O_{15}N_6Na)^+$ expected 711.3383 found 711.3379.

Formation of **S8** and **S14**, adducts of **3** with Glc-6-P

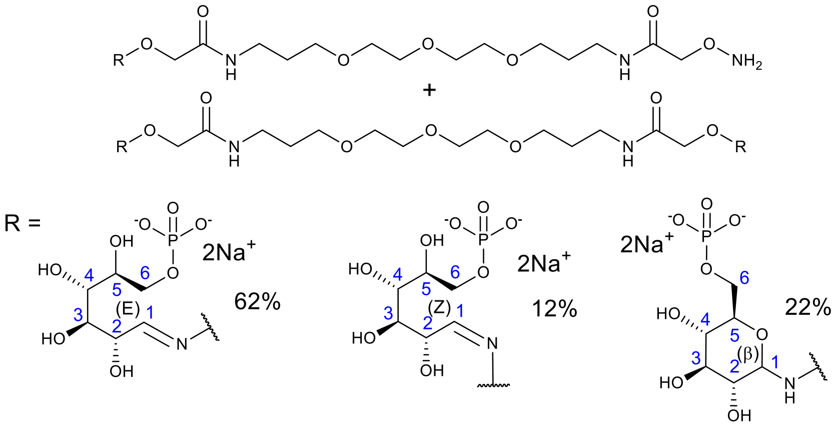

Yield: 11.2 mg, 22%, 66:12:0:22 mixture of E/Z/α/β-anomers.

^{1}H-NMR (400 MHz, D_2O): δ_H 7.75 (d, 0.66H, J = 6.3 Hz, 1E), 7.06 (d, 0.12H, J = 6.6 Hz, 1Z), 5.12 (t, 0.15H, J = 6.5 Hz, 2Z), 4.65 (s, 3.6H, OCH_2CO E, OCH_2CO Z, $COCH_2O$), 4.48 (t, 0.75H, J = 6.6 Hz, 2E), 4.39 (d, 0.22H, J = 9.2 Hz, 1β), 4.34 (s, 0.4H, OCH_2CO β), 4.23-3.90 (m, 3H, 6a, 6b, 3E, 3Z), 3.78-3.44 (m, 15H, 3β, 4, 5, $(CH_2OCH_2)_3$), 3.39 (t, 4H, J = 6.7Hz, $NHCH_2$, CH_2NH), 3.31 (t, 0.62H J = 8.4 Hz, 2β), 1.93-1.79 (m, 4H, 2 × CH_2) ppm. **^{13}C-NMR** (100 MHz, D_2O): δ_C 171.9 (CO E, Z), 168.5 (CO β), 152.9 (C-1 E), 95.9 (C-1 Z), 92.1 (C-1 β), 75.4 (CH), 73.9 (CH), 71.9 (OCH_2 E), 71.7 (OCH_2 Z, β), 71.3 (OCH_2 β), 70.8 (CH), 70.1 (CH_2O), 69.6-68.3 ($(CH_2OCH_2)_3$), 64.1 (CH_2), 36.3 ($NHCH_2$), 36.2 (CH_2NH), 28.2 (2 × CH_2) ppm. **HRMS** for $(C_{20}H_{40}O_{15}N_4P)^-$ expected 607.2228 found 607.2213; $(C_{26}H_{51}O_{23}N_4P_2)^-$ expected 849.2419 found 849.2402.

Formation of **S9** and **S15**, adducts of **3** with 2-deoxyGlc

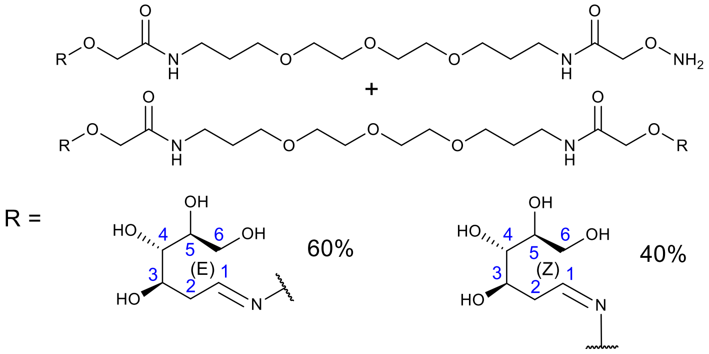

Yield: 19.5 mg, 48%, 60:40:0:0 mixture of E/Z/α/β-anomers.

^{1}H-NMR (400 MHz, CD_3OD): δ_H 7.69 (t, 0.60H, J = 6.3 Hz, 1E), 6.76 (d, 0.40H, J = 5.7 Hz, 1Z), 4.51 (s, 0.72H, OCH_2CO Z), 4.45 (s, 1.07H, OCH_2CO E), 4.10 (m, 3H, 3E, 3Z, $COCH_2O$), 3.80 (dt, 1H, J_1=10.9 Hz, J_2=3.7 Hz, 6a), 3.73-3.51 (m, 14H, 5, 6b, $(CH_2OCH_2)_3$), 3.41-3.31 (m, MeOH obscuring 4, $NHCH_2$, CH_2NH), 2.84 (ddd, 0.40H, J_1=15.0, J_2=8.9, J_3=5.9 Hz, 2a Z), 2.53 (m, 1.60H, 2a E, 2b E, 2a Z), 1.87-1.75 (m, 4H, 2 × CH_2) ppm. **^{13}C-NMR** (100 MHz, CD_3OD): δ_C 171.5 (CO E), 171.1 (CO Z), 151.8 (C-1 E), 151.5 (C-1 Z), 72.8 (CH), 72.1 (OCH_2 E), 71.6 (OCH_2 Z), 70.1 (CH_2O), 69.9-68.6 ($(CH_2OCH_2)_3$), 68.1 (CH), 67.6 (CH), 63.7

(CH), 36.4 ($NHCH_2$), 36.3 (CH_2NH), 33.7 (CH), 30.3 (CH), 29.3 (CH), 28.9 (2 × CH_2) ppm. **HRMS** for $(C_{20}H_{40}O_{11}N_4Na)^+$ expected 535.2586 found 535.2573; $(C_{26}H_{50}O_{15}N_4Na)^+$ expected 681.3165 found 681.3155.

Formation of **S10** and **S16**, adducts of **3** with LacNAc

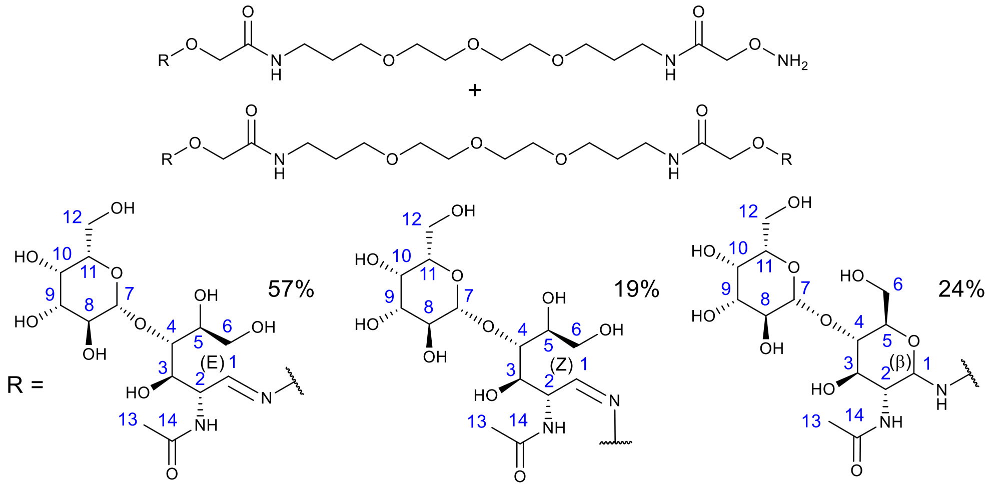

Yield: 18.1 mg, 67%, 57:19:0:24 mixture of E/Z/α/β-anomers.

^{1}H-NMR (400 MHz, CD_3OD): δ_H 7.72 (d, 0.57H, 5.7 Hz, 1E), 6.99 (d, 0.19H, 6.9 Hz, 1Z), 5.47 (t, 0.18H, 5.8 Hz, 2Z), 5.01 (t, 0.55H, 5.8 Hz, 2E), 4.53 (s, 0.35H, OCH_2CO Z), 4.46 (s, 0.88H, OCH_2CO E), 4.40 (d, 1H, J = 7.6 Hz, 7), 4.36 (s, 0.26H, OCH_2CO β), 4.30 (d, 0.18H, J = 9.7 Hz, 1β), 4.14 (s, 0.31H, $COCH_2O$ E), 4.11 (t, 0.21H, J = 5.1 Hz, 3Z), 4.08-4.02 (m, 2.70H, 3E, $COCH_2O$ Z, $COCH_2O$ β), 3.95-3.44 (m, 22H, 2β, 3β, 4, 5, 6, 8, 9, 10, 11, 12, $(CH_2OCH_2)_3$), 3.39-3.26 (m, MeOH obscuring $NHCH_2$, CH_2NH), 2.04-1.88 (6×s, 3H, 13), 1.84-1.73 (m, 4H, 2 × CH_2) ppm. **^{13}C-NMR** (100 MHz, CD_3OD): δ_C 172.0 (CO), 171.5 (CO E, Z), 171.0 (CO β), 151.6 (C-1 E), 151.5 (C-1 Z), 104.3 (C-7), 104.1 (CH), 103.7 (CH_2), 79.4 (C-1 β), 76.5 (CH), 75.8 (CH), 75.7 (CH), 74.1 (OCH_2 Z), 73.5 (CH), 72.5 (OCH_2 E), 72.2 (OCH_2 β), 71.4 (CH), 71.3 (CH), 70.2 (CH), 70.1 (CH_2O), 69.9-68.6 ($(CH_2OCH_2)_3$), 62.3 (CH), 50.9 (CH), 36.3 ($NHCH_2$), 36.1 (CH_2NH), 29.0 (CH_2), 28.9 (CH_2), 21.6 (C-13 β), 21.3 (C-13 E/Z) ppm. **HRMS** for $(C_{28}H_{54}O_{17}N_5)^+$ expected 732.3509 found 732.3514; for $(C_{42}H_{77}O_{27}N_6)^+$ expected 1097.4831 found 1097.4837.

Formation of **S11** and **S17**, adducts of **3** with 3'-SL

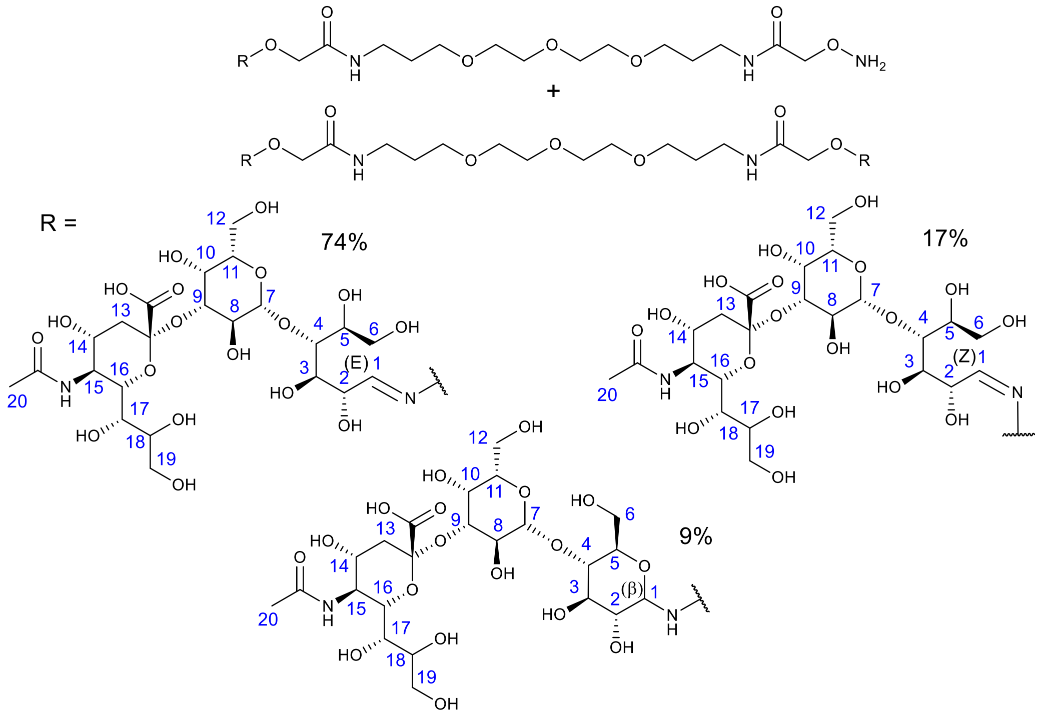

Yield: 30.6 mg, 79%, 74:17:0:9 mixture of E/Z/α/β-anomers.

^{1}H NMR (400 MHz, CD_3OD): δ_H 7.68 (d, 0.74H, 6.3 Hz, 1E), 7.02 (d, 0.17H, 5.7 Hz, 1Z), 5.03 (t, 0.21H, 4.6 Hz, 2Z), 4.55-4.48 (m, 1.70H, 2E, OCH_2CO Z), 4.47 (s, 2H, $COCH_2O$), 4.40 (d, 1H, J = 7.6 Hz, 7), 4.36 (s, 0.63H, OCH_2CO E), 4.34-4.29 (m, 1.09H, 1β, 16), 4.27 (s, 0.29, OCH_2CO β), 4.02 (dd, 1H, J_1=9.7, J_2=3.0 Hz, 6a), 3.91-3.43 (m, 29H, 2β, 3-5, 6b, 8-12, 14, 15, 17-19, $(CH_2OCH_2)_3$), 3.37-3.25 (m, MeOH obscuring $NHCH_2$, CH_2NH), 2.78-2.71 (m, 1H, 13a), 2.00 (2×s, 3H, 20), 1.93-1.85 (m, 1H, 13b), 1.83-1.72 (m, 4H, 2 × CH_2) ppm. **^{13}C-NMR** (100 MHz, CD_3OD): δ_C 174.8 (CO), 171.8 (CO), 171.0 (CO), 170.4 (CO), 153.4 (C-1 E), 104.2 (C-7), 98.6 (CH), 80.0 (C-1 β), 75.6 (CH), 73.5 (CH), 72.1 (OCH_2 E), 71.6 (CH), 71.4, 70.1 (CH_2O), 69.8-68.6 ($(CH_2OCH_2)_3$), 67.3 (CH), 63.4 (CH_2), 61.1 (CH_2), 52.3 (CH), 42.1 (CH_2), 35.9 ($NHCH_2$), 29.0 (CH_2), 21.3 (CH_3) ppm. **HRMS** for $(C_{37}H_{67}O_{25}N_5Na)^+$ expected 1004.4017 found 1004.4023; $(C_{60}H_{104}O_{43}N_6Na)^+$ expected 1619.6028 found 1619.6032.

5. Synthesis of glycoconjugates of lipid 1

5.1. Synthesis of glycolipid 2, adduct of lipid 1 with GlcNAc

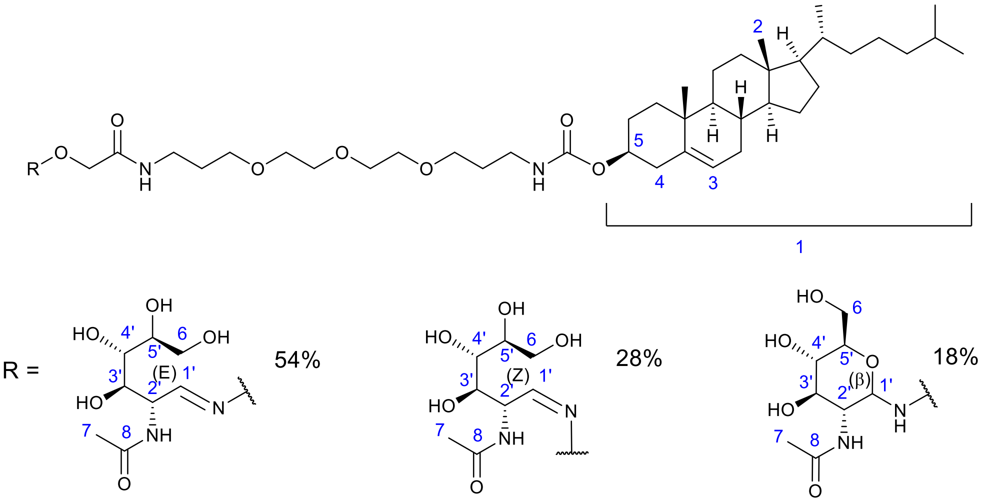

Yield: 49%, 31 mg, 54:28:0:18 mixture of E/Z/α/β-anomers.

Lipid **1** (1 eq.) and GlcNAc (2 eq.) were dissolved in MeOH (10 mL) under reflux conditions (N_2 atmosphere, 65 °C, overnight). The next day, the solvent was removed under reduced pressure and the crude mixture was purified by silica column chromatography (DCM/MeCN/MeOH 5:5:1 changed to MeOH to recover the product). **TLC** R_f 0 (DCM/MeCN/MeOH 5:5:1).

^{1}H-NMR (500 MHz, CD_3OD): δ_H 7.66 (d, 0.57H, *J* = 5.6 Hz, 1'E), 6.74 (d, 0.29H, *J* = 7.5 Hz, 1'Z), 5.40 (d, 1H, *J* = 4.6 Hz, 3), 5.34 (t, 0.32H, *J* = 7.4 Hz, 2'Z), 4.75 (t, 0.65H, *J* = 6.1 Hz, 2'E), 4.57 (s, 0.57H, OCH_2CO Z), 4.48 (s, 1.11H, OCH_2CO E), 4.38 (m, 1H, 5 cholesteryl), 4.30 (d, 0.19H, *J* = 9.8 Hz, 1'β), 4.16 (s, 0.29H, OCH_2CO β), 4.08 (dd, 0.86H, J_1=6.6 and J_2=2.1 Hz, 3'E, 3'Z), 3.91 (d, 0.22H, *J* = 11.8 Hz, 2'β), 3.79 (dd, 1H, J_1=10.7 and J_2=3.3 Hz, 6a GlcNAc), 3.71-3.48 (m, 15H, $(CH_2OCH_2)_3$, 3'β, 4', 5' and 6b GlcNAc) 3.36 (t, 2H, *J* = 3.8 Hz, CH_2NH), 3.19 (t, 2H, *J* = 6.8 Hz, $NHCH_2$), 2.38-2.27 (m, 2H, 4), 2.10-0.87 (m, 45H, 1 cholesteryl, 2 × CH_2, 7), 0.74 (s, 3H, 2) ppm. **^{13}C-NMR** (100 MHz, CD_3OD): δ_C 172.0 (C-8 E), 171.8 (C-8 Z), 170.8 (C-8 β), 170.7 (CONH), 157.1 (COO), 150.9 (C-1' E), 150.6 (C-1' Z), 139.8 (C-1), 122.0 (C-3), 74.0 (C-5), 72.4 ($NHOCH_2$ β), 72.1 ($NHOCH_2$ E/Z), 71.4 (CH), 71.3 (C-3' β), 71.0 (CH), 70.4 (C-3' E/Z), 70.1 - 69.8 ($(CH_2OCH_2)_3$), 68.9 (CH), 68.6 (CH), 68.4 (CH_2), 63.4 (CH_2), 56.7 (C), 56.1 (C), 52.1 (C-2' Z), 50.2 (CH), 48.6 (C-2' E), 42.1 (C), 39.7 (CH_2), 39.3 (CH_2), 38.3 (C-4), 37.6 (CH_2NH), 36.9 ($NHCH_2$), 36.4 (CH_2), 36.3 (C), 36.0 (CH_2), 35.7 (CH), 31.8 (CH), 31.6 (C), 29.0 (CH_2 spacer), 28.9 (CH_2 spacer), 27.9 (CH_2), 27.7 (CH), 23.9 (CH_2), 23.6 (CH_2), 21.8 (CH_3), 21.6 (C-7), 21.3 (CH_3), 21.2 (CH_2), 20.8 (CH_2) 18.4 (CH_2), 11.0 (C-2) ppm. **HRMS** for $(C_{48}H_{84}O_{12}N_4Na)^+$ 931.5983 found 931.5978.

5.2. Synthesis of glycolipid 13, adduct of lipid 1 with LacNAc

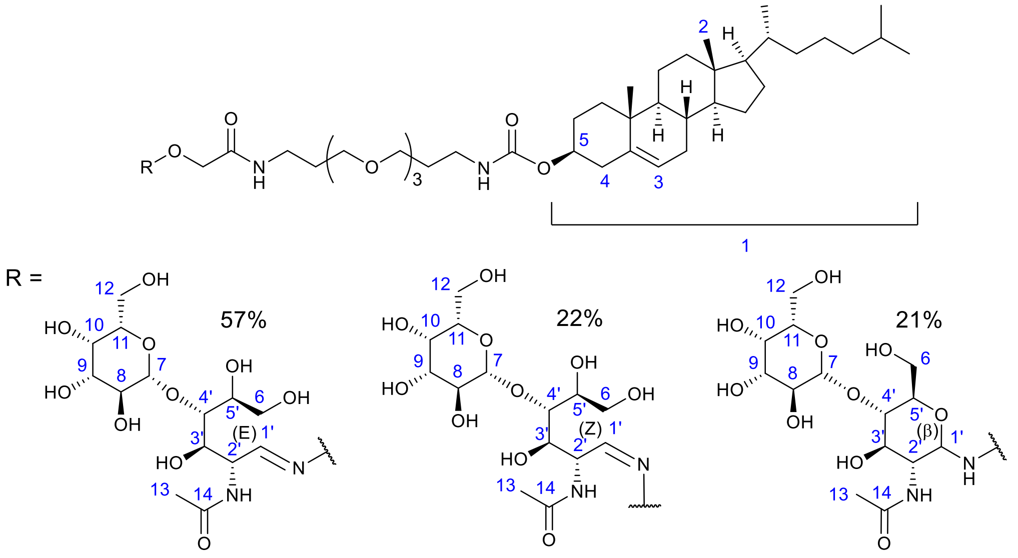

Yield: 20%, 7.3 mg, 49:29:0:22 mixture of E/Z/α/β-anomers.

Lipid **1** (1 eq.) and LacNAc (2 eq.) were dissolved in MeOH (10 mL) under reflux conditions (N_2 atmosphere, 65 °C, overnight). The next day, the solvent was removed under reduced pressure and the crude mixture was purified by silica column chromatography (DCM/MeCN/MeOH 5:5:1 changed to MeOH to recover the product). **TLC** R_f 0 (DCM/MeCN/MeOH 5:5:1).

^{1}H-NMR (400 MHz, CD_3OD): δ_H 7.72 (d, 0.49H, J = 5.7 Hz, 1'E), 6.98 (d, 0.29H, J = 6.9 Hz, 1'Z), 5.47 (t, 0.31H, J = 6.9 Hz, 2'Z), 5.38 (d, 1H, J = 4.2 Hz, 3), 5.01 (t, 0.63H, J = 6.0 Hz, 2'E), 4.55 (s, 0.62H, OCH_2CO Z), 4.48 (s, 0.92H, OCH_2CO E), 4.43 (d, 0.48H, 7.7 Hz, 7), 4.36 (m, 1H, 5), 4.29 (d, 0.22H, J = 9.7 Hz, 1'β), 4.16 (s, 0.17H, OCH_2CO β), 4.11 (t, 0.33H, J = 4.3 Hz, 2'β), 4.04 (dd, 0.58H, J_1=7.0 and J_2=2.4 Hz, 3'E, 3'Z), 3.84 (dd, 1H, J_1=11.7 and J_2=7.6 Hz, 6a), 3.78 (dd, 2H, J_1=8.4 and J_2=2.9 Hz, 12), 3.72 (dd, 1H, J_1=11.4 and J_2=4.3 Hz, 6b), 3.66-3.43 (m, 18H, $(CH_2OCH_2)_3$, 4', 5', 8, 9, 10, 11, 3β), 3.34 (m, 2H, CH_2NHCOO), 3.16 (t, 2H, J = 6.7 Hz, $CONHCH_2$), 2.30 (m, 2H, 4 cholesteryl), 2.08-0.82 (m, 50H, 1, 2 × CH_2 trioxa, 7), 0.71 (s, 3H, 2) ppm. **^{13}C-NMR** (100 MHz, CD_3OD): δ_C 178.3 (C-14 E), 171.8 (C-14 Z), 170.8 (C-14 β), 170.7 (CONH), 157.2 (COO), 151.5 (C-1' E), 145.7 (C-1' Z), 139.8 (C), 122.0 (C-3), 104.1 (C-7), 75.7 ($NHOCH_2$), 74.0 (C-5), 71.3 (C-3), 70.1-69.8 ($(CH_2OCH_2)_3$), 68.9 (CH), 68.6 (CH), 68.4 (CH_2), 56.7 (CH), 56.1 (C), 50.9 (C-2' Z), 50.2 (C-2' E), 42.0 (C), 39.7 (CH_2), 39.2 (CH_2), 38.3 (C-4), 37.6 (CH_2NH), 36.8 ($NHCH_2$), 36.3 (C), 35.9 (CH_2), 35.7 (CH), 31.8 (CH_2), 31.6 (C), 29.4 (CH_2 spacer), 29.0 (CH_2 spacer), 27.9 (CH_2), 27.7 (CH), 23.9 (CH_2), 23.5 (CH_2), 23.3 (CH_3), 21.8 (CH_3), 21.5 (C-13), 21.2 (CH_2), 20.7 (CH_3), 18.4 (CH_3), 10.9 (C-2) ppm. **HRMS** for $(C_{54}H_{93}O_{17}N_4)^-$ 1069.6536 found 1069.6541.

5.3. HRMS data

Table S1: Calculated errors in HRMS measurements.

Compound	Molecular formula	Expected m/z	Found m/z	Error / ppm
**S3**	$(C_{24}H_{46}N_4O_{11}Na)^+$	589.3055	589.305	0.848456361
**3**	$(C_{14}H_{31}N_4O_7)^+$	367.2186	367.2187	-0.272317361
**S2**	$(C_{38}H_{68}O_5N_2K)^+$	671.4765	671.476	0.744627697
**S4**	$(C_{45}H_{79}O_9N_3K)^+$	844.5453	844.5448	0.592034554
**1**	$(C_{40}H_{71}O_7N_3K)^+$	744.4929	744.4924	0.671598077
**6**	$(C_{10}H_{20}O_6N_2Na)^+$	287.1214	287.1211	1.044854197
**11**	$(C_{16}H_{31}O_{11}N_2Na)^+$	450.1826	450.1821	1.11066043
**4**	$(C_{30}H_{56}O_{17}N_6Na)^+$	795.3594	795.3576	2.263127839
**10**	$(C_{20}H_{40}O_{12}N_4Na)^+$	551.2535	551.2534	0.181404744
**8**	$(C_{20}H_{40}O_{12}N_4Na)^+$	551.2535	551.2526	1.632642695
**7**	$(C_{20}H_{40}O_{12}N_4Na)^+$	551.2535	551.2525	1.814047439
**9**	$(C_{26}H_{50}O_{17}N_4Na)^+$	713.3063	713.3043	2.803844576
**S5**	$(C_{22}H_{43}O_{12}N_5K)^+$	608.254	608.2525	2.466075028
**4**	$(C_{30}H_{56}O_{17}N_6K)^+$	811.3334	811.3311	2.834839537
**S6**	$(C_{20}H_{40}O_{11}N_4Na)^+$	535.2586	535.2573	2.428732579
**S12**	$(C_{26}H_{50}O_{15}N_4Na)^+$	681.3165	681.316	0.733873317
**S7**	$(C_{20}H_{41}O_{11}N_5Na)^+$	550.2695	550.2685	1.817291345
**S13**	$(C_{26}H_{52}O_{15}N_6Na)^+$	711.3383	711.3379	0.562320347
**S8**	$(C_{20}H_{40}O_{15}N_4P)^-$	607.2228	607.2213	2.470262974
**S14**	$(C_{26}H_{51}O_{23}N_4P_2)^-$	849.2419	849.2402	2.001785357
**S9**	$(C_{20}H_{40}O_{11}N_4Na)^+$	535.2586	535.2573	2.428732579
**S15**	$(C_{26}H_{50}O_{15}N_4Na)^+$	681.3165	681.3155	1.467746635
**S10**	$(C_{28}H_{54}O_{17}N_5)^+$	732.3509	732.3514	-0.682732827
**S16**	$(C_{42}H_{77}O_{27}N_6)^+$	1097.4831	1097.4837	-0.546705457
**S11**	$(C_{37}H_{67}O_{25}N_5Na)^+$	1004.4017	1004.4023	-0.597370554
**S17**	$(C_{60}H_{104}O_{43}N_6Na)^+$	1619.6028	1619.6032	-0.246974135
**2**	$(C_{48}H_{84}O_{12}N_4Na)^+$	931.5983	931.5978	0.536712014
**13**	$(C_{54}H_{93}O_{17}N_4)^-$	1069.6536	1069.6541	-0.467441048

6. NMR spectra for intermediate S2 and new compounds S3, 3, S4 and 1

^{1}H-NMR spectrum of S3 in $CDCl_3$ at 298 K.

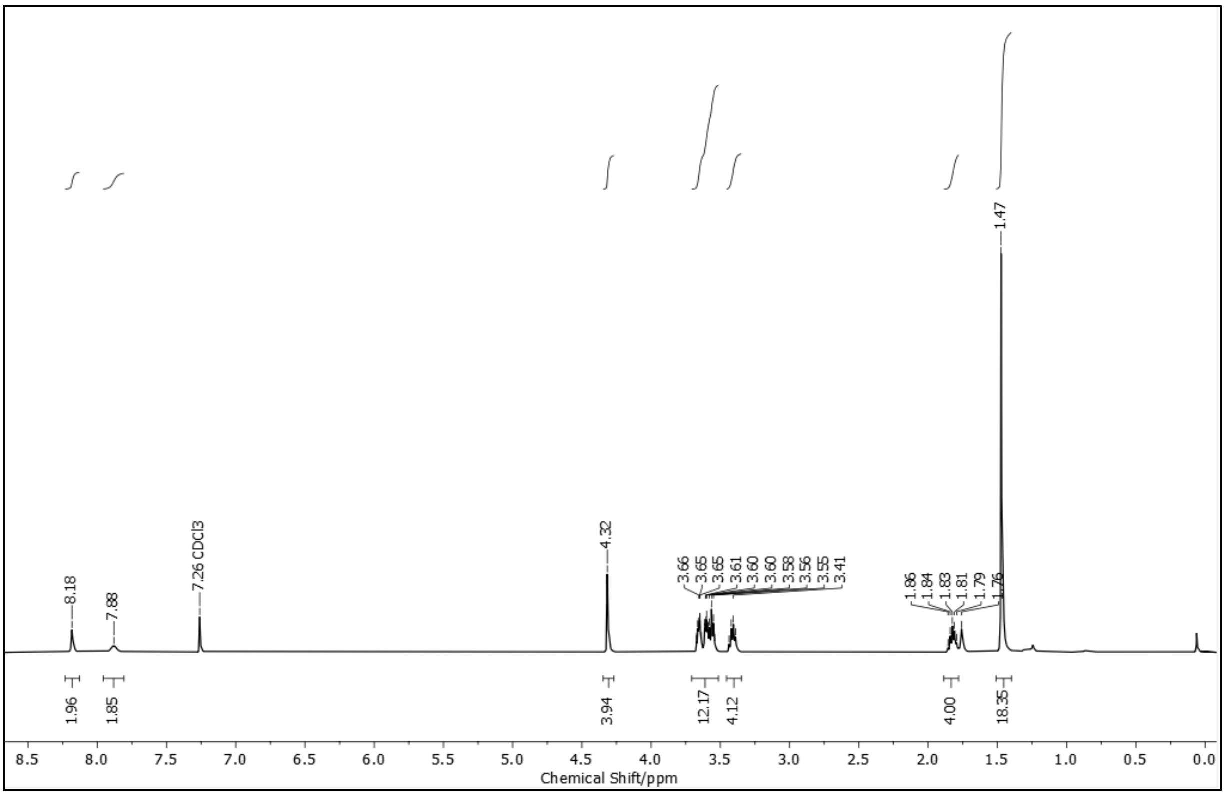

^{13}C-NMR spectrum of S3 in $CDCl_3$ at 298 K.

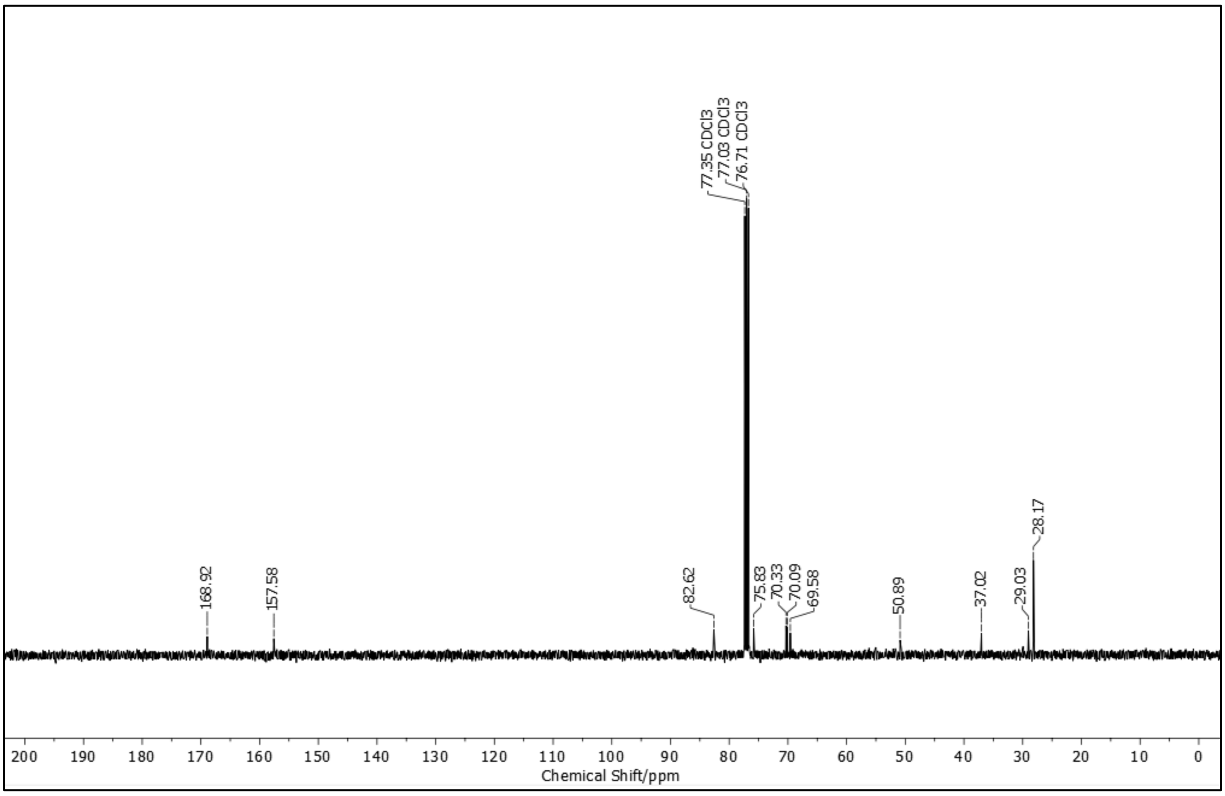

^{1}H-NMR spectrum of 3 in D_2O at 298 K.

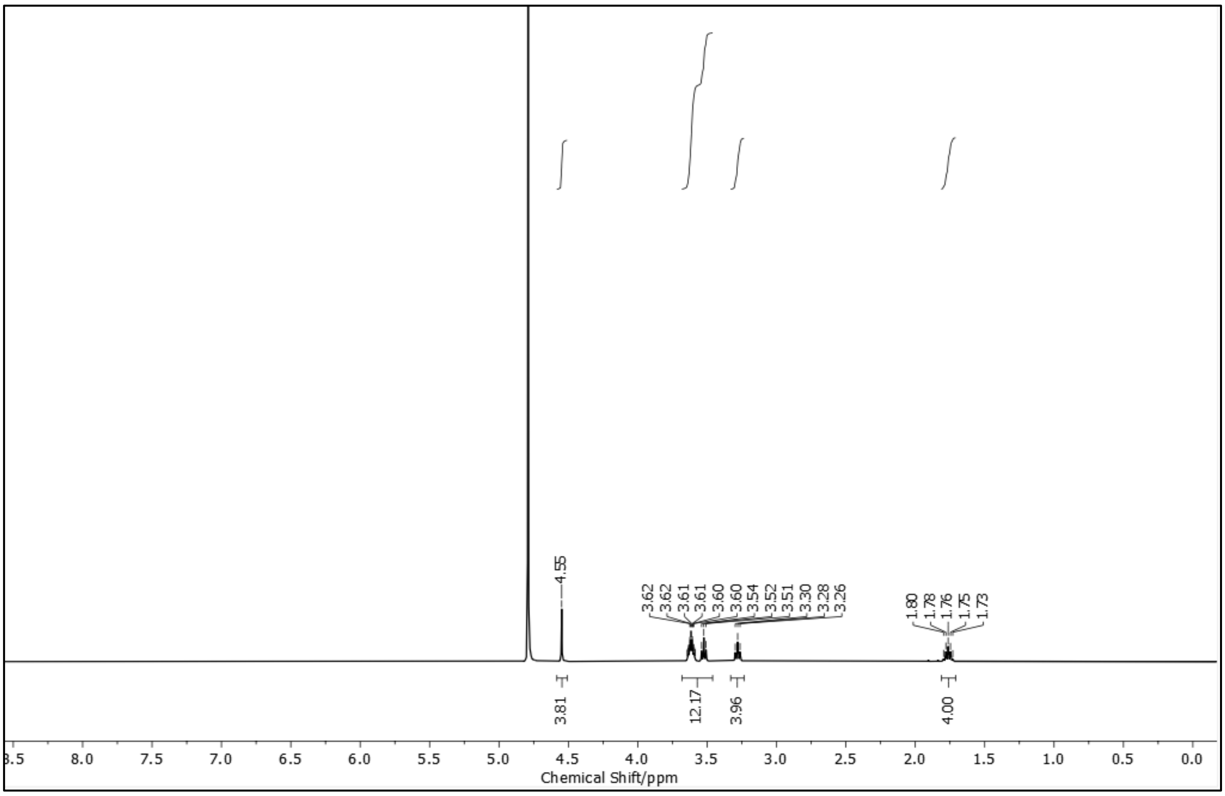

^{13}C-NMR spectrum of 3 in D_2O at 298 K.

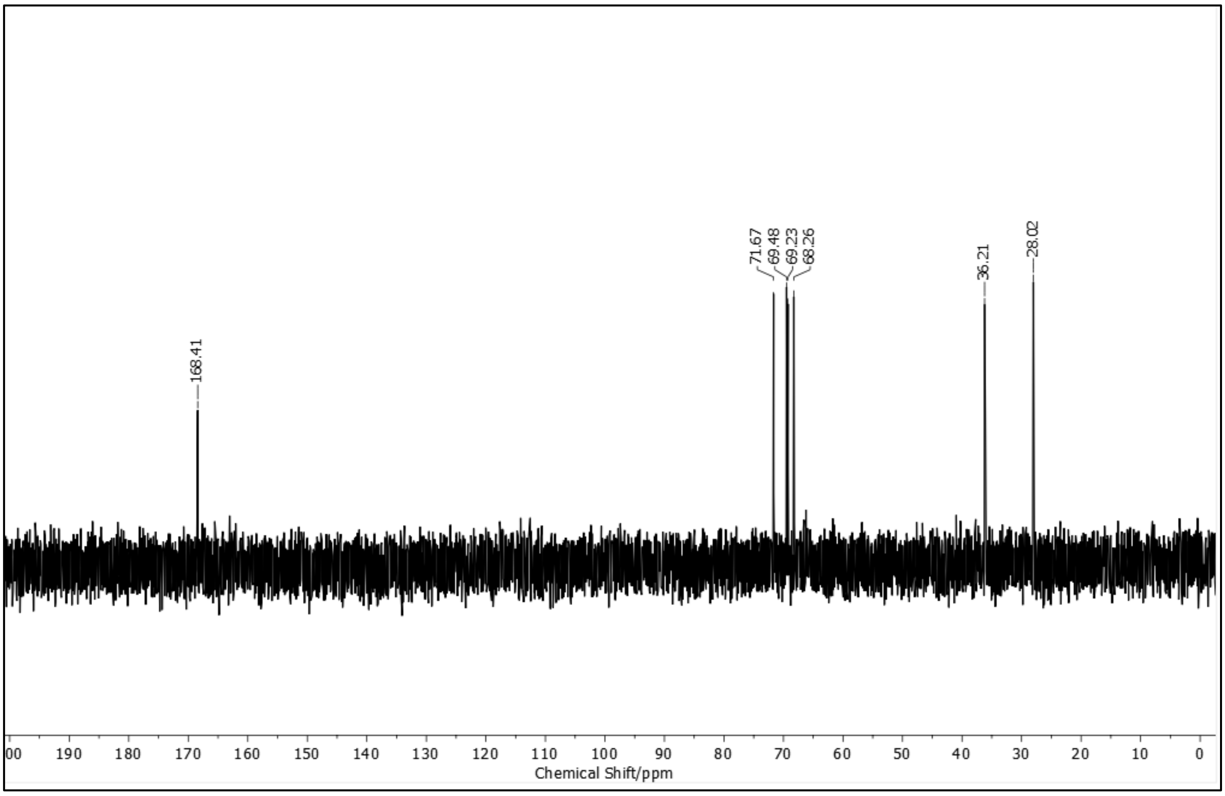

^{1}H-NMR spectrum of S2 in $CDCl_3$ at 298 K.

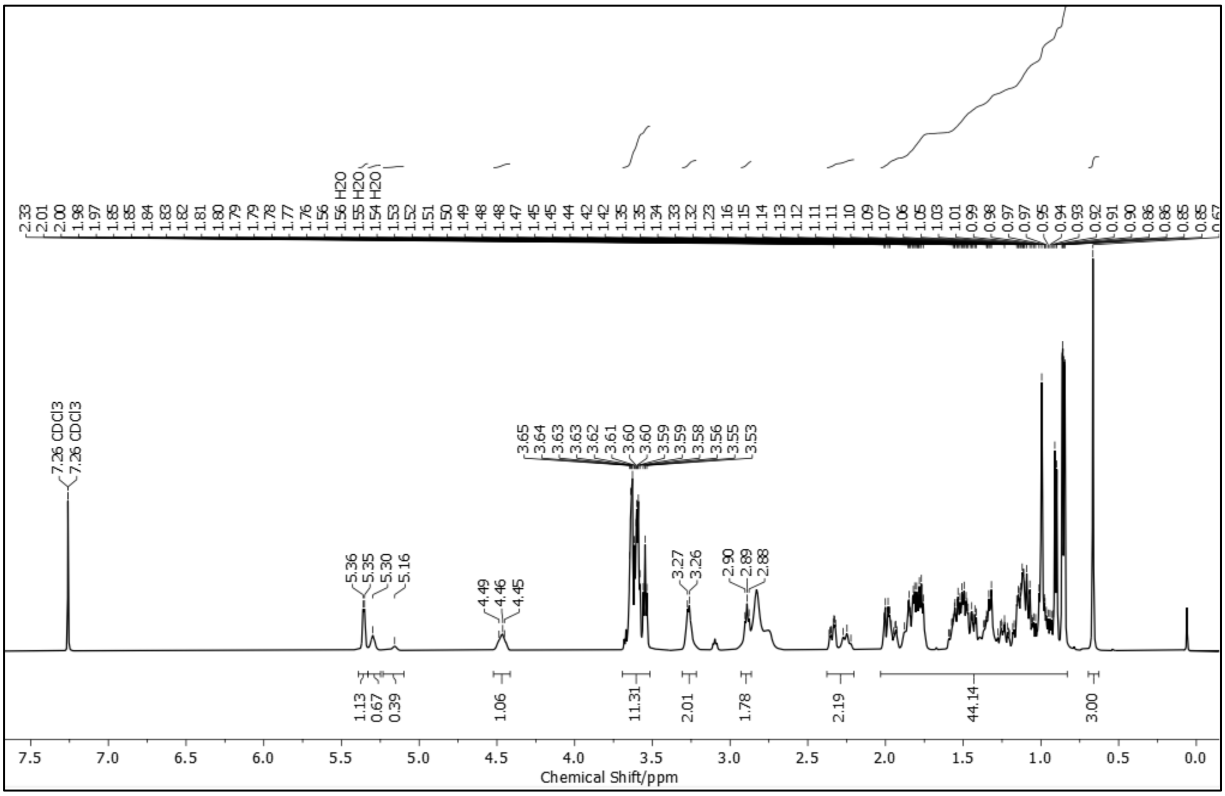

^{13}C-NMR spectrum of S2 in $CDCl_3$ at 298 K.

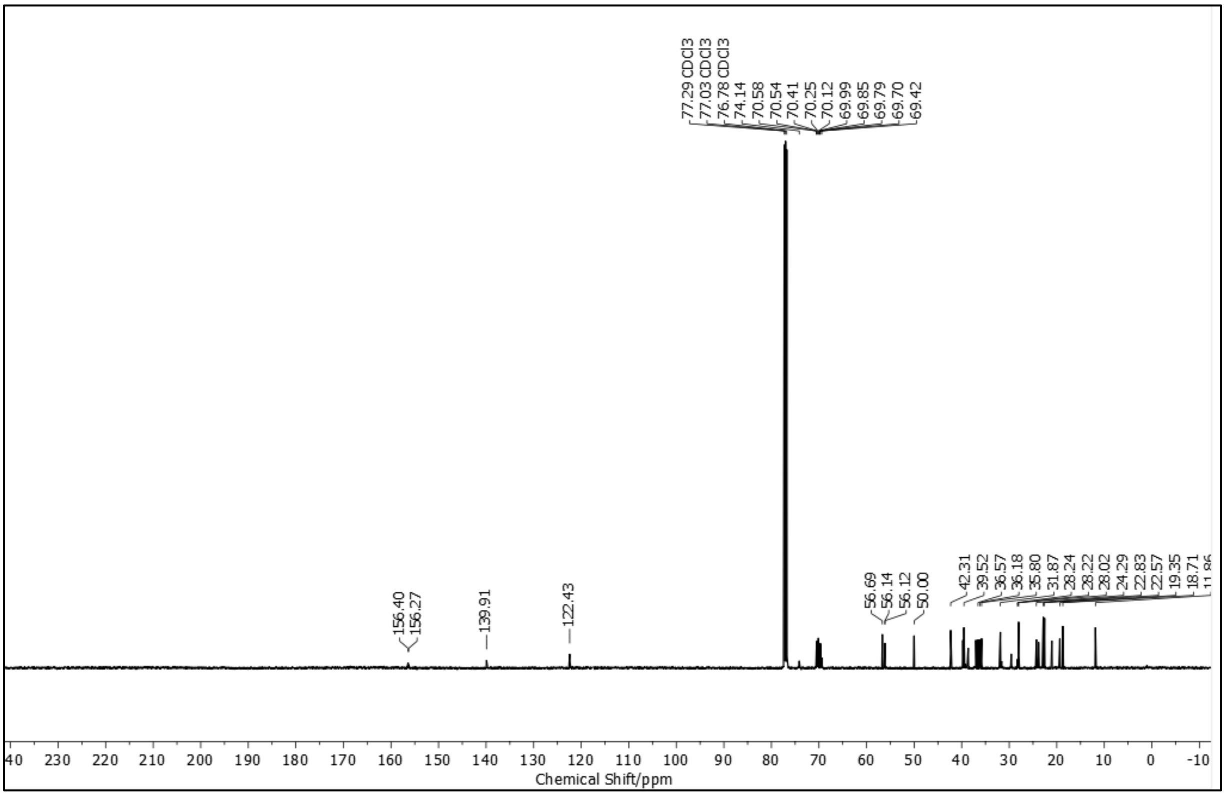

^{1}H-NMR spectrum of S4 in CD_3OD at 298 K.

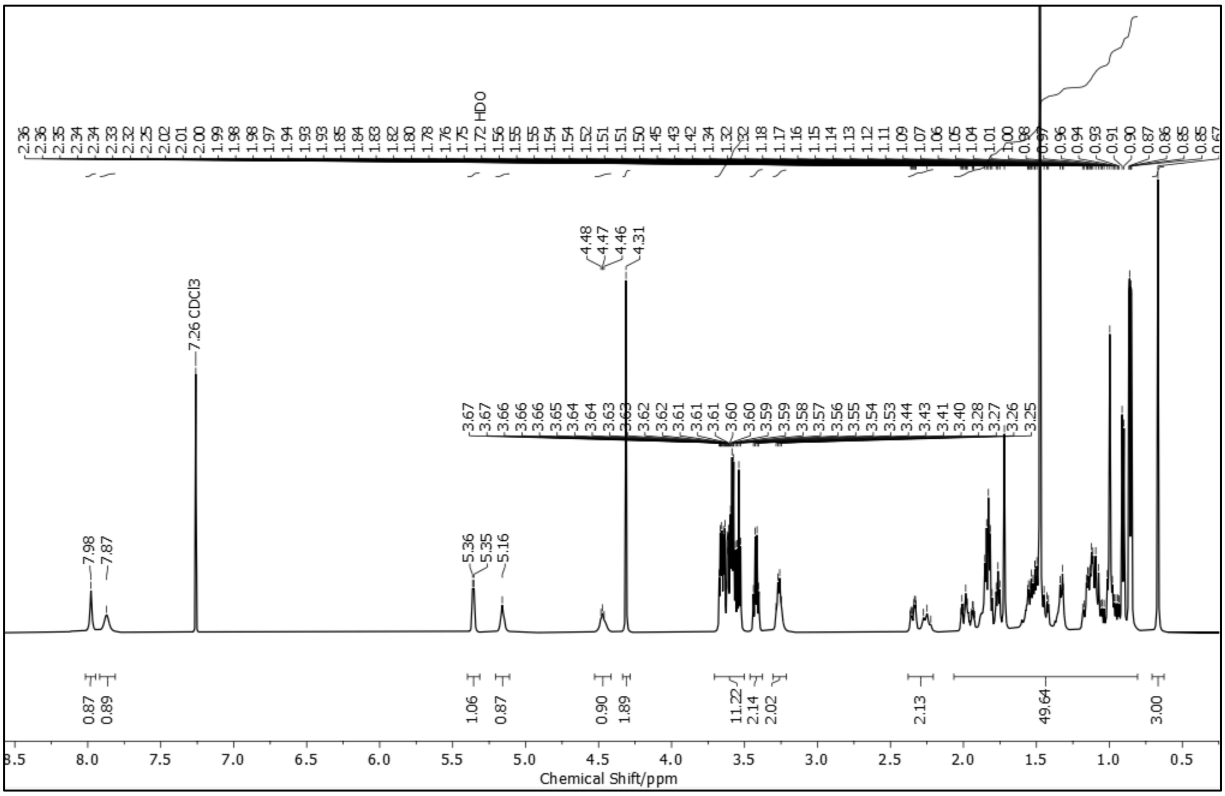

^{13}C-NMR spectrum of S4 in $CDCl_3$ at 298 K.

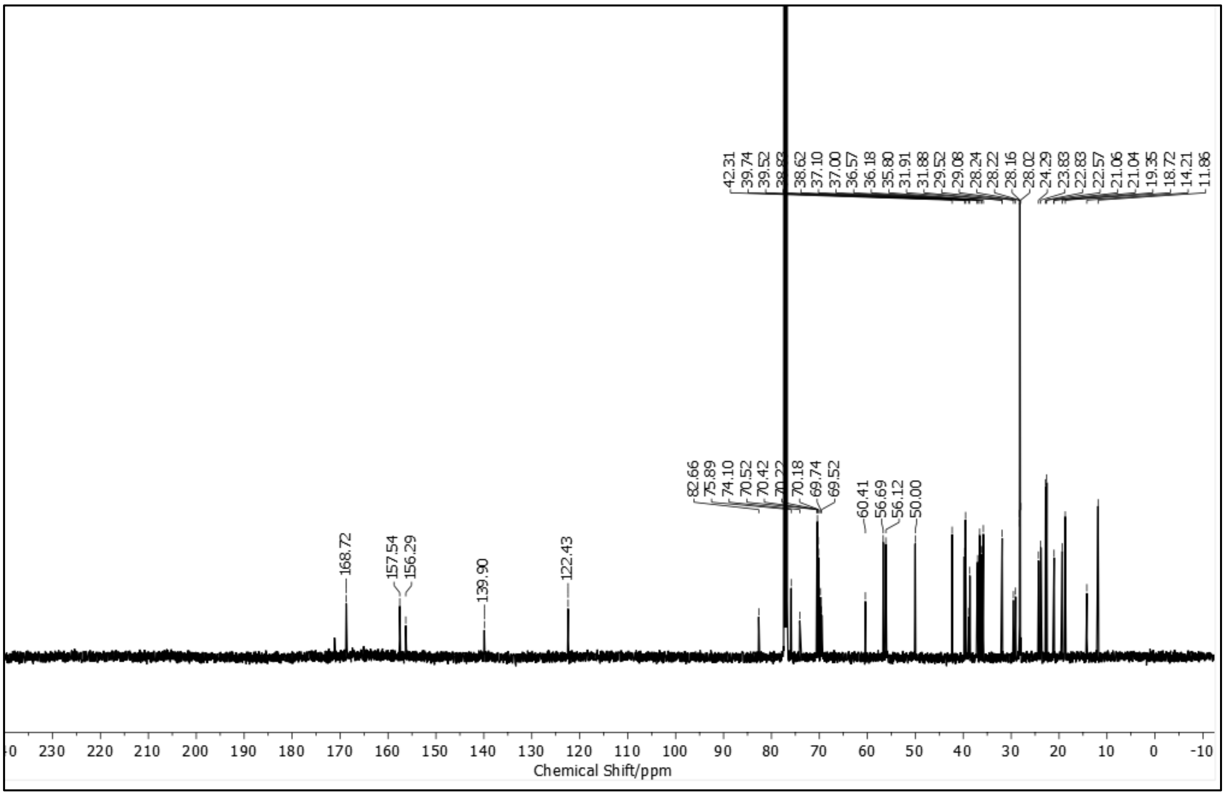

^{1}H-NMR spectrum of 1 in CD_3OD at 298 K.

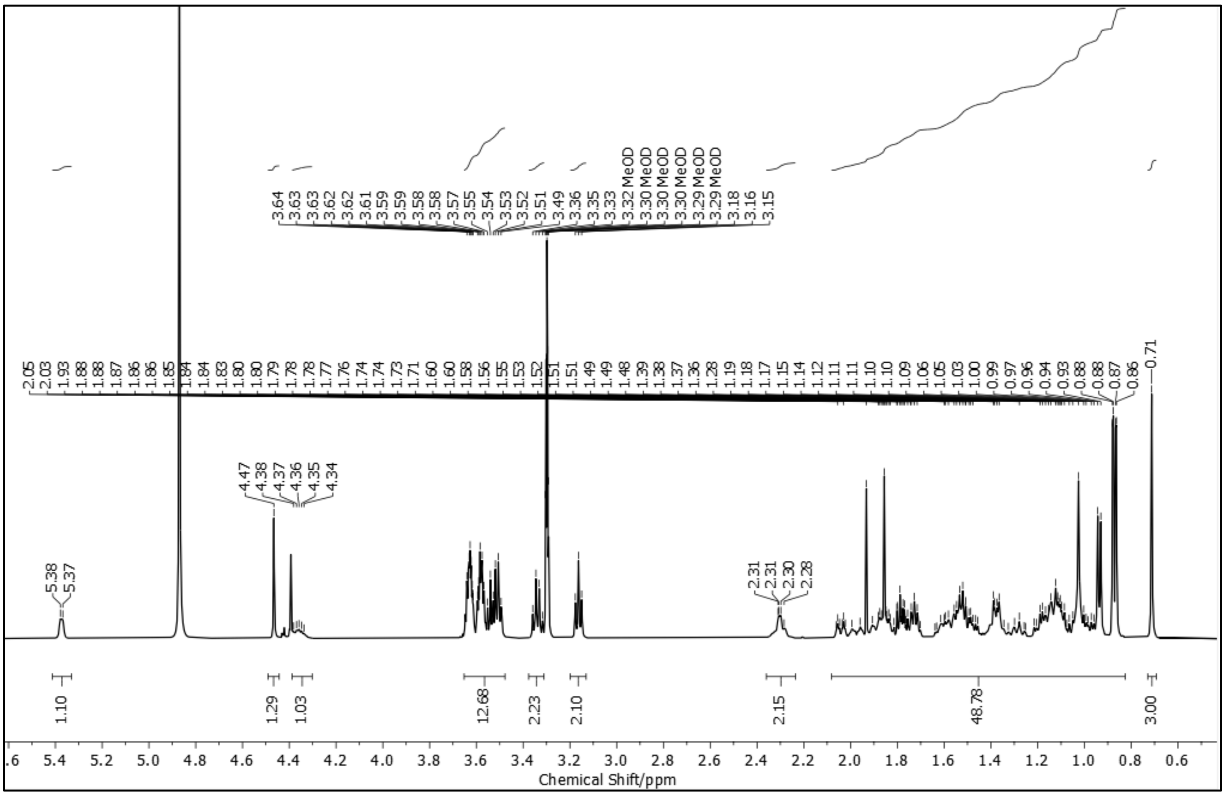

^{13}C-NMR spectrum of 1 in CD_3OD at 298 K.

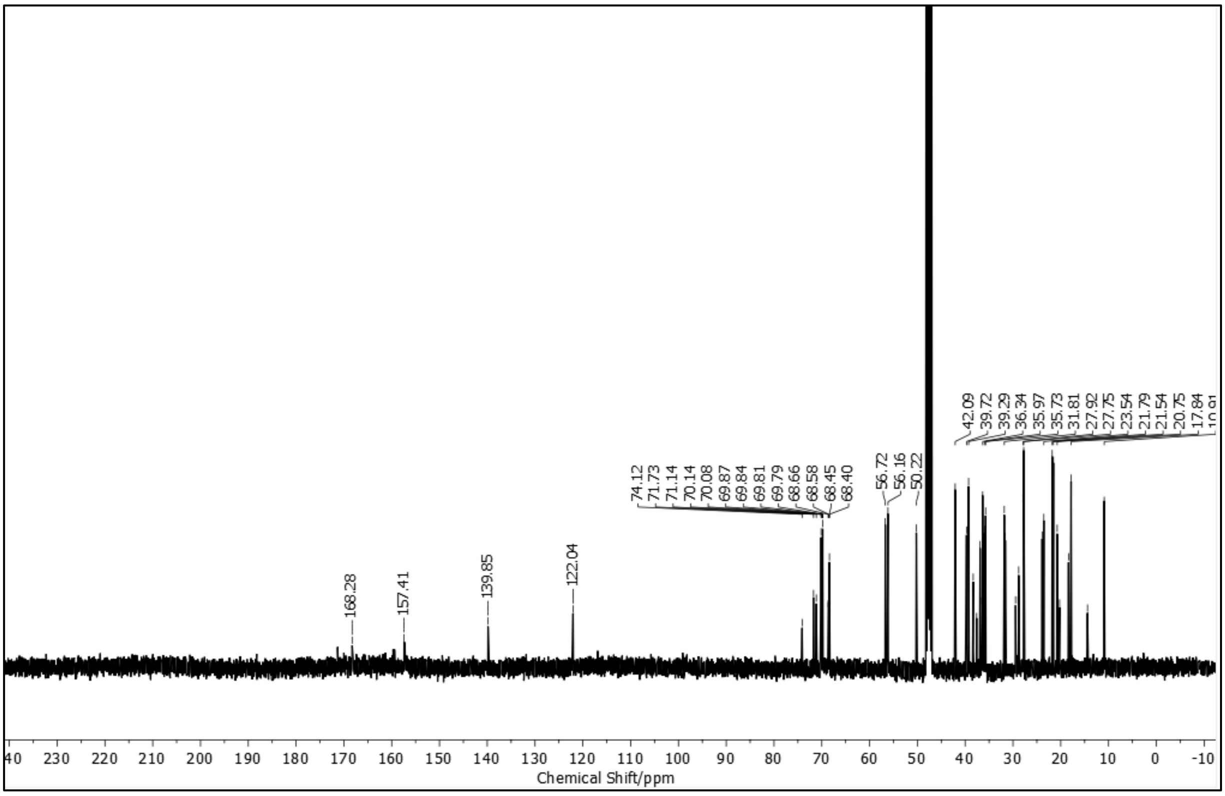

7. Enzymatic transformation of GlcNAc-PNP S18

Enzymatic transformation of S18 using combinations of β4GalT1, TcTS and α1,3-FucT

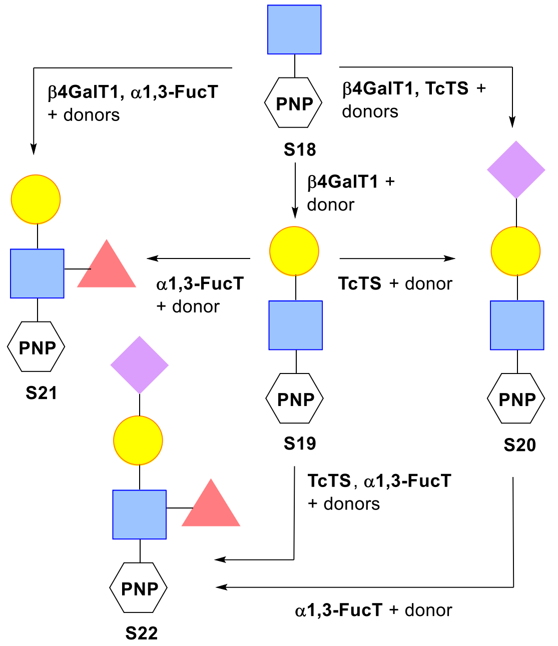

Scheme S3: Proposed enzymatic transformation of GlcNAc-PNP into oligosaccharides. Donor mixtures = UDP-Gal; 3'-SL; FKP, L-Fuc, ATP, GTP, $MgCl_2$. Sugar symbols according to SNFG.[6] PNP = *p*-nitrophenyl.

Overview

Given the successful use of β4GalT1 to transform DMPC/**2** liposomes and the failure to directly condense 3'SL with lipid **1**, it was hoped that the use of multiple glycosyltransferases would provide more complex glycolipids. Applying multienzyme synthetic sequences to synthetic sugars has been reported to give difficult-to-access bioactive oligosaccharides.[7] Such complex oligosaccharides on the surface of drug-loaded liposomes may produce highly specific targeting of particular cell types.

Combinations of three enzymes with glycosyltransferase activity were used, namely β4GalT1, TcTS and α-1,3-fucosyltransferase (α1,3-FucT). Different combinations of these enzymes could provide four oligosaccharides (Scheme S3). The methodology was first validated using soluble GlcNAc-PNP **S18**, as the *p*-nitrophenyl (PNP) chromophore permits reaction monitoring by HPLC and thereby provides quantitative data on the reaction timecourse.

The conversion of **S18** to **S20** with β4GalT1/UDP-Gal and TcTS/3'-SL (Scheme S3, right) using a "one-pot" procedure has already been reported, giving up to 70% of **S20** within an hour with concomitant 25% conversion to **S19**.[8] Product **S20** has a Neu5Ac(α2–3)Gal(β1–4)GlcNAc sequence that is similar to the Neu5Ac(α2–3)Gal(β1–4)Glc sequence found on GM_3 glycolipid.[9]

A combination of β4GalT1 and α-1,3-FucT may elaborate a GlcNAc terminus into Lewis X (Le^x), which mediates several important cellular functions in neural development and in immunity.[10] This would also be a new combination of enzymes for "one pot" liposome modification methodology (Scheme S3, left). The glycosyl donor substrate for α1,3-FucT is GDP-fucose. Although commercially available, GDP-fucose is expensive, so FKP enzyme (L-fucokinase/GDP-fucose pyrophosphorylase) was used to

convert L-fucose into GDP-Fuc, with ATP and GTP added as donors.[11] The enzymatic synthesis of Le^x-PNP was investigated by both sequential and "one-pot" approach using β4GalT1 and α1,3-FucT enzymes (Scheme S4). The sequential approach comprised an initial incubation of GlcNAc-PNP with β4GalT1 enzyme. HPLC analysis revealed a (92 ± 2)% conversion after 5 h, which reached a maximum (96 ± 1)% conversion after 16 h. After this time, the solution was incubated with α1,3-FucT enzyme. After only 3 h, the Le^x-PNP product **S21** was obtained with approximately complete conversion. However, a 16 h "one-pot" reaction, where GlcNAc-PNP **S18** is incubated with both β4GalT1 and α1,3-FucT enzymes, showed a maximum conversion onto Le^x-PNP of about (75 ± 10)% with GlcNAc-PNP **S18** was still present ((29 ± 6)%). This observation suggest the activity of β4GalT1 is compromised when mixed with α1,3-FucT and respective reagents, although the absence of intermediate **S19** suggests that α1,3-FucT activity is not significantly affected.

Sialylation of **S21** by TcTS might provide sialyl Lewis X (sLe^x), a key cell surface saccharide that plays key roles in inflammatory and immune processes.[12] In the natural biosynthetic pathway,[13] sialylation generally occurs before fucosylation,[14] so the synthesis of **S22** was investigated using either a sequential or a "one-pot" mix of β4GalT1 and TcTs enzymes, which was followed by reaction with α1,3-FucT (Scheme S3, bottom). The "one-pot"/α1,3-FucT approach involved a first step incubating with both β4GalT1 and TcTS[15] over 16 h, then the final step catalysed by α1,3-FucT for 16 h (Figure S3). Analysis after the first "one-pot" step showed the sialylation step was less effective with this batch of TcTS/3'-SL; after 16 h the relative proportions were 26% product **S20**,[16] 59% intermediate **S19** and 5% starting GlcNAc-PNP **S18**. Then the final step, catalysed by α1,3-FucT, was carried out for 16 h to give final proportions of sLe^x-PNP **S22** and Le^x-PNP **S21** of (30 ± 1)% and (67 ± 1)%, respectively. These proportions show that fucosylation of **S19** and **S20** was very effective. The sequential approach comprised an initial incubation of GlcNAc-PNP with β4GalT1 enzyme, then after 16 h the solution was incubated with TcTS for a further 16 h. Subsequently, this solution was incubated with α1,3-FucT (16 h). After 2 h, the proportions of **S22** (34 ± 13)% and **S21** (65 ± 13%) were at their highest values. These two approaches gave no significant difference in the proportions of oligosaccharides obtained, so the shorter "one-pot"/α1,3-FucT methodology was subsequently applied to DMPC/**2** liposomes.

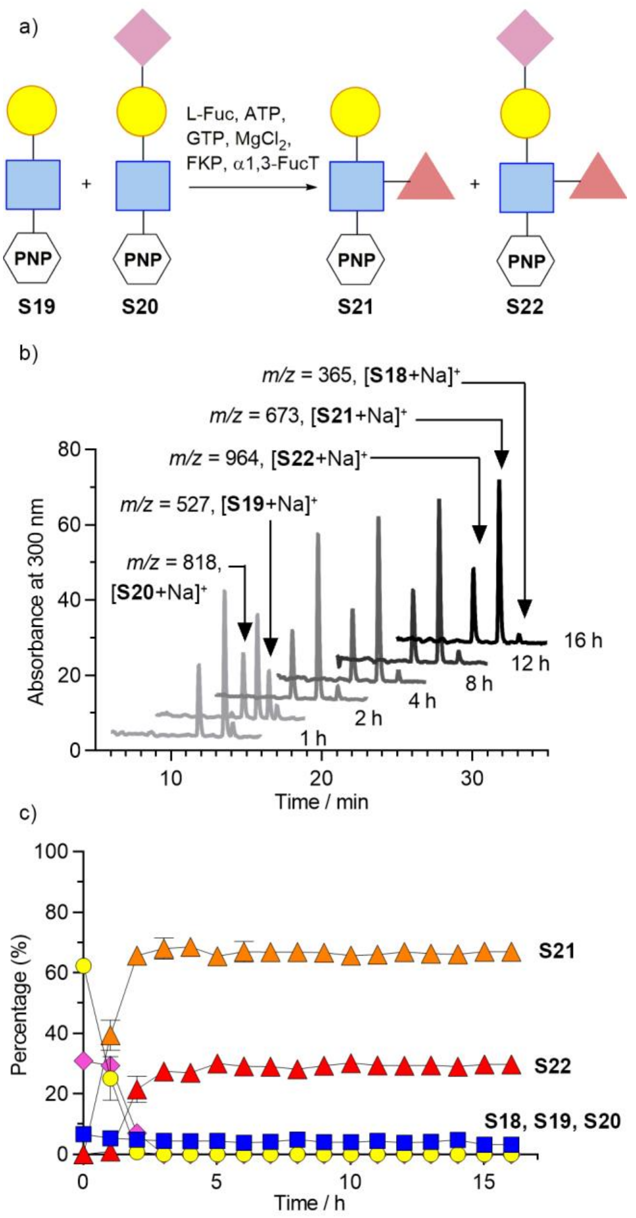

Figure S3: (a) Enzymatic transformation of a mixture of **S19** and **S20** (~60% and ~30% respectively, formed by the "one-pot" enzymatic approach) using α1,3-FucT/FKP/Fuc, affording **S21** and **S22**. (b) The reaction was monitored over 16 h by HPLC and the mass of the compounds in each peak was determined by LC-MS. (c) Time course for each compound. Each point represents the mean ± SD (*n* = 3).

Sequential and "one-pot" formation of LeX-PNP

Two approaches were investigated to afford Le^{X}-PNP **S21**, a sequential and a 'one-pot' enzymatic reaction (Scheme S4).

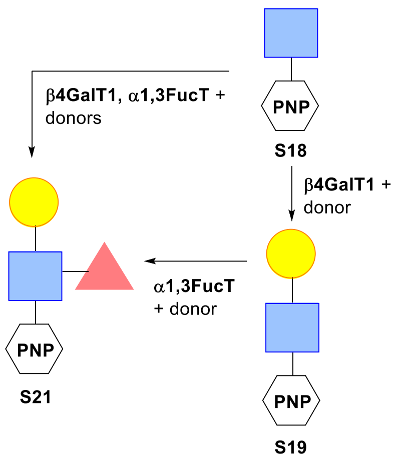

Scheme S4: Synthesis of PNP-Le^x **S21** by an enzymatic methodology. Two approaches were investigated: the sequential reaction using (a) β4GalT1 and α1,3-FucT enzymes; and the (c) 'one-pot' enzymatic transformation using β4GalT1/ α1,3-FucT enzymes in the same reaction. Sugar symbols according to SNFG.

The sequential enzymatic transformation of GlcNAc-PNP **S18** was carried out in two steps at 37 °C to afford Le^x-PNP **S21**. The first step of the reaction involved mixing GlcNAc-PNP **S18** (200 µM) with β4GalT1 (0.54 mg/mL)/UDP-Gal (10 mM) to catalyse the transfer of Gal residues and afford **S19** (Figure S3).

The second step of the sequential reaction involved using α1,3-FucT (3.7 mg/mL)/FKP (8 mg/mL)/Fuc (10 mM) to transform LacNAc-PNP **S19** into Le^x-PNP **S21**. The two expected peaks were observed in the HPLC traces (Figure S4a), namely Le^x-PNP **S21** (11.6 min) and LacNAc-PNP **S19** (12.6 min). Quantification of all compounds was carried out (Figure S4b), which shows an instant decrease of LacNAc-PNP **S19** that coincides with an increase in Le^x-PNP **S21**. After 2 h of reaction, all LacNAc-PNP **S19** is consumed and Lex-PNP **S18** was produced with 100% conversion.

The fucosylation of LacNAc-PNP **S19** using FucT was surprisingly fast and it only took a total of 2 h for the full consumption of the substrate. Therefore, the fucosylation step was repeated and the reaction analysed using shorter time points. In addition, the order that the enzymes (FKP and FucT) were added into the solution was also assessed. Specifically, FKP addition at the same time as α1,3-FucT or 20 min before α1,3-FucT enzyme (after all the other reagents) was investigated. The results obtained (Figure S5) suggested that the order that each enzyme is added into the solution is crucial to the outcome of the reaction. When FKP enzyme is added into the solution 20 min before the α1,3-FucT enzyme, the synthesis of Le^x-PNP **S21** occurs straight away (Figure S5a). However, when both enzymes are added at the same time in the solution, the enzymatic transformation of LacNAc-PNP **S19** into Le^x-PNP **S21** takes longer and at 40 min it reaches only a 50% conversion (Figure S5b). Since FKP is the accessory enzyme for the production of donor nucleotides and it is used in excess in the reaction (compared with the α1,3-FucT), it is suggested that adding the FKP enzyme into the solution 20 min before the addition of α1,3-FucT, results in a decrease of the second enzymatic transformation time from 2 h to 20 min.

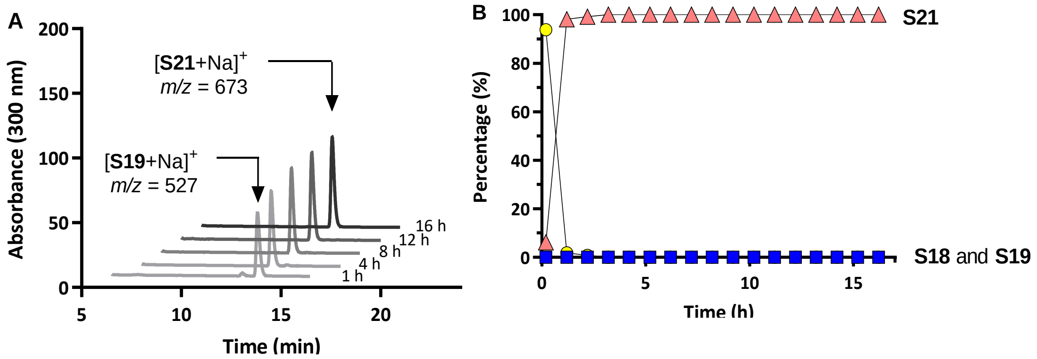

Figure S4: Enzymatic transformation of LacNAc-PNP **S19** using α1,3-FucT/FKP/Fuc to afford Le^X-PNP **S21**. A) The reaction was monitored over 16 h by HPLC and the peaks *m/z* determined by LC-MS. B) Quantification of both molecules over time. Each point represents the mean ± SD (*n* = 3).

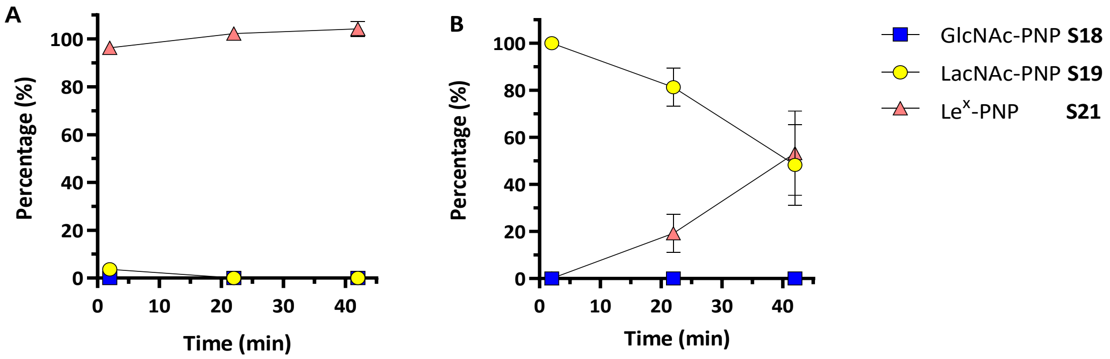

Figure S5: Effect of FKP enzyme on the conversion to Le^X-PNP **S21** when FKP enzyme is added A) 20 min before α1,3-FucT enzyme, and when it is added B) at the same time of α1,3-FucT enzyme. Each point represents the mean ± SD (*n* = 3).

The 'one-pot' enzymatic transformation was then investigated, which consists of a one-step protocol at 37 °C. GlcNAc-PNP **S18** (200 µM) was mixed with β4GalT1 (0.54 mg/mL)/UDP-Gal (10 mM) and α1,3-FucT (3.7 mg/mL)/FKP (8 mg/mL)/Fuc (10 mM) in the same reaction step, which resulted in the synthesis of Le^x-PNP **S21**. The reaction was monitored by HPLC (Figure S6a) and two peaks were identified as being Lex-PNP **S21** (11.6 min) and GlcNAc-PNP **S18** (13.0 min). The peak corresponding to LacNAc-PNP **S19** was not found during the course of the reaction, which suggests LacNAc-PNP **S19** is consumed as soon as it forms. The proportion of each molecule over time (Figure S6b) revealed a decrease of GlcNAc-PNP **S18** and an increase of Le^x-PNP **S21** in the first three hours, after which both molecules reach a plateau corresponding to proportions of 29% and 71%, respectively. The outcome of the reaction suggests that the activity of β4GalT1 enzyme is compromised by the presence of α1,3-FucT enzyme (or a reagent from the fucosylation reaction). Previously the galactosylation reaction using β4GalT1 resulted in a 96% conversion. However, in this experiment 29% of starting material GlcNAc-PNP **S18** was detected.

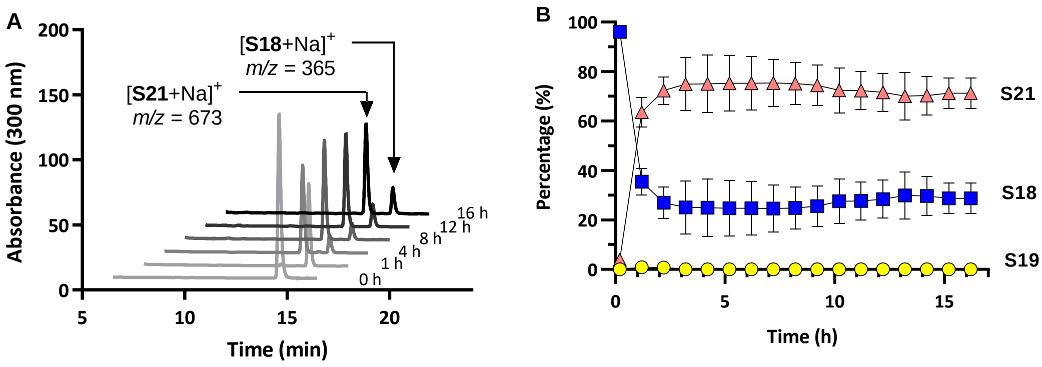

Figure S6: Enzymatic transformation of GlcNAc-PNP **S18** using β4GalT1/UDP-Gal and α1,3-FucT/FKP/Fuc to afford Le^x-PNP **S21**. A) The reaction was monitored over 16 h by HPLC and the peaks *m/z* determined by LC-MS. B) Quantification of both molecules over time. Each point represents the mean ± SD (*n* = 3).

Le^x-PNP **S21** was afforded by two methods and the results revealed that the sequential approach afforded higher proportion overall conversion to Le^x-PNP **S21** (~96% over two steps) than the 'one-pot' approach (71%, Figure S6). In addition, the 'one-pot' method also gave unreacted **S18** at the end of the experiment, which was detrimental to the Le^x-PNP **S21** yield. Also, by adding the FKP enzyme 20 min before the addition of α1,3-FucT, the reaction time of the sequential approach could be considerably decreased.

Procedures for enzymatic transformation of PNP derivatives

The enzymatic transformation of GlcNAc-PNP was monitored by reverse-phase HPLC using a 20 min gradient method ranging from 5 to 30% MeCN/IPA (4:1) in water with a flow rate of 0.5 mL/min. The eluent was monitored by setting the HPLC DAD module to record the phenyl group absorbance at 300 nm. A Macherey-Nagel Nucleosil C_{18} (150 × 4.6 mm, 5 µm) fitted with a guard column was used. The GlcNAc-PNP **S18** eluted at 13.0 min, LacNAc-PNP **S19** at 12.6 min, Neu5Ac-LacNAc-PNP **S20** at 11.4 min, sLe^x-PNP **S22** at 10.3 min and Le^x-PNP **S21** at 11.6 min. The *m/z* of each peak was analysed by LC-MS using the same method.

Enzymatic transformation using β4GalT1 enzyme

GlcNAc-PNP (100 µL of 200 µM stock in MES buffer) was mixed with UDP-Gal (30 µL, 10 mM in H_2O), bovine β4GalT1 (5 µL, 0.54 mg/mL) and $MnCl_2$ (1 µL, 1 M in H_2O), and incubated overnight at 37 °C.

Enzymatic transformation using TcTS enzyme

After an overnight incubation with β4GalT1 enzyme (as above), the solution was mixed with TcTS (5 µL, 3.91 µM), 3'-SL (5 µL, 6 mM in H_2O) and $MnCl_2$ (1 µL, 1 M in H_2O), and incubated overnight at 37 °C.

Enzymatic transformation using α1,3-FucT enzyme

After an overnight incubation with TcTS enzyme (as above), the solution was mixed with L-Fuc (5 µL, 10 mM in 100 mM Tris buffer, pH 7.5), ATP (5 µL, 10 mM in 100 mM Tris buffer, pH 7.5), GTP (40 µL, 10 mM in 100 mM Tris buffer, pH 7.5), $MgCl_2$ (1 µL, 1 M in H_2O), FKP (2 µL, 8 mg/mL) and α1,3-FucT (6 µL, 3.7 mg/mL). The solution was incubated overnight at 37 °C.

Enzymatic transformation using β4GalT1/TcTS enzymes

GlcNAc-PNP (100 µL, 200 µM, in MES buffer) was mixed with UDP-Gal (30 µL, 10 mM in H_2O), $MnCl_2$ (1 µL, 1 M in H_2O), 3'-SL (5 µL, 6 mM in H_2O), bovine β4GalT1 (5 µL, 0.54 mg/mL) and TcTS (5 µL, 3.91 µM). The solution was incubated overnight at 37 °C.

Enzymatic transformation using β4GalT1/α1,3-FucT enzymes

GlcNAc-PNP (100 µL, 200 µM, in MES buffer) was mixed with UDP-Gal (5 µL, 10 mM in H_2O), $MnCl_2$ (1 µL, 1 M in H_2O), bovine β4GalT1 (5 µL, 0.54 mg/mL), L-Fuc (5 µL, 10 mM in 100 mM Tris buffer, pH 7.5), ATP (5 µL, 10 mM in 100 mM Tris buffer, pH 7.5), GTP (40 µL, 10 mM in 100 mM Tris buffer, pH 7.5), $MgCl_2$ (1 µL, 1 M in H_2O), FKP (2 µL, 8 mg/mL) and α1,3-FucT (6 µL, 3.7 mg/mL). The solution was incubated overnight at 37 °C.

8. Liposomal studies

Liposome preparation

Standard liposome preparation: Following reported procedures, 1,2-dimyristoyl-*sn*-glycero-3-phosphocholine (DMPC, 1.8 µmol) and synthetic glycolipid (0.2 µmol, either GlcNAc glycolipid **2** or LacNAc glycolipid **13**) were dissolved in $CH_3OH/CHCl_3$ (1 mL, 1:1 v/v).[17] After solvent evaporation, the resulting thin film was suspended in MES buffer (1 mL, 50 mM, pH 7.0) forming a suspension (2 mM lipid concentration). Then the lipid suspension was extruded (Avanti Mini Extruder) through a polycarbonate membrane (100, 200 or 800 nm diameter pore size), nineteen times to afford a monodisperse suspension of liposomes (Figure S7).[18] The hydration and the extrusion steps were performed at a temperature above the gel liquid-crystalline phase transition temperature of DMPC (T_m = 24 °C).

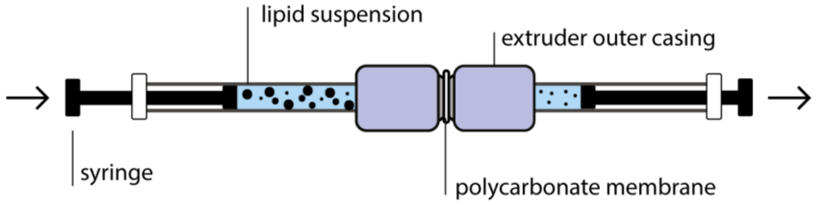

Figure S7: Schematic representation of an Avanti Mini Extruder.

To prepare "blank" DMPC liposomes, 2 µmol of DMPC was dissolved in $CH_3OH/CHCl_3$ (1 mL, 1:1 v/v). After solvent evaporation, the resulting thin film was hydrated and the lipid suspension was extruded following the standard liposome preparation method described above.

Preparation of fluorescently labelled liposomes: Labelled liposomes were prepared by mixing 1.8 µmol of DMPC, 0.2 µmol of synthetic glycolipid and 0.002 µmol (0.1% mol/mol) rhodamine DHPE (6.7 µL of a stock solution in chloroform) in $CH_3OH/CHCl_3$ (1:1 v/v). After solvent evaporation, the resulting thin film was hydrated and the lipid suspension was extruded following the standard liposome preparation method described above.

Preparation of liposomes containing cholesterol for DOX encapsulation: The liposomes were prepared by mixing DMPC (1.0 µmol), cholesterol (0.8 µmol) and glycolipid **2** (0.2 µmol) at a ratio of 49:41:10 in $CH_3OH/CHCl_3$ (1:1 v/v). After solvent evaporation, the resulting thin film was hydrated with citrate buffer (1 mL, 300 mM, pH 4.0) and the lipid suspension was extruded following the standard liposome preparation method described above.

Preparation of DOPC and DOPC/2 liposomes: DOPC liposomes were prepared by mixing 2 µmol of DOPC in $CH_3OH/CHCl_3$ (1:1 v/v), whereas DOPC/**2** liposomes were prepared by mixing 1.8 µmol DOPC and 0.2 µmol of glycolipid **2** in $CH_3OH/CHCl_3$ (1:1 v/v). After solvent evaporation, the resulting thin films were hydrated with citrate buffer (1 mL, 300 mM, pH 4.0) and the lipid suspension was extruded following the standard liposome preparation method described above.

Procedure for the enzymatic transformation of liposomes

Enzymatic transformation using β4GalT1 enzyme: The liposome suspension (100 µL) was mixed with UDP-Gal (30 µL, 10 mM in H_2O), $MnCl_2$ (1 µL, 1 M in H_2O) and bovine β4GalT1 enzyme (P08037, 5 µL of estimated 0.54 mg/mL in 50 mM MES buffer, 0.05% v/v TX-100 and 10% v/v glycerol). The solution was incubated at 37 °C overnight.

Enzymatic transformation using TcTS enzymes: The liposome suspension (100 µL) was mixed with 3'-SL (5 µL, 6 mM in H_2O), $MnCl_2$ (1 µL, 1 M in H_2O) and *Trypanosoma cruzi* trans-sialidase (TcTS, 5 µL, 3.91 µM). The solution was incubated at 37 °C overnight.

Enzymatic transformation using β4GalT1/TcTS enzymes: The liposome suspension (100 µL) was mixed with UDP-Gal (30 µL, 10 mM in H_2O), $MnCl_2$ (1 µL, 1 M in H_2O), 3'-SL (5 µL, 6 mM in H_2O), bovine β4GalT1 (5 µL, 0.54 mg/mL) and TcTS (5 µL, 3.91 µM). The solution was incubated at 37 °C overnight.

Enzymatic transformation using α1,3-FucT enzyme: The liposome suspension (100 µL) was mixed with L-Fuc (5 µL, 10 mM in 100 mM Tris buffer, pH 7.5), ATP (5 µL, 10 mM in 100 mM Tris buffer, pH 7.5), GTP (40 µL, 10 mM in 100 mM Tris buffer, pH 7.5), $MgCl_2$ (1 µL, 1 M in H_2O), 2 µL FKP enzyme (8 mg/mL) and α-1,3-fucosyltransferase (α1,3-FucT, 6 µL of 3.7 mg/mL). The solution was incubated at 37 °C overnight.

Enzymatic transformation using β4GalT1/α1,3-FucT enzymes: The liposome suspension (100 µL) was mixed with UDP-Gal (5 µL, 10 mM in H_2O), $MnCl_2$ (1 µL, 1 M in H_2O), bovine β4GalT1 (5 µL, 0.54 mg/mL), L-Fuc (5 µL, 10 mM in 100 mM Tris buffer, pH 7.5), ATP (5 µL, 10 mM in 100 mM Tris buffer pH, 7.5), GTP (40 µL, 10 mM in 100 mM Tris buffer, pH 7.5), $MgCl_2$ (1 µL, 1 M in H_2O), FKP enzyme (2 µL, 8 mg/mL) and α1,3-FucT (6 µL, 3.7 mg/mL). The solution was incubated at 37 °C overnight.

Enzymatic transformation using different sources of β4Gal transferases: The liposome suspension (100 µL) was mixed with UDP-Gal (30 µL, 10 mM in H_2O), $MnCl_2$ (1 µL, 1 M in H_2O) and β4GalT1 enzyme (5 µL, 0.54 mg/mL). Three different enzymes were tested, namely bovine β4GalT1, human β4GalT1 (P15291) and bacterial lacto-*N*-neotetraose biosynthesis glycosyltransferase LgtB (Q51116). Each solution was incubated at 37 °C overnight.

Liposome zeta potential and DLS

Suspended liposomes (0.2 mM concentration) in MES buffer (50 mM, pH 7.0) or HEPES buffer (25 mM, 150 mM NaCl, pH 7.5) and, using the diffusion barrier method,[19] were transferred to a folded capillary cell (DTS1070).

<u>DLS parameters</u>: Material DMPC, refractive index 1.400, absorption 0.001. Dispersant: water, 25 °C, viscosity 0.8872 cP, refractive index 1.330. Total of 3 measurements. Each measurement was 11 runs, 10 s each. Analysis model: general purpose. Mean particle hydrodynamic size and polydispersity index (PdI) were expressed as the mean ± standard deviation.

<u>Zeta potential parameters</u>: Material DMPC, refractive index 1.400, absorption 0.001. Dispersant: water, 25 °C, viscosity 0.8872 cP, refractive index 1.330, dielectric constant 78.5. F(Ka) model: Smoluchowski. F(Ka) value: 1.50. Equilibration time: 180 s. Total of 5 measurements, 20 runs, 180 s

delay between measurements. Voltage: 40 V (MES buffer) and 20 V (HEPE buffer). Data processing: Auto mode.

Agglutination assay using lectins

Liposome suspensions (200 nm diameter, 0.2 mM lipid concentration) were prepared in HEPES buffer (20 mM with 2 mM $CaCl_2$ and 150 mM NaCl, pH 7.5).

Agglutination assay using UV analysis by a plate reader

Liposomes were mixed with WGA or ECL lectin at different concentrations (0.1, 0.05, 0.025, 0.0125 and 0.00625 mg/mL) in a clear 96 well-plate (Corning). Samples' absorbance was recorded at 360 nm (based on the UV spectrum of the samples in the absence of lectins) over time (0, 10, 20, 30, 40 and 50 min) in a CLARIOstar plate reader (Figure S8 A/B for WGA, and C/D for ECL). Liposomes incubated in the absence of lectins were used as the control. Blank samples (liposomes in the absence of lectin) were used to subtract auto absorbance of the samples.

Agglutination assay using DLS

The liposome suspension was transferred to disposable cuvettes (ZEN0040). Each sample was analysed by DLS before and after the addition of the respective lectin (WGA or ECL) at a concentration of 0.1 mg/mL.

Agglutination assay using Fluorescence Microscopy

Liposomes were labelled with rhodamine DHPE following the protocol described in the standard procedure above. To facilitate visualisation by fluorescence microscopy, liposomes were extruded using a membrane of 800 nm pore size to afford LUVs. Liposomes were imaged before and after incubation with the respective lectin (WGA or ECL at 0.1 mg/mL) using an oil immersion 100x objective.

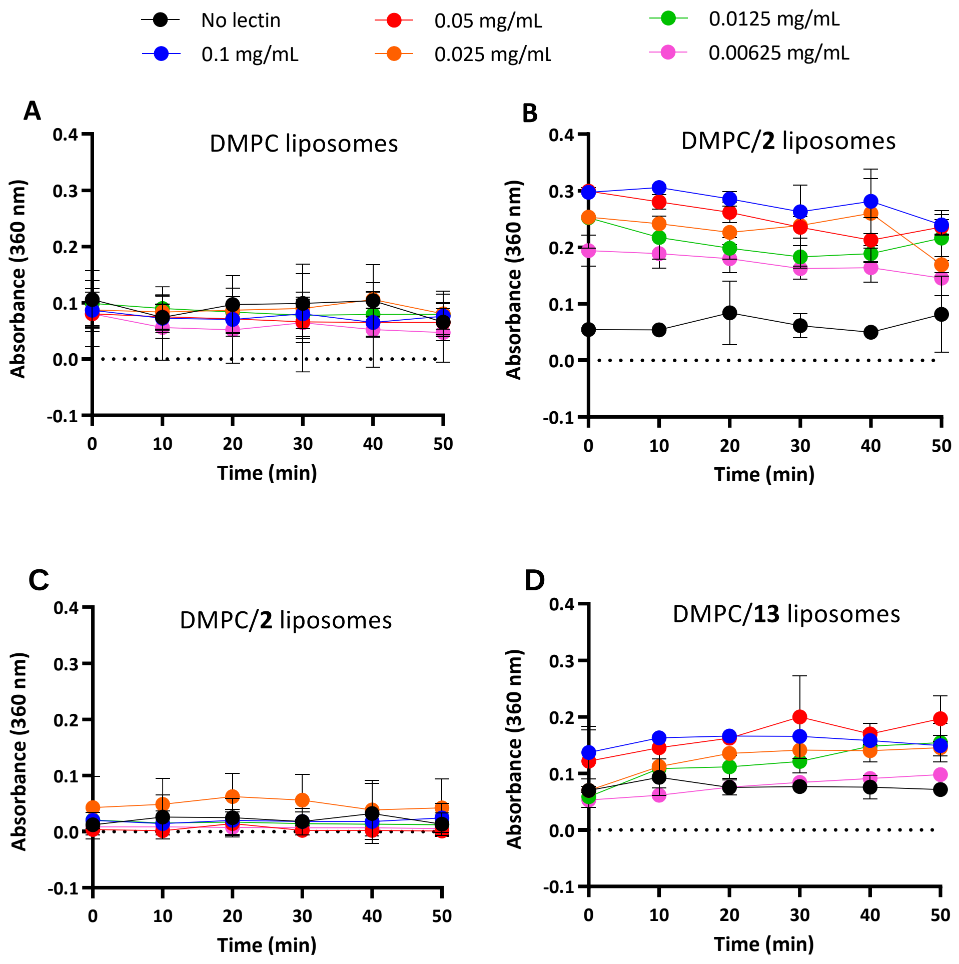

Figure S8: Agglutination assay of (a) DMPC and (b) DMPC/**2** liposomes in the presence of WGA lectin at different concentrations. (c) DMPC/**2** and (d) DMPC/**13** liposomes incubated with ECL lectin at different concentrations. Each point corresponds to the mean ± SD (*n* = 3).

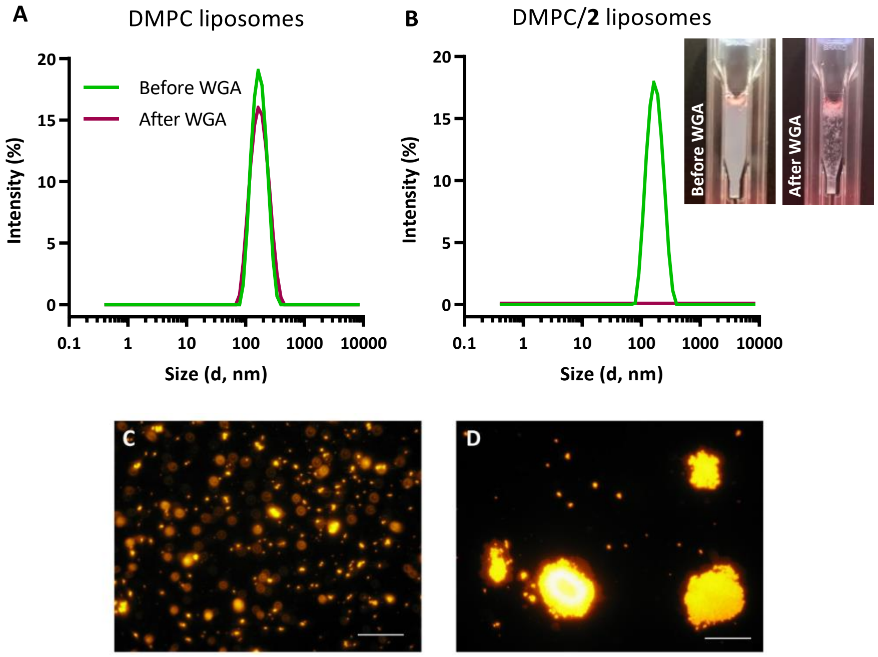

Figure S9 Effect of WGA lectin (0.1 mg/mL) on liposomes by (A, B) DLS and (C, D) fluorescence microscopy. (A) Diameter of DMPC liposomes does not change with the addition of the WGA. (B) DMPC/**2** liposomes are 159 ± 2 nm (PdI of 0.1) before addition and (3.1 ± 0.9) × 10^4 nm (PdI of 0.6) before and after addition of WGA, respectively. The peak for red line in (B) is beyond the displayed x-axis. Dispersed DMPC/**2** liposomes (labelled with rhodamine DHPE) were imaged (C) before and (D) after addition of the lectin. Scale bar 20 µm.

Measurement of galactosylation in enzymatically transformed liposomes

Quantification by galactose oxidase assay

Both DMPC/**2** and DMPC/**13** liposome formulations were analysed using the galactose oxidase assay. This assay is a simple approach for the direct measure of Gal in solution. Galactose oxidase (GOase) is a fungal enzyme able to oxidise the C-6 hydroxymethyl group of Gal, forming an aldehyde (Scheme S5). During the catalysis, oxygen is reduced to hydrogen peroxide (H_2O_2). Adding horseradish peroxidase (HRP) and 2,2'-azino-bis(3-ethylbenzothiazoline-6-sulfonic acid) diammonium salt (ABTS) into the solution results in the oxidation of the latter by the H_2O_2. Finally, a coloured ABTS positive cation $(ABTS)^+$ is formed, in a quantity proportional to the number of Gal present in solution. The GOase assay is not only suitable for the detection of free reducing Gal residues, but also to galactosyl derivatives, including Gal- or LacNAc-capped glycolipids.[20,21,22] In addition, the stereospecificity of GOase is susceptible to the orientation of the C-4 hydroxyl group, hence it does not oxidise Glc residues.[22]

Samples of DMPC/**2** and DMPC/**13** liposomes were treated according to the supplier's instructions in the galactose assay kit from Sigma. The DMPC/**2** liposomes were analysed as the control experiment. The fluorescence of the samples was measured (λ_{ex} = 535 nm and λ_{em} = 587 nm) and extrapolated from a standard curve that showed the relationship between known concentrations of synthesised glycolipid **13** and the respective fluorescence values. The results obtained (see manuscript Figure 5a)

suggest that the degree of Gal conversion in DMPC/**13** liposomes is about 16%, a comparable result to that obtained by LC-MS (see manuscript Figure 5b).

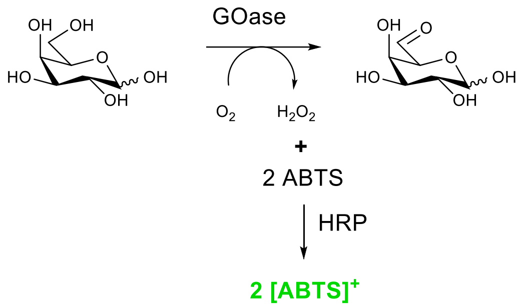

Scheme S5: Oxidation of Gal by galactose oxidase (GOase) and concomitant production of the chromophore ABTS by horse radish peroxidase (HRP).

Methodology: DMPC/**13** liposomes (2 mM concentration, 200 nm diameter) were purified by gel permeation chromatography (GPC) column (GE Healthcare Sephadex G-25 PD-10 desalting column). DMPC/**2** liposomes were analysed as the control of the experiment. Both DMPC/**2** and DMPC/**13** liposomes were mixed with reagents from the galactose assay kit® from Sigma following the supplier's instructions. Briefly, samples (50 µL) were transferred to a 96-well black plate (Greiner). To each well, 50 µL of a master reaction mixture, containing galactose assay buffer (45.6 µL), galactose probe (0.4 µL), galactose enzyme mix (2 µL) and HRP (2 µL), was added. The well plate was incubated for 30 min at 37 °C protected from light. Finally, the fluorescence intensity was measured (λ_{ex} = 535 nm/λ_{em} = 587 nm) using a plate reader. A standard curve was prepared using different known concentrations of chemically synthesised glycolipid **13** (0.2 to 1.0 nmol) plotted against the respective fluorescence intensity (Figure S10).

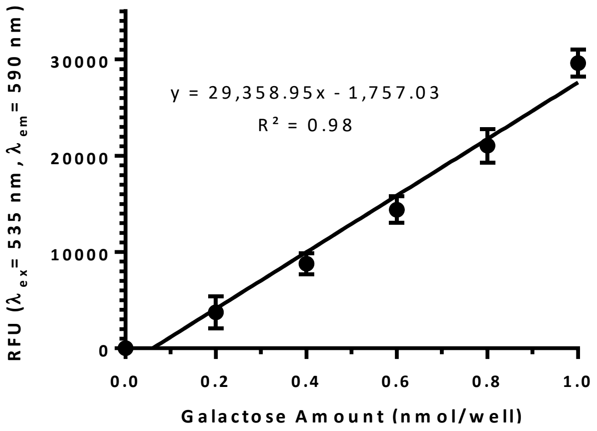

Figure S10: Standard curve for the Gal conversion by the galactose oxidase assay using known concentrations of the chemically synthesised glycolipid **13** (0.2 to 1.0 nmol) plotted against the respective fluorescence intensity. Data curve fitting (using linear regression) provided an equation that allowed to calculate the concentration of Gal in each sample (X value) using the measured fluorescence value (Y value). Results are the mean ± SD (*n* = 3).

Estimation by LC-MS

Liquid chromatography-mass spectrometry (LC-MS) was considered for the quantification of the Gal conversion since glycolipids **2** and **13** lack chromophores, which limits analysis by UV detection (*e.g.* HPLC). Mass spectrometry is highly sensitive and tolerant to mixtures; however, is not usually used as a quantitative technique because ionisation efficiencies vary among different molecules.[23] Therefore, some considerations were deliberated to carry out quantitative analysis using LC-MS, namely method optimisation (including the choice of solvents and column), reference solutions for MS calibration (purine 121 ion solution and ESI tuning mix), number of sample replicates (set up to three), analysis of blanks (MES buffer) and the interference by the enzyme (since samples were not purified). It was hoped that a standard curve showing the relationship between known concentrations of glycolipids **2** and **13** and their MS peak area could be obtained. For the purpose of the standard curve, glycolipid **13** was chemically synthesised following the same methodology used to afford glycolipid **2**.

In the context of method optimisation, samples containing DMPC/**2** and DMPC/**13** liposomes were used. Due to the glycolipid's hydrophobic nature, a C_8 column was chosen. The best compromise between the appearance of shoulders in the peak corresponding to **13** and the ionisation level of **2** was achieved using a 10 min gradient ranging from 80% to 100% of a mixture of organic solvents (50% MeCN/50% IPA supplemented with 0.1% formic acid) in water (with 0.1% formic acid) with a flow rate of 1.0 mL/min. The extracted chromatograms of [**2**+Na]$^+$ and [**13**+Na]$^+$ showed that molecule **2** eluted at 6.0 min and the molecule **13** eluted at 5.6 min. Analysis of samples containing the enzyme β4GalT1 did not show any interference with the glycolipid MS peaks.

For the quantification of Gal conversion, liposome samples containing DMPC/**2** (before enzymatic transformation) or DMPC/**13** (after enzymatic transformation) were diluted (0.02 mM) and transferred to mass spectroscopy tubes. The tubes were analysed by LC-MS using the optimised conditions. Six replicates were investigated from two independent experiments and the standard curve repeated for each one. Before enzymatic transformation, DMPC/**2** liposomes showed one peak at 6.0 min that correspond to the *m/z* of the glycolipid **2** (see manuscript Figure 5c). On the other hand, liposomes that were incubated with β4GalT1 gave rise to two peaks (see manuscript Figure 5d), namely at 6.0 min that corresponds to the *m/z* of **2** ([**2**+Na]$^+$), and at 5.6 min that corresponds to an *m/z* of **13** ([**13**+Na]$^+$). The peak area of **2** is smaller in enzymatically transformed samples DMPC/(**2**+**13**) than in DMPC/**2** suggesting a partial consumption of **2** after the enzymatic reaction. The respective peak areas were then extrapolated from the standard curve (Figure S11) and a 19% conversion to **13** was estimated.

Methodology: Aliquots (5 µL) of DMPC/**2** (before enzymatic transformation) and DMPC/(**2**+**13**) (after enzymatic transformation) liposome suspensions (0.02 mM concentration in MES buffer) were analysed by LC-MS using a 10 min gradient ranging from 80% to 100% of a mixture of organic solvents (50% MeCN/50% IPA supplemented with 0.1% formic acid) in water (with 0.1% formic acid) with a flow rate of 1.0 mL/min. Due to the glycolipid amphiphilic nature, an Agilent Zorbax Eclipse XDB C_8 column (150 × 4.4 mm, 5 µm) fitted with a guard column was used. A vial containing only buffer was used to wash the system. Analysis of samples containing the β4GalT1 enzyme did not show any interference with the glycolipid MS peaks. Molecule **2** eluted at 6.0 min and molecule **13** eluted at 5.6 min based on the extracted chromatograms of [**2**+Na]$^+$ and [**13**+Na]$^+$, respectively. The area of each

peak (in counts) was normalised as a ratio of enzymatic transformed product (EC_{1093}) in terms of non-transformed product (EC_{931}) using the **Equation S1**. The values obtained were then extrapolated from a standard curve (Figure S11) to obtain the percentage of Gal conversion in each sample. The standard curve consisted of known concentrations of glycolipid **2** mixed with the chemically synthesised glycolipid **13** (at the ratio of 0:100, 25:75, 50:50, 72:25 and 100:0), plotted against the normalised peak area from the respective extracted chromatogram. A total of two experiment were performed (each with three replicates) and for each one a new standard curve was prepared as well as standard MS calibration (using solutions of purine 121 ion solution and ESI tuning mix).

$$Normalised\ peak\ area = \frac{EC_{1093}}{(EC_{1093} + EC_{931})}$$

Equation S1

Where: EC_{931} = peak area of [**2**+Na]$^+$ (in counts) and EC_{1093} = peak area of [**13**+Na]$^+$ (in counts).

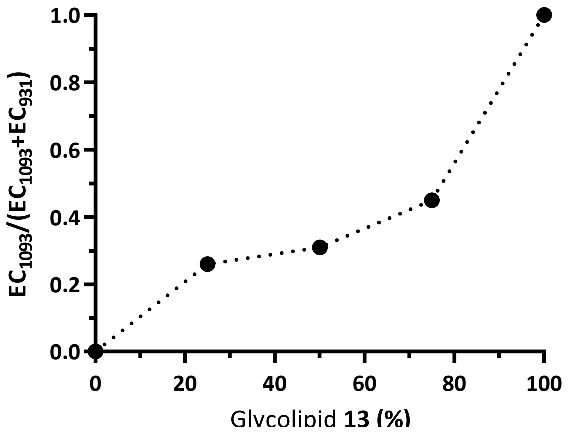

Figure S11: LC-MS standard curve consists of known concentrations of **13** mixed with glycolipid **2** (both chemically synthesised) at the ratio of 0:100, 25:75, 50:50, 72:25 and 100:0. These values were plotted against the normalised peak area from the respective extracted chromatogram using **Equation S1** above.

Quantification of available *N*-alkoxyamino groups on liposome surfaces

The extent to which this class of lipid embedded into bilayers was assessed using the 2,4,6-trinitrobenzenesulfonic acid (TNBS) assay on lipid **1**. The TNBS assay is a colorimetric test that consists of reacting TNBS with free primary amines, which result in an orange coloured trinitrophenyl derivative with absorbance at 320 nm (Scheme S6).[1] TNBS does not react with secondary (R_2NH) or tertiary (R_3N), making this assay highly specific for detecting primary (RNH_2) amines.

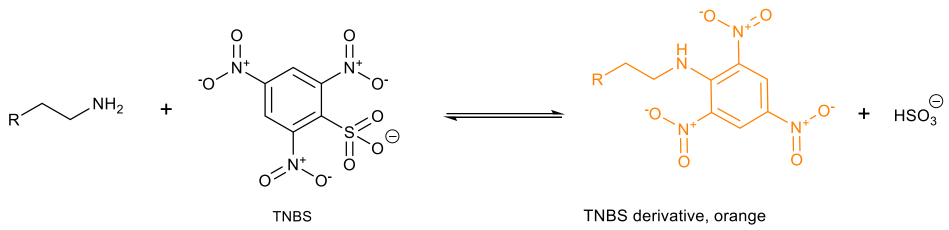

Scheme S6: Colour generating reaction in the TNBS assay.[1]

For the purpose of the experiment, liposomes (200 nm diameter) were prepared bearing amino lipid **1** because the free primary amine can be detected by the TNBS assay. The liposomal suspension was transferred to a Vivaspin®500 centrifugal concentrator (cut-off of 10 kDa) and after centrifugation, both top and bottom solutions were collected and analysed (Figure S12 A). It was expected that non-embedded lipid **1** would move to the bottom compartment of the Vivaspin while lipid **1** embedded within the bilayers would stay on top, as they should be unable to cross the filter. The collected solutions were analysed according to the TNBS methodology described by Davidenko *et al*. (see below).[24] The absorbance values obtained for each sample was extrapolated from a standard curve that consisted of the relationship between known concentrations of glycine (one primary amine per molecule) and the respective absorbance at 320 nm (see below).

The results obtained (Figure S12 C) show that the Vivaspin top compartment contained about 0.02 µmol of amine groups (100 µL at 0.2 mM), whereas on the bottom compartment no amine content was detected. Glycolipid **1** was not detected in the bottom compartment, which means that full incorporation of glycolipid **1** (0.2 mM) within the membrane bilayer of the liposomes was achieved. This result indicates that this class of synthetic *N*-(alkyloxy)amine-terminated lipid does, in fact, mix well with the l_d phase of DMPC phospholipid bilayers.

Methodology: Liposomes were prepared bearing amino groups (lipid **1**) because the free primary amine can be detected by the TNBS assay. The liposomes suspension was transferred to a Vivaspin®500 (cut-off of 10 kDa, 2.9 nm pore size) and centrifuged (4000 rpm, 20 min). It was expected that non-embedded lipid **1** would move to the bottom compartment of the Vivaspin while the molecules embedded within the bilayers would stay on top as they should be unable to cross the filter (Figure S12 A). Both top and bottom solutions were collected and analysed following the same methodology. Then 0.5 mL of freshly made 0.05% (v/v) TNBS was added to each tube, followed by a 2 h incubation at 40 °C. In order to stabilise the TNBS-complex, 1.5 mL of HCl (6 M) was added to each solution and samples were further incubated for 90 min at 60 °C. Finally, samples were diluted with 2.5 mL of distilled water. An aliquot of 100 µL of each sample was transferred to a clear 96 well-plate (Corning) and the absorbance was measured at 320 nm using a plate reader. The absorbance values obtained were extrapolated from a standard curve that consisted of glycine solution at different known concentrations (0.001 – 0.167 mmol/well) plotted against the respective absorbance (Figure S12 B). Blank samples were used to subtract auto absorbance of the samples.

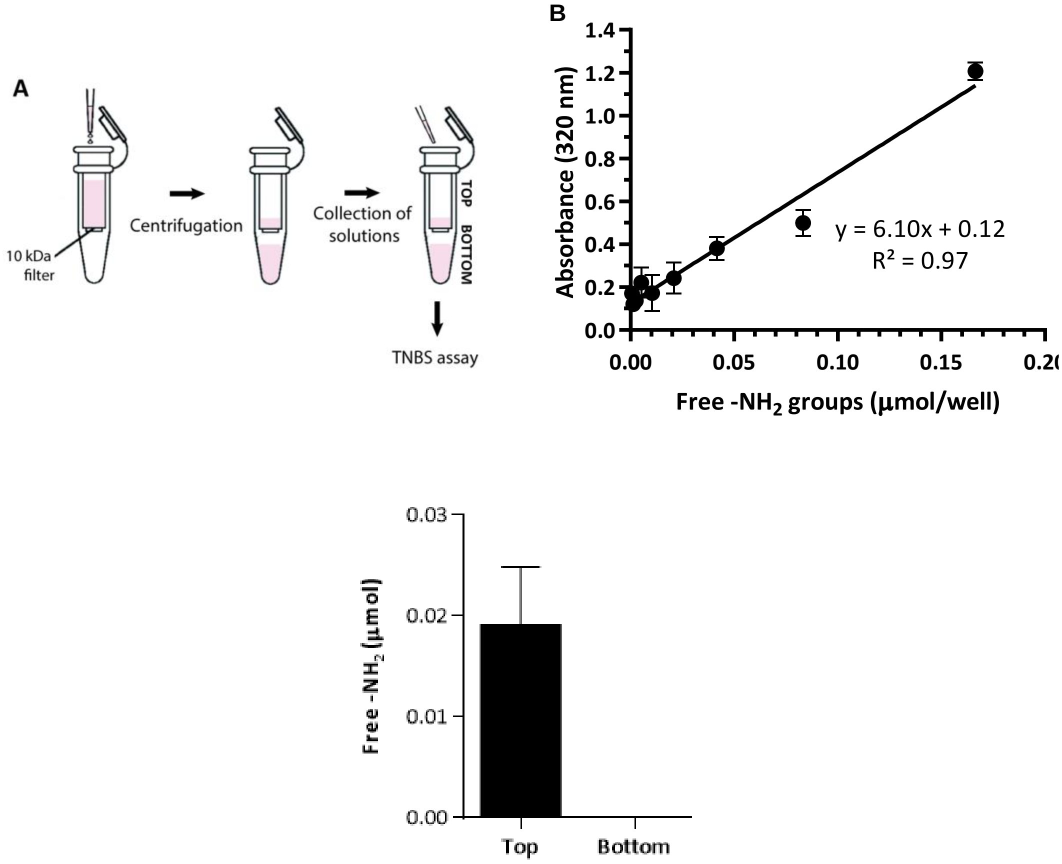

Figure S12: Quantification of non-incorporated glycolipid **1** after liposome preparation. (A) Liposomal solutions were spin down in a Vivaspin, and the top and bottom solutions were collected and analysed by the TNBS assay. (B) TNBS standard curve using known concentrations of glycine (0.001 – 0.167 µmol/well) plotted against the respective absorbance. Data curve fitting (using linear regression) provided an equation that allowed to calculate the concentration of amino groups in each sample (X value) using the measured absorbance (Y value). Data represents the mean ± SD (*n* = 4). (C) Liposomal suspensions analysed for the presence of unincorporated amine lipid by the TNBS assay. Data represents the mean ± SD (*n* = 4).

Solubility of glycolipid 2 in buffer

The alternative approach of the synthesis of **13** was considered, where the enzymatic reaction would be performed in buffer in the absence of liposomes, which we hoped would decrease the amount of steric hindrance encountered by the enzyme and increase conversion. However, mixing the glycolipid **2** in buffer (MES buffer, pH 7.0), which are the enzymatic reaction conditions, results in the formation of insoluble particles and large aggregates (40-2000 nm diameter as shown by DLS, **Figure S13**), due to the amphiphilic nature of the glycolipid. Given this observation, this approach was abandoned.

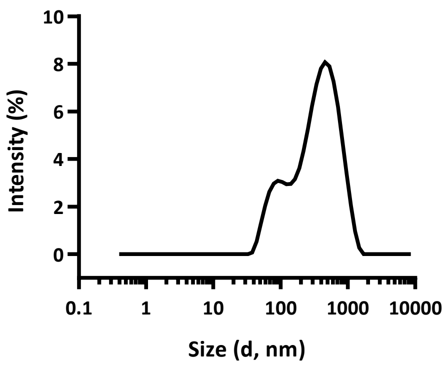

Figure S13: DLS trace of glycolipid **2** suspended in MES buffer.

Monitoring of multienzyme transformation of 2 embedded in liposomes by LC-MS.

Glycolipid **2** was mixed with phospholipids prior to vesicle formation, which results in half of the lipid becoming located in the inner leaflet of the bilayer. Therefore, at least half of glycolipid **2**, which is located in the inner face of the liposomes, will not be easily accessible to externally added regents The proportion available will depend on the rate of flip-flop of **2**; should flip-flop be slow then only half will be available.

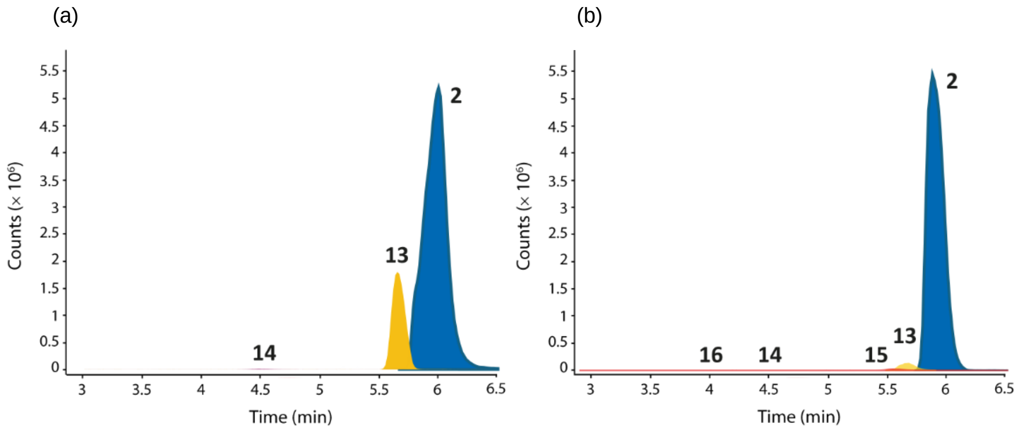

Figure S14: (a) LC-MS analysis of DMPC/**2** liposomes after incubation with β4GalT1/UDP-Gal and TcTS/3-siallyllactose in the same reaction step. (b). LC-MS analysis of DMPC/(**2**+**13**+**14**) liposomes (from the 'one-pot' β4GalT1/TcTS reaction) incubated with α1,3-FucT/FKP.

Studies of liposome stability over time

Stability is an essential parameter during liposome production, storage and administration. It includes phospholipid and ligand chemical stability, the preservation of liposome size and structure, and cargo retention.[25] In this sense, the storage stability of DMPC/**13** liposomes (in MES buffer, 0.2 mM, 200 nm diameter) was investigated at two different storage temperatures, namely 4 °C and 37 °C (to mimic cell culture temperature) over 24 days using DLS (hydrodynamic size and PdI).

Figure S15 compares the performance of DMPC, DMPC/**2** and DMPC/**13** liposomes based on their size and PdI when stored at 4 °C and 37 °C. Among these formulations, DMPC/**2** liposomes show the best performance throughout the 24 days, with consistent diameter size and PdI regardless of the temperature. In the case of DMPC/**13** liposomes, they are stable at 37 °C; however, at 4 °C their diameter changes considerably from 164 nm to 230 nm (PdI of 0.3). Similar behaviour was observed with DMPC, in which at 37 °C the liposomes were more stable than at 4 °C, although the PdI values remain unchanged in both temperatures. In summary, these results show that liposomes were more stable at 37 °C than 4 °C, which is beneficial for cell culture use. Importantly, the absence of liposomal agglomeration during the 24 days in all formulations reveals that liposomes were sufficiently charged to repel each other, despite their low ζ potentials (displayed in Table S2 and Table S3).[26]

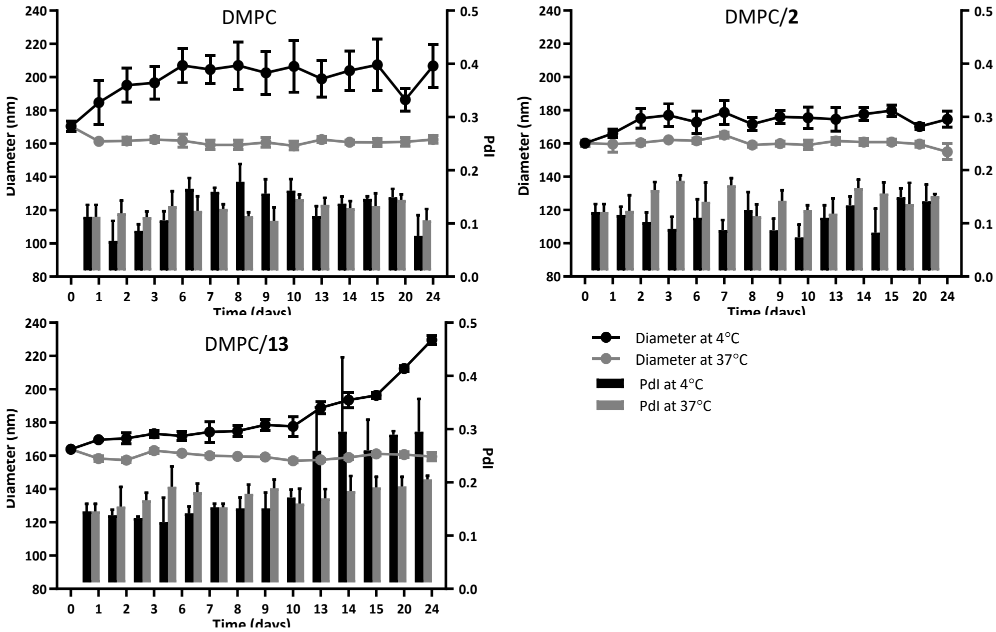

Figure S15: Stability of DMPC, DMPC/**2** and DMPC/**13** liposomes over 24 days at 4 °C and 37 °C by DLS. Results correspond to the mean ± SD (*n* = 3).

Methodology: Liposomes (DMPC, DMPC/**2** and DMPC/**13** liposomes) were prepared in MES buffer (100 nm diameter, 0.02 mM concentration) and transferred to a disposable cuvette (ZEN0040). Samples were stored at two different temperatures namely 4 °C (to mimic the storage temperature) and 37 °C (to mimic cell culture temperature). Samples were analysed by DLS and the respective hydrodynamic size and PdI recorded over 24 days. Before each measurement, each sample was pipetted up and down to resuspend any sedimented liposomes in the cuvette.

Table S2: Zeta potential of DMPC and DMPC/**2** liposomes in MES (50 mM, pH 7.0) and HEPES (25 mM, 150 mM NaCl, pH 7.5) buffer using the diffusion barrier method.[19] A total of five measurements were performed.

**Liposome Formulation**	**Zeta Potential (ζ, mV)**	
	**MES buffer**	**HEPES buffer**
DMPC liposomes	-1.75 ± 1.06	-1.28 ± 1.71
DMPC/**2** liposomes	-9.40 ± 4.19	-1.53 ± 0.67

Table S3: Zeta potential of DMPC/**13** liposomes prepared in MES and HEPES buffer using the diffusion barrier method.[19] Five measurements were performed for each sample.

**Liposome Formulation**	**Zeta Potential (ζ, mV)**	
	**MES buffer**	**HEPES buffer**
DMPC/**13** liposomes	+7.56 ± 1.12	+3.62 ± 1.12

Procedure for the active encapsulation of doxorubicin in liposomes

Active encapsulation consists of adding DOX to liposomes under an ion gradient that is established by an acidic pH inside and a basic pH outside the liposome (Figure S17). As a result, the drug crosses the bilayer and, due to the acidic environment, precipitates inside the liposome causing its entrapment. This approach has been used in the preparation of liposomes loaded with DOX for clinical use.[27]

The preparation of liposomes in acidic buffer (citrate buffer) to establish a pH gradient for the encapsulation of DOX was shown to be incompatible with the enzymatic transformation of DMPC/**2** liposomes using the β4GalT1 enzyme. Therefore DOX encapsulation would have to occur *prior* (on DMPC/**2** liposomes) to the enzymatic transformation due to the acidic conditions.

Following the literature pH gradient procedure for the active loading of DOX,[27] liposomes (DMPC and DMPC/**2**, *ca*. 100 nm diameter, 2.0 mM lipid, prepared according to details in Section 7) were prepared in citrate buffer (300 mM, pH 4.0), followed by the change of the external buffer to HEPES (25 mM with 150 mM NaCl, pH 7.5) using a gel permeation chromatography (GPC) column. DOX solution (final molar ratio of 4:1 phospholipid to the drug) was added to the liposomes and incubated for 1 h at $T>T_m$. Finally, non-encapsulated DOX was removed by a GPC column.

Liposomes were loaded at three different temperatures, namely 29 °C,[28] 40 °C and 60 °C, and the loading efficacy investigated by comparing the absorbance (DOX absorbance peak at 480 nm) of liposomes before and after the purification step (GPC column). UV-visible spectroscopy data (Figure S16) reveals complete loss of DOX absorbance after the purification step, regardless of the loading temperature. These data suggest that the encapsulation of DOX in DMPC and DMPC/**2** liposomes was unsuccessful.

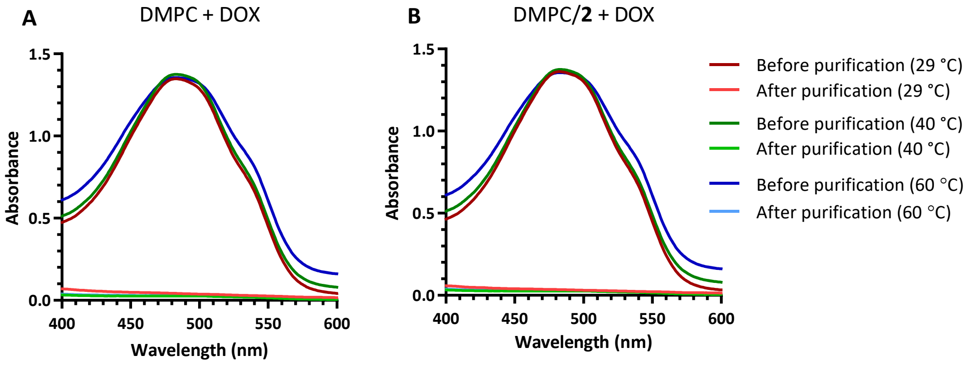

Figure S16: Absorbance profiles of (A) DMPC and (B) DPMC/**2** liposomes encapsulated with DOX by UV-visible spectroscopy before and after purification (by GPC column). Three encapsulation temperatures were tested: 29 °C, 40 °C and 60 °C.

Farzaneh *et al.* had showed the positive effect of adding cholesterol (chol) in liposomal formulations for the encapsulation of DOX.[29] In another study, Abraham *et al.* reported efficient DOX encapsulation in DMPC/chol (55:45) liposomes when incubated for 1 h at 40 °C and 60 °C.[30] Cholesterol is hydrophobic and strengthens the liposomal membranes, reducing permeability, improving stability and, significantly enhancing encapsulation efficiency and preventing drug leakage.[26,31] Since cholesterol can improve drug loading in liposomes, it was added to the current DMPC liposomal formulations and the encapsulation with DOX repeated. The two new formulations consisted of DMPC/chol (55:45) and DMPC/chol/**2** (49:41:10) liposomes. Previous work had reported DOX encapsulation in dioleoyl phosphatidylcholine (DOPC, T_m = -17 °C),[32] so DOPC and DOPC/**2** (90:10) liposomes were prepared as controls. The procedure to load DOX into liposomes included loading temperatures of 60 °C for DMPC/chol and DMPC/chol/**2** liposomes, and room temperature (20 °C) for DOPC and DOPC/**2** liposomes.

Methodology: Doxorubicin (DOX) was encapsulated into liposomes using an active loading approach (based on a pH gradient) described in the literature (Figure S17).[27] Briefly, DMPC/chol, DMPC/chol/**2**, DOPC, and DOPC/**2** liposomes (2.0 mM total lipid) were prepared in citrate buffer (1 mL, 300 mM, pH 4.0) according to the procedure in Section 7, followed by change of the external buffer to HEPES (25 mM with 150 mM NaCl, pH 7.5) using a gel permeation chromatography (GPC) column. A stock solution of DOX was prepared in the same buffer as the liposomes. An aliquot (200 µL) of DOX solution (2.5 mM) was added to the liposome solution (3.5 mL) to give a final molar ratio of *ca.* 4:1 phospholipid to the drug. Each solution was incubated for 1 h at T>T_m, namely 60 °C for DMPC/chol and DMPC/chol/**2** liposomes, and room temperature (20 °C) for DOPC and DOPC/**2** liposomes. Finally, unencapsulated DOX was removed by use of a GPC column.

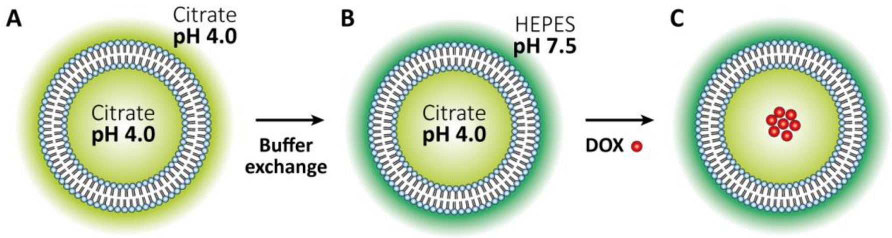

Figure S17: Active loading of DOX into liposomes by a pH gradient using the citrate loading procedure.[27] (A) Liposomes are prepared in 300 mM citrate buffer, pH 4.0, followed by a (B) buffer exchanged to 25 mM HEPES buffer (with 150 mM NaCl), pH 7.5. (C) Finally, DOX crosses the bilayer and, due to the internal acidic pH, precipitates inside the liposome, causing its entrapment

Efficiency of doxorubicin encapsulation in liposomes

The concentration of DOX in each liposomal formulation before and after purification was determined by interpolating each sample absorbance values from a standard curve (relationship between known concentrations of free DOX and the respective absorbance values, see methodology below).

The concentration of DOX detected in each liposomal formulation before and after GPC purification is shown in Figure S18. In general, a decrease in DOX concentration after purification was observed for all formulations, with DMPC/chol/**2** liposomes showing the largest decrease. By comparing concentration values before and after purification, loading efficiencies were calculated as 85% for DMPC/chol, 68% for DMPC/chol/**2**, 89% for DOPC, and 83% for DOPC/**2**. The successful loading of DOX in DMPC/chol and DMPC/chol/**2** liposomes confirms the reported benefit of cholesterol in liposomal formulations (albeit at a lower percentage than the established DOPC-based liposomes).

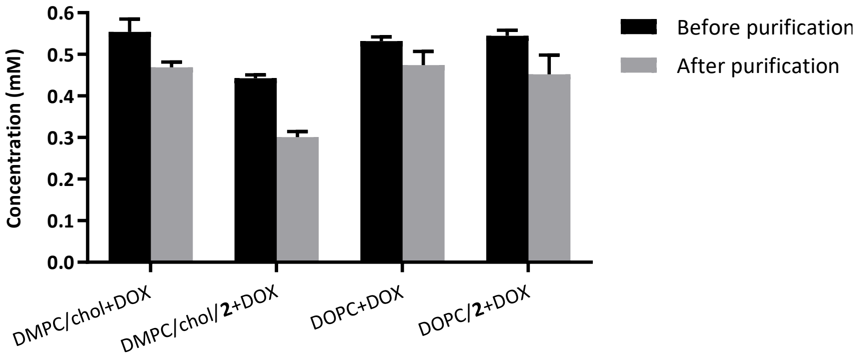

Figure S18: Concentration of DOX before and after purification (GPC column) in DMPC/chol, DMPC/chol/**2**, DOPC and DOPC/**2** liposomes. Loading efficiencies were calculated as 85% for DMPC/chol, 68% for DMPC/chol/**2**, 89% for DOPC, and 83% for DOPC/**2** liposomes. Results correspond to the mean ± SD (*n* = 4).

Once DOX is encapsulated inside liposomes, their spherical shape tends to change to an oval shape due to the precipitation of DOX in their core.[30] Therefore, the hydrodynamic size of the formulated liposomes was verified by DLS before and after loading of DOX. DMPC/chol, DMPC/chol/**2**, DOPC and

DOPC/**2** liposomes show that the hydrodynamic size was the same within the experimental error (before and after drug encapsulation), which means that no significant change in diameter size occurred due to DOX precipitation.

Methodology: Aliquots (100 µL) of each sample (before and after GPC column purification) were transferred to a clear 96 well-plate (Corning) and the absorbance measured at 480 nm. The concentration of DOX in each sample was determined by extrapolating the sample's absorbance values from a standard curve (Figure S19). The standard curve consisted of known concentrations of free DOX (0.01-0.60 mM) plotted against the respective absorbance values. Bare liposomes were considered as blank samples to account for liposomal scattering.

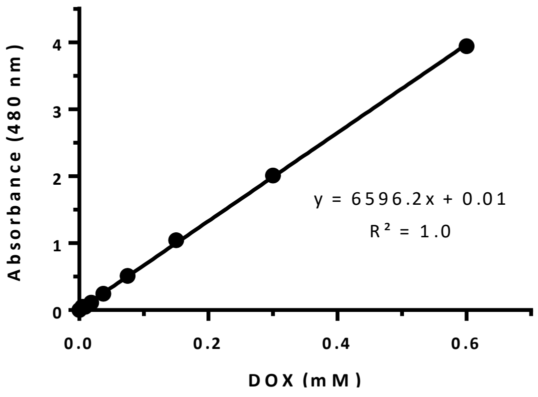

Figure S19: Standard curve using known concentrations of free DOX ranging from 0.01 to 0.60 mM plotted against the respective absorbance. Data curve fitting (using linear regression) provided an equation that allowed to calculate the concentration of DOX in each sample (X value) using the measured absorbance value (Y value). Results are the mean ± SD (n = 4).

In parallel, all liposome suspensions were analysed by DLS before and after loading of DOX and their hydrodynamic size compared. The values obtained were the same within the experimental error and no differences were observed between the diameter size of liposomes before and after encapsulation with DOX (Figure S20).

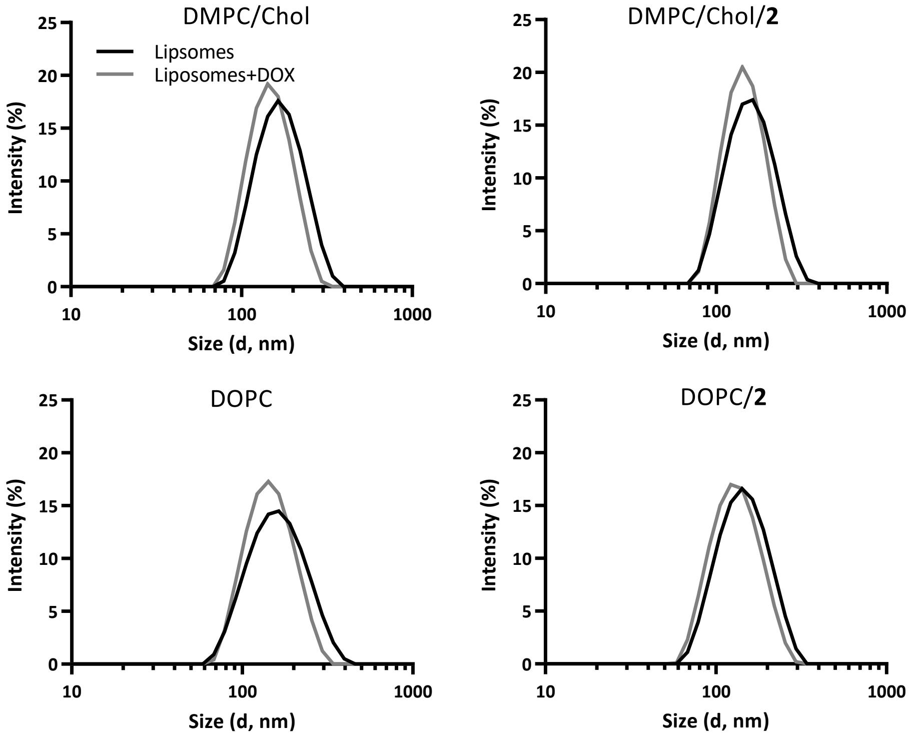

Figure S20: Size distribution of DMPC/chol, DMPC/chol/**2**, DOPC and DOPC/**2** before (black line) and after (grey line) DOX encapsulation.

Release of doxorubicin from loaded liposomes

The rate of release of doxorubicin from DMPC/chol+DOX, DMPC/chol/**2**+DOX, DOPC+DOX and DOPC/**2**+DOX liposomes was investigated using dialysis. Samples were transferred into a dialysis bag (BioDesignDialysis Tubing®, 14000 MWCO) and at specific time intervals, aliquots of the liposomal solution were collected. The absorbance of each sample was measured (λ = 480 nm) and the percentage of DOX inside the liposomes calculated. The initial percentage of loaded DOX for each liposomal formulation was set as the loading efficiency calculated earlier (Figure S18), *i.e.* 85% for DMPC/chol, 68% for DMPC/chol/**2**, 89% for DOPC, and 83% for DOPC/**2**. Free unencapsulated DOX was used as the experiment control.

The release profile of DOX is presented in Figure S21. All the liposomal formulations were able to retain their cargo for a longer period than the control (the free drug) and were retained in the dialysis bag. Although DOPC based liposomes showed the highest encapsulation efficiencies, namely 89% for DOPC and 83% for DOPC/**2**, the ability to retain their cargo was poor and at 48 h there was no DOX detected in both formulations. Since DOPC has a T_m of -17°C, DOPC and DOPC/**2** liposomes are more prone to leakage at room temperature, which might explain the premature release of their cargo. On the other hand, DMPC/chol and DMPC/chol/**2** showed the highest release of drug during the first four hours of the experiment compared with DOPC based liposomes. However, after 4 h, both DMPC/chol and DMPC/chol/**2** liposomes show only a slight decrease of encapsulated cargo and at 72 h they both reach a plateau. Final percentages of DOX inside the liposomes are 34% and 15% for DMPC/chol and DMPC/chol/**2**, respectively. Despite different initial encapsulation efficiencies, DMPC/chol and

DMPC/chol/**2** liposomes show a similar release profile throughout the experiment with a total loss of 51% and 53% of their cargo, respectively. Taken together the present results indicate that the liposomal formulations of DMPC/chol and DMPC/chol/**2** show a satisfactory performance with respect to encapsulation efficiency and rate of drug leakage.

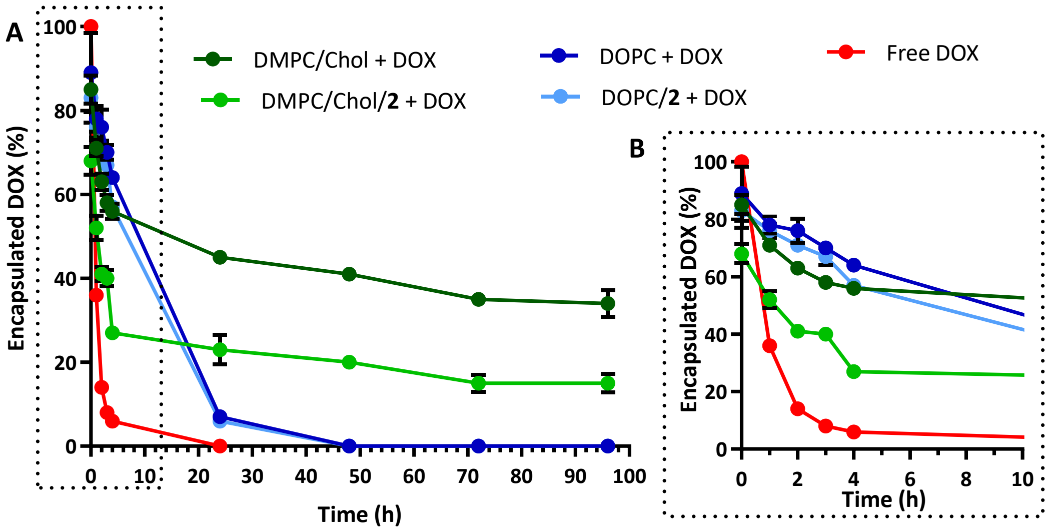

Figure S21: (A) Profile release of DOX from different liposomal formulations (DMPC/chol, DMPC/chol/**2**, DOPC and DOPC/**2** liposomes) by dialysis. Free DOX was included as control. (B) Close up of the profile release of DOX from different liposomal formulations (DMPC/chol, DMPC/chol/**2**, DOPC and DOPC/**2** liposomes) and free DOX at time intervals of 1, 2, 3 and 4 h. Results correspond to the mean ± SD (n = 3).

Methodology: DMPC/chol, DMPC/chol/**2**, DOPC and DOPC/**2** liposomes loaded with DOX were transferred into a dialysis bag (BioDesignDialysis Tubing®, 14000 MWCO). Each bag was stirred in a beaker with 200 mL of distilled water. At time intervals of 1, 2, 3, 4, 24, 48, 72 and 96 h, 350 µL of the liposomal suspension was collected from inside the dialysis bag. Aliquots (100 µL) of each sample (at different time-points) were transferred to a clear 96 well-plate (Corning) and the absorbance measured at 480 nm using a plate reader. Free unencapsulated DOX was used as the experiment control.

9. Cell toxicity assays

The cytocompatibility of enzymatically transformed liposomes was explored. DMPC, DMPC/**2** and DMPC/(**2**+**13**) liposomes were incubated with HepG2 cells, and their cell toxicity determined using the Alamar Blue® assay.

HepG2 cells (2.5×10^4 cells/cm^2, 24 h cell adhesion) were incubated with sterile DMPC, DMPC/**2** and DMPC/(**2**+**13**) liposomes (100 nm diameter) at different concentrations. After 24 h incubation, the media was replaced by media containing resazurin and incubated for further 4 h. The fluorescence of each sample was recorded (λ_{ex} = 530 nm and λ_{em} = 590 nm) and interpolated into a standard curve that showed the relationship between known concentrations of living cells (in the absence of liposomes) and the respective resorufin fluorescence values. Cells incubated in the absence of liposomes were included in the experiment as a control.

Liposomes at 5.3, 13.3 and 26.7 µg/mL do not alter cell viability (independent of the liposome formulation) since cell numbers were similar to the control (Figure S22). At higher liposomal concentrations (53.4 µg/mL), both DMPC and DMPC/**2** liposomes showed similar cell numbers with the control, but DMPC/(**2**+**13**) liposomes cause a reduction of the cell numbers to 0.75×10^4 cells/cm^2; however, it was not considered significantly different from the control by the non-paired Student's *t* test (*p* value <0.05 considered significant).[33] Based on the mean decrease of the sample (DMPC/(**2**+**13**) at 53.4 µg/mL), it is hypothesised that if higher concentrations were tested (*e.g.* 106.8 µg/mL), an even lower cell density would be observed and likely to be significant different from the control. These results indicate that all concentrations of liposomes should be suitable for cell culture experiments using HepG2 cells.

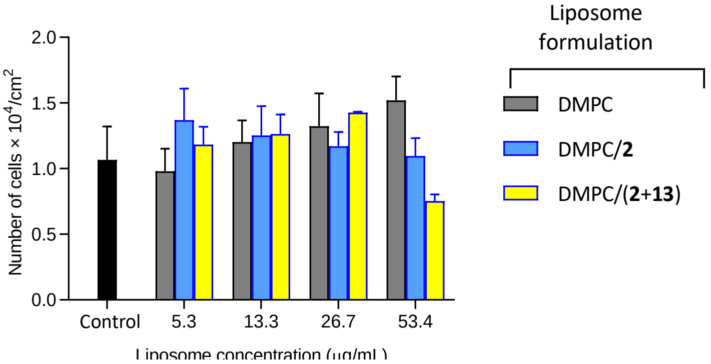

Figure S22: Viability of HepG2 cells (2.5×10^4 cells/cm^2) incubated with DMPC, DMPC/**2** and DMPC/(**2**+**13**) liposomes at 5.3, 13.3, 26.7, 53.4 µg/mL. Cells incubated in the absence of liposomes were included in the experiment as a control. Results correspond to the mean ± SD (*n* = 3). Statistical analysis by the non-paired Student's *t* test revealed no significant differences between the control and samples.

Procedure for culture of human hepatocellular carcinoma cell line (HepG2)
Cells were cultured in Eagle's minimum essential medium (EMEM) supplemented with 10% (v/v) foetal bovine serum (FBS), 2 mM L-glutamine, 1% non-essential amino acids and 1% penicillin/streptomycin solution. Cells were cultured in T25 culture flasks with 5 mL media and incubated at 37 °C with a 5% CO_2 atmosphere. Media was renewed every 48/72 h. When cells became 80% confluent, the media was removed and the flask washed with PBS in order to remove the non-adherent cells. Then, the cells were incubated with 1 mL of accutase for 5 min at 37 °C. The cell suspension was aspirated back and forth in order to separate cell aggregates. Finally, the cell suspension was distributed among three T25 flasks (1:3 subcultivation ratio).

Procedure for measuring cell viability using Alamar blue
Liposomes were prepared (100 nm) and their hydrodynamic size and monodispersity verified by DLS (Figure S23). Small liposomes were used to facilitate liposomes sterilisation by filtration (0.2 µm pore size filters), which is the common sterilisation technique used to prepare pharmaceutical products.[25] Liposomes were enzymatically transformed using β4GalT1 enzyme as described before. After reaction, β4GalT1 enzyme and UDP-Gal were removed from the liposomes solution (200 µL) by centrifugation (13000 rpm for 5 min). The supernatant was removed (150 µL) and fresh MES buffer (150 µL) was added to resuspend the pellet (by vortex). Each supernatant collected was analysed by UV-visible spectroscopy to determine the amount of enzyme and UDP-Gal present (peak at 280 nm) still present in the suspension. A total of 4 washes were necessary to remove all β4GalT1 and UDP-Gal present.

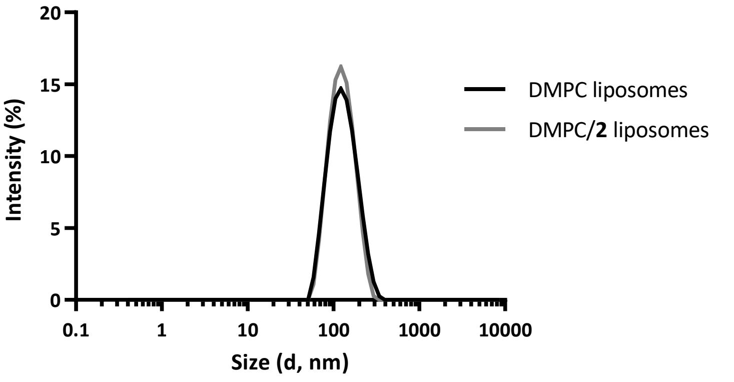

Figure S23: Hydrodynamic size of DMPC and DMPC/**2** liposomes by DLS.

HepG2 cells were seeded on a 96-well plate at 2.7 × 10^4 cells/mL and incubated for 24 h for cell adhesion. Then, sterilised liposomes at concentrations of 5.3, 13.3, 26.7 and 53.4 µg/mL were added to the cells. After 24 h incubation at 37 °C, the media was replaced by a solution of 1:10 of resazurin (0.55 mM in media without serum). The composition of fetal bovine serum (FBS) may vary between batches and, considering reproducibility of the experiment, this supplement was not included. Also, in the absence of serum, any liposomal interactions with albumin and other serum components are prevented. Cell proliferation is also likely to be prevented.[34] The plate was incubated for 4 h at 37 °C, followed by the transfer of 200 µL of cell media to a 96-well black plate (Greiner). The fluorescence of each well was recorder (λ_{ex} = 530 nm and λ_{em} = 590 nm). In parallel, a standard curve was prepared

using known concentrations of cells (0-2.7 × 10^4 cells/mL) incubated in the absence of liposomes plotted against fluorescence intensity (Figure S24).

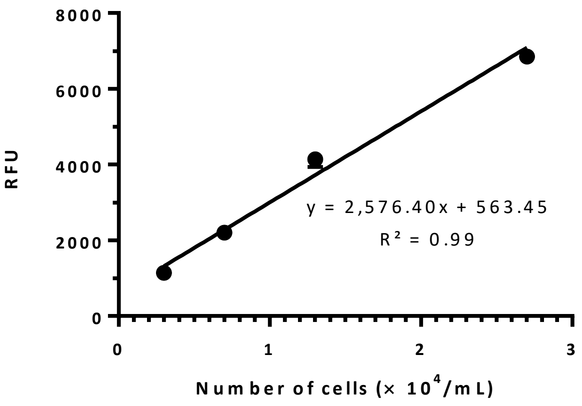

Figure S24: Cell viability standard curve using know concentrations of cells (0-2.7 × 10^4 cells/mL) incubated in the absence of liposomes plotted against fluorescence intensity. Data curve fitting (using linear regression) provided an equation that allowed to calculate the concentration of cells in each sample (X value) using the measured fluorescence value (Y value). Results are the mean ± SD (n = 3).

10. References

1. F. Edwards-Lévy, M.-C. Andry and M.-C. Lévy, *Int. J. Pharm.*, 1993, **96**, 85-90.
2. (a) C. McCallion, A. D.; Peters, A. Booth, K. Rees-Unwin, J. A. Adams, R. Rahi, A. Pluen, C. Hutchinson, S. J. Webb and J. Burthem, *Blood Adv*. 2019, **3**, 2069-2081. (b) D. Thakar, E. Migliorini, L. Coche-Guerente, R. Sadir, H. Lortat-Jacob, D. Boturyn, O. Renaudet, P. Labbe and R. P. Richter, *Chem. Commun*., 2014, **50**, 15148-15151
3. C. P. Leamon, United States of America Patent, 2005 US-6858226-B2
4. C. Booth, R. J. Bushby, Y. Cheng, S. D. Evans, Q. Liu and H. Zhang, *Tetrahedron*, 2001, **57**, 9859- 9866.
5. O. R. Baudendistel, D. E. Wieland, M. S. Schmidt and V. Wittmann, *Chem. Eur. J.*, 2016, **22**, 17359-17365.
6. (a) A. Varki, R. D. Cummings, M. Aebi, N. H. Packer, P. H. Seeberger, J. D. Esko, P. Stanley, G. Hart, A. Darvill, T. Kinoshita, J. J. Prestegard, R. L. Schnaar, H. H. Freeze, J. D. Marth, C. R. Bertozzi, M. E. Etzler, M. Frank, J. F. Vliegenthart, T. Lütteke, S. Perez, E. Bolton, P. Rudd, J. Paulson, M. Kanehisa, P. Toukach, K. F. Aoki-Kinoshita, A. Dell, H. Narimatsu, W. York, N. Taniguchi and S. Kornfeld, *Glycobiology*, 2015, **25**, 1323-1324. (b) S. Neelamegham, K. Aoki-Kinoshita, E. Bolton, M. Frank, F. Lisacek, T. Lütteke, N. O'Boyle, N. H. Packer, P. Stanley, P. Toukach, A. Varki, R. J. Woods and The SNFG Discussion Group, *Glycobiology*, 2019, **29**, 620-624.
7. (a) Z. Wang, Z. S. Chinoy, S. G. Ambre, W. Peng, R. McBride, R. P. de Vries, J. Glushka, J. C. Paulson and G.-J. Boons, *Science*, 2013, **341**, 379-383. (b) L. Li, Y. Liu, C. Ma, J. Qu, A. D. Calderon, B. Wu, N. Wei, X. Wang, Y. Guo, Z. Xiao, J. Song, G. Sugiarto, Y. Li, H. Yu, X. Chen and P. G. Wang, *Chem. Sci*., 2015, **6**, 5652-5661.
8. F. L. Craven, J. Silva, M. D. Segarra-Maset, K. Huang, P. Both, J. E. Gough, S. L. Flitsch and S. J. Webb, *Chem. Comm*. 2018, **54**, 1347-1350.
9. (a) M. A. Ferrero-García , S. E. Trombetta , D. O. Sánchez , A. Reglero , A. C. C. Frasch and A. J. Parodi , *Eur. J. Biochem*., 1993, **213**, 765-771. (b) C.-L. Schengrund, *Trends Biochem. Sci.*, 2015, **40**, 397-406.
10. E. Hennen and A. Faissner, *Int. J. Biochem. Cell Biol.*, 2012, **44**, 830-833.
11. W. Wang, T. Hu, P. A. Frantom, T. Zheng, B. Gerwe, D. Soriano del Amo, S. Garret, R. D. Seidel III and P. Wu, *Proc. Natl. Acad. USA*, 2009, **106**, 16096-16101.

12. (a) C. Chantarasrivong, A. Ueki, R. Ohyama, J. Unga, S. Nakamura, I. Nakanishi, Y. Higuchi, S. Kawakami, H. Ando, A. Imamura, H. Ishida, F. Yamashita, M. Kiso and M. Hashida, *Mol. Pharmaceutics*, 2017, **14**, 1528-1537. (b) F. Jin and F. Wang, *Glycoconj. J.* 2020, **37**, 277-291.
13. (a) N. Mondal, B. Dykstra, J. Lee, D. J. Ashline, V. N. Reinhold, D. J. Rossi and R. Sackstein, *J. Biol. Chem*. 2018, **293**, 7300–7314. (b) E. H. Holmes, G. K. Ostrander and S.-i. Hakomori, *J. Biol. Chem*., 1986, **261**, 3737-3743.
14. G. Sugiarto, K. Lau, H. Yu, S. Vuong, V. Thon, Y. Li, S. Huang and X. Chen, *Glycobiology* 2011, **21**, 387–396.
15. S. Kaloo, "*Glycosylation of Carbohydrates by Glycosyltransferases*", PhD thesis, The University of Manchester, 2009.
16. This is somewhat less than the *ca*. 70% conversion achieved previously and was also reflected in a greater proportion of the galactosylated intermediate **13**, which suggests lower activity for this batch of TcTS.
17. F. L. Craven, J. Silva, M. D. Segarra-Maset, K. Huang, P. Both, J. E. Gough, S. L. Flitsch and S. J. Webb, *ChemComm*, 2018, **54**, 1347-1350.
18. R. C. MacDonald, R. I. MacDonald, B. P. M. Menco, K. Takeshita, N. K. Subbarao and L. Hu, *Biochim. Biophys. Acta*, 1991, **1061**, 297- 303.
19. J. C. W. Corbett, M. T. Connah and K. Mattison, *Electrophoresis*, 2011, **32**, 1787-1794.
20. M. Fortelius and P. Mattjus, *Chem. Phys. Lipids*, 2006, **142**, 103-110.
21. E. M. Goudsmit, F. Matsuura and D. A. Blake, *J. Biol. Chem.*, 1984, **259**, 2475-2878.
22. R. Paukner, P. Staudigl, W. Choosri, D. Haltrich and C. Leitner, *Protein Expr. Purif.*, 2015, **108**, 73-79.
23. J. J. Pitt, *Clin. Biochem. Rev.*, 2009, **30**, 19-34.
24. N. Davidenko, C. F. Schuster, D. V. Bax, N. Raynal, R. W. Farndale, S. M. Best and R. E. Cameron, *Acta Biomater.*, 2015, **25**, 131-142
25. N. Monteiro, A. Martins, R. L. Reis and N. M. Neves, *J. R. Soc. Interface*, 2014, **11**, 1-24.
26. C. I. Nkanga, A. M. Bapolisi, N. I. Okafor and R. W. M. Krause, in *Liposomes - Advances and Perspectives*, ed. A. Catala, intechopen, 2019, vol. 3.
27. S. A. Abraham, D. N. Waterhouse, L. D. Mayer, P. R. Cullis, T. D. Madden and M. B. Bally, *Methods Enzymol.*, 2005, **391**, 71-97.
28. G. Niu, B. Cogburn and J. Hughes, in *Cancer Nanotechnology, Methods in Molecular Biology*, eds. S. R. Grobmyer and B. M. Moudgil, Springer Science, 2010, vol. 624, pp. 211-219
29. H. Farzaneh, M. E. Nik, M. Mashreghi, Z. Saberi, M. R. Jaafari and M. Teymouri, *Int. J. Pharm.*, 2018, **551**, 300–308.
30. S. A. Abraham, K. Edwards, G. Karlsson, S. MacIntosh, L. D. Mayer, C. McKenzie and M. B. Bally, *Biochim. Biophys. Acta*, 2002, **1565**, 41-54.
31. C. Kirby, J. Clarke and G. Gregoriadis, *Biochem. J.*, 1980, **186**, 591-598.
32. C. McCallion, A. D. Peters, A. Booth, K. Rees-Unwin, J. Adams, R. Rahi, A. Pluen, C. V. Hutchinson, S. J. Webb and J. Burthem, *Blood Adv.*, 2019, **3**, 2069-2081.
33. A. Field, *Discovering Statistics using SPSS*, 2nd edn., SAGE 2005.
34. V. López-Dávila, T. Magdeldin, H. Welch, M. V. Dwek, I. Uchegbu and M. Loizidou, *Nanomedicine*, 2016, **11**, 331-344.
